# A Physiologically Based Pharmacokinetic and Pharmacodynamic Model of the CYP3A4 Substrate Felodipine for Drug–Drug Interaction Modeling

**DOI:** 10.3390/pharmaceutics14071474

**Published:** 2022-07-15

**Authors:** Laura Maria Fuhr, Fatima Zahra Marok, Maximilian Mees, Felix Mahfoud, Dominik Selzer, Thorsten Lehr

**Affiliations:** 1 Department of Clinical Pharmacy, Saarland University, 66123 Saarbrücken, Germany; laura.fuhr@uni-saarland.de (L.M.F.); fatima.marok@uni-saarland.de (F.Z.M.); max.mees@prepair.de (M.M.); dominik.selzer@uni-saarland.de (D.S.); 2 Department of Internal Medicine III (Cardiology, Angiology, Intensive Care Medicine), Saarland University Medical Center and Saarland University Faculty of Medicine, 66421 Homburg, Germany; felix.mahfoud@uks.eu; 3 Institute for Medical Engineering and Science, Massachusetts Institute of Technology, Cambridge, MA 02139, USA

**Keywords:** physiologically based pharmacokinetic (PBPK) modeling, pharmacodynamics, felodipine, drug–drug interactions (DDIs), cytochrome P450 3A4 (CYP3A4)

## Abstract

The antihypertensive felodipine is a calcium channel blocker of the dihydropyridine type, and its pharmacodynamic effect directly correlates with its plasma concentration. As a sensitive substrate of cytochrome P450 (CYP) 3A4 with high first-pass metabolism, felodipine shows low oral bioavailability and is susceptible to drug–drug interactions (DDIs) with CYP3A4 perpetrators. This study aimed to develop a physiologically based pharmacokinetic/pharmacodynamic (PBPK/PD) parent–metabolite model of felodipine and its metabolite dehydrofelodipine for DDI predictions. The model was developed in PK-Sim^®^ and MoBi^®^ using 49 clinical studies (94 plasma concentration–time profiles in total) that investigated different doses (1–40 mg) of the intravenous and oral administration of felodipine. The final model describes the metabolism of felodipine to dehydrofelodipine by CYP3A4, sufficiently capturing the first-pass metabolism and the subsequent metabolism of dehydrofelodipine by CYP3A4. Diastolic blood pressure and heart rate PD models were included, using an *E_max_* function to describe the felodipine concentration–effect relationship. The model was tested in DDI predictions with itraconazole, erythromycin, carbamazepine, and phenytoin as CYP3A4 perpetrators, with all predicted DDI AUC_last_ and C_max_ ratios within two-fold of the observed values. The model will be freely available in the Open Systems Pharmacology model repository and can be applied in DDI predictions as a CYP3A4 victim drug.

## 1. Introduction

The dihydropyridine felodipine is used for the treatment of hypertension [1,2,3]. By blocking calcium channels, mainly in vascular smooth muscles, felodipine causes a reduction in vascular resistance, which subsequently results in blood pressure lowering. This effect has been demonstrated to be dose-dependent and directly correlated with felodipine plasma concentrations [4,5]. Moreover, an increase in the heart rate was observed directly after felodipine administration, which is likely caused by baroreflex-activated sympathetic mechanisms [5].

Felodipine is a BCS class II compound of high lipophilicity and low solubility [6]. Despite a nearly complete absorption of the drug, the oral bioavailability is only 15–20% due to the high first-pass metabolism [1,7]. Felodipine is listed by the FDA as a sensitive CYP3A4 substrate [8], and it is primarily metabolized by cytochrome P450 (CYP) 3A4 [9], with dehydrofelodipine as its main but pharmacologically inactive metabolite. As CYP3A4 is one of the major enzymes expressed in the gastrointestinal tract, intestinal metabolism after drug absorption strongly contributes to presystemic drug elimination, as observed for many other sensitive CYP3A4 substrates [10]. For felodipine, it is reported that more than 50% of the absorbed dose is metabolized in the gut wall [11]. Hence, the bioavailability and clearance of felodipine can be strongly affected by CYP3A4 perpetrators, resulting in significant changes in plasma concentrations and leading to an increased risk of adverse drug reactions, such as flush, headache, hypotension, tachycardia, or peripheral edema [1,12]. For example, a six-fold or two-fold increase in the felodipine area under the plasma concentration–time curve (AUC) could be observed after co-administration with the CYP3A4 inhibitors itraconazole or erythromycin [13,14]. Furthermore, the oral intake of felodipine with grapefruit juice, which acts as an intestinal CYP3A4 inactivator, increases its bioavailability from 15% to about 25%, resulting in a mean increase in the felodipine AUC of 72% [11]. In contrast, a 15-fold decrease in the AUC could be observed if felodipine was administered to patients treated with carbamazepine and phenytoin, inducers of CYP3A4, resulting in felodipine plasma concentrations below the therapeutic range [15]. Thus, the impact of CYP3A4-mediated drug–drug interactions (DDIs) on the pharmacokinetics and pharmacodynamics (PD) of felodipine should be throrougly investigated. For this purpose, physiologically based pharmacokinetic (PBPK) modeling is recognized as a valuable tool by the regulatory agencies FDA and EMA [16,17,18,19]. 

Therefore, this study aimed to develop a parent–metabolite PBPK/PD model of felodipine and its metabolite dehydrofelodipine that comprehensively describes (1) the pharmacokinetics of felodipine and its metabolite and (2) the effect of felodipine on the diastolic blood pressure and heart rate, and (3) to apply the model in DDI simulations with felodipine as a CYP3A4 victim drug and erythromycin, itraconazole, carbamazepine, and phenytoin as CYP3A4 perpetrators. The thoroughly evaluated model is publicly available in the Open Systems Pharmacology (OSP) repository on GitHub and can be applied to investigate and predict the effect of CYP3A4 perpetrators.

## 2. Materials and Methods

### 2.1. Software

Concentration–time profiles from published clinical studies were digitized with Engauge Digitizer Version 12.1 (M. Mitchell [20], 2020) according to the best practices proposed by Wojtyniak et al. [21]. The PBPK/PD model was developed with PK-Sim^®^ and MoBi^®^ (Open Systems Pharmacology Suite 9.1, released under the GNU General Public License version 2 (GPLv2) by the Open Systems Pharmacology community, www.open-systems-pharmacology.org, 2020). Parameter optimization (via Monte Carlo and Levenberg–Marquardt algorithms) and sensitivity analyses were performed within PK-Sim^®^. R 3.6.3 (The R Foundation for Statistical Computing, Vienna, Austria, 2019) was used for performance evaluations, non-compartmental analyses, and generation of plots.

### 2.2. Clinical Data

Plasma concentration−time profiles of felodipine and dehydrofelodipine as well as effect–time profiles of diastolic blood pressure and heart rate were gathered and digitized from published clinical studies. The collected studies provided plasma concentration–time and effect–time profiles (1) after intravenous and oral administration of felodipine in (2) single- and multiple-dosing regimens (3) over a broad dose range. Studies were split into a training dataset for model building and a test dataset for model evaluation. Concentration–time profiles for the training dataset were selected according to the following criteria: (1) coverage of intravenous and oral administration routes, (2) broad dosing range, (3) availability of both felodipine and dehydrofelodipine data, and (4) measurements in healthy participants without co-medication. Effect–time profiles for the PD model training dataset were selected from studies (1) covering a broad dosing range and (2) providing diastolic blood pressure and heart rate placebo measurements to analyze diurnal variations in baseline blood pressure and heart rate.

### 2.3. PBPK Model Building

To perform mathematical simulations of the collected clinical studies, virtual individuals were generated based on the mean and mode demographics reported in the respective study protocols. If no information was provided, a 30-year-old, male, European individual with body weight and height calculated based on the PK-Sim^®^ population database was used [22,23,24,25]. The relative expression of CYP3A4 in the different organs of the body was defined using the PK-Sim^®^ expression database [26]. Information on the selected expression profile and CYP3A4 reference concentration is provided in Table S18 of the Supplementary Materials.

Information on physicochemical parameters and absorption, distribution, metabolism, and excretion (ADME) processes were collected from the literature. Model input parameter values that could not be informed from the literature were estimated by mathematical optimization.

The felodipine parent–metabolite PBPK model was built in a stepwise procedure. First, an initial parent felodipine PBPK model was developed. Second, the initial model was complemented by the dehydrofelodipine metabolite model. Parameter values optimized for the initial parent PBPK model were then refined together with the parameter values of the metabolite PBPK model. As it can be assumed that small molecules like felodipine and its metabolite undergo passive glomerular filtration in the kidney, a glomerular filtration rate (GFR) fraction of 1 was used in the model. This parameter describes the fraction of drug that is passively filtered into the urine. Saturable metabolic processes were implemented via Michaelis–Menten kinetics. Otherwise, first-order clearance processes were used. The contribution of intestinal CYP3A4 metabolism after oral felodipine administration was predicted by calculating the intestinal fraction metabolized (f_m,int_) as the amount of felodipine metabolized in all intestinal compartments expressing CYP3A4 relative to the total amount of felodipine. The dissolution of tablet formulations with different felodipine release kinetics was described using Weibull dissolution functions implemented in PK-Sim^®^. The mathematical implementation of the Weibull dissolution is described in more detail in Section 1.1 of the Supplementary Materials. To evaluate the implemented dissolution parameters for the description of the extended-release formulation, the model was applied to predict in vivo dissolution profiles measured by Weitschies et al. [27] as an external model evaluation step.

### 2.4. PD Model Building

The PBPK model of felodipine was extended by a diastolic blood pressure and a heart rate PD model. As blood pressure and heart rate undergo fluctuations throughout the day, diurnal variation in the baseline diastolic blood pressure and heart rate was included based on models developed by Chae et al. [28] and Lott et al. [29]. The effect of felodipine on diastolic blood pressure and heart rate was described using a direct-effect *E_max_* model without lag time according to Equation (1).

(1) $$E=\frac{E_{max}\times C^{h}}{EC_{50}^{h}+C^{h}}$$

where *E_max_* is the maximum effect of felodipine on diastolic blood pressure or heart rate, *EC*_50_ is the concentration necessary to achieve half of *E_max_*, *h* is the hill coefficient, and *C* is the felodipine plasma concentration.

*E_max_* and *EC*_50_ values were optimized using the diastolic blood pressure and heart rate training dataset. A detailed description of the PD model building is provided in the Supplementary Materials Section 1.6.

### 2.5. Model Evaluation

The performance of the felodipine model was evaluated graphically by comparing (1) simulated plasma concentration−time profiles, as well as diastolic blood pressure and heart rate profiles, to the respective observed measurements; (2) all predicted plasma concentration, diastolic blood pressure, and heart rate values to their corresponding observed values in goodness-of-fit plots; and (3) predicted and observed AUC (calculated from the time of drug administration to the time of the last concentration measurement (AUC_last_)) and maximum plasma concentration (C_max_) values in goodness-of-fit plots. Additionally, the mean relative deviation (MRD) of predicted plasma concentrations, diastolic blood pressure, and heart rate values, as well as the geometric mean fold error (GMFE) of predicted AUC_last_ and C_max_ values, were calculated as quantitative measures, as described in Equations (S3) and (S4) in the Supplementary Materials.

### 2.6. DDI Modeling

The felodipine PBPK model was applied as a CYP3A4 victim drug model to predict DDIs with the CYP3A4 inhibitors erythromycin and itraconazole and the CYP3A4 inducers carbamazepine and phenytoin. For erythromycin [30], itraconazole [31], carbamazepine [32], and phenytoin (unpublished, in-house), we used available PBPK models that have been previously evaluated for DDI predictions as CYP3A4 perpetrator models. 

To establish DDI simulations, virtual individuals were generated and administration protocols of felodipine and the perpetrator drugs were established according to the information provided in the study protocols, which is summarized in Tables S10, S13 and S16 of the Supplementary Materials. In the itraconazole–felodipine study, an extended-release formulation of felodipine was administered that was not reflected in the felodipine PBPK model. To describe the dissolution kinetics of this formulation, the Weibull parameters were optimized based on felodipine control plasma concentration–time profiles. In the carbamazepine–phenytoin–felodipine DDI study, felodipine was administered to healthy individuals as a control and to epileptic patients receiving carbamazepine or phenytoin as a long-term anticonvulsant treatment. Based on information provided in the study protocol and dosing recommendations from the drug labels, a typical administration protocol was established to simulate carbamazepine and phenytoin administration. In the DDI simulation, felodipine administration began after reaching carbamazepine and phenytoin steady-state levels. The pharmacokinetics of the perpetrators were implemented using the published drug-dependent parameters of the PBPK models without any further adjustments.

The PBPK perpetrator models were coupled with the felodipine model using the CYP3A4 interaction parameters that were implemented and evaluated in these models. The mathematical implementation of (1) the mechanism-based CYP3A4 inhibition by erythromycin, (2) the competitive inhibition of CYP3A4 by itraconazole and its metabolites, and (3) CYP3A4 induction by carbamazepine and phenytoin is described in the Supplementary Materials Section 4. Furthermore, drug-dependent parameters of the perpetrator models are provided in the Supplementary Materials in the respective section.

The DDI performance of felodipine as a CYP3A4 victim drug was assessed by comparing predicted versus observed (1) felodipine and dehydrofelodipine plasma concentration−time profiles, as well as diastolic blood pressure and heart-rate effect–time profiles, with and without co-administration of the perpetrators and (2) DDI AUC_last_ ratios and DDI C_max_ ratios of felodipine and dehydrofelodipine in goodness-of-fit plots. Here, the limits proposed by Guest et al. [33] were used to evaluate the prediction success. Additionally, GMFEs of the predicted DDI AUC_last_ and C_max_ ratios were calculated.

## 3. Results

### 3.1. Pharmacokinetic Model

The parent−metabolite PBPK model of felodipine and dehydrofelodipine was built and evaluated using 49 clinical studies. Overall, these clinical studies provided 82 concentration–time profiles of felodipine for intravenous and oral administration, as summarized in Table 1. Additionally, 12 concentration–time profiles of the metabolite dehydrofelodipine were reported. A detailed overview of all clinical studies involved, including administration protocols, the demographics of the participants, and the assignment to the test or training dataset is provided in the Supplementary Materials Table S1.

**Figure 1:**
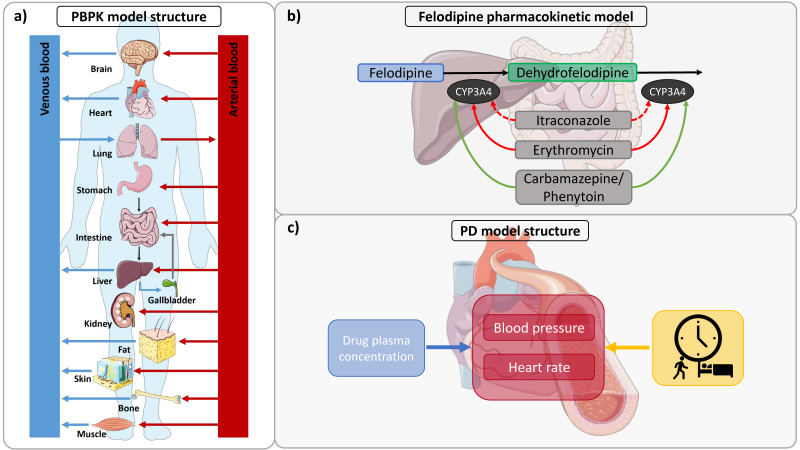
(**a**) Structure of a whole-body PBPK model. In this multi-compartmental modeling approach, compartments represent ADME-relevant organs of the body. The compartments are interconnected with arterial (red arrows) and venous (blue arrows) blood flows. (**b**) Metabolic pathways of felodipine and dehydrofelodipine and DDI network. Felodipine is metabolized by CYP3A4 to dehydrofelodipine, which is also metabolized by CYP3A4 (black arrows). Itraconazole and erythromycin are competitive (dotted red arrow) and mechanism-based (red arrow) inhibitors of CYP3A4, respectively, and inhibit the metabolism of felodipine and its metabolite, while carbamazepine and phenytoin induce CYP3A4. (**c**) Structure of the PBPK/PD model extension. Blood pressure and heart rate undergo diurnal variations (yellow). Alterations in diastolic blood pressure and heart rate are directly correlated to felodipine plasma concentrations (blue). Drawings by Servier, licensed under CC BY 3.0 [77]. CYP: cytochrome P450, DDI: drug–drug interaction, PBPK: physiologically based pharmacokinetic, PD: pharmacodynamics.

The compartmental structure of a whole-body PBPK model and the implemented metabolic pathways for felodipine and dehydrofelodipine are illustrated in Figure 1a,b, respectively. The final felodipine model accounts for (1) CYP3A4 metabolism to dehydrofelodipine and (2) passive glomerular filtration, and the dehydrofelodipine model accounts for (3) CYP3A4-mediated clearance, (4) unspecific hepatic clearance, and (5) passive glomerular filtration. To describe the biotransformation of felodipine to dehydrofelodipine via CYP3A4, the Michaelis–Menten constant (K_m_) was acquired from the literature, while the catalytic rate constant (k_cat_) was estimated. To adequately describe the plasma concentrations after the administration of the extended-release formulation, the formulation-specific felodipine solubility was estimated in addition to the Weibull parameters, which was approximately eight-fold lower compared to the felodipine solubility gathered from the literature and used in the model otherwise. Only sparse information on the metabolism of dehydrofelodipine was found in the literature; therefore, the implemented clearance processes had to be estimated. The final drug-dependent input parameters in comparison to the parameters available in the literature are listed in Table 2.

The predicted in vivo dissolution–time profiles after the administration of an extended-release felodipine tablet are displayed in Figure S4 of the Supplementary Materials. Here, the dissolution was well-predicted by the model, with a mean MRD of 1.51 for all dissolution measurements.

Furthermore, the model predicted the f_m,int_ as ~53% and an oral bioavailability of 13–18% after oral felodipine administration, implying a high extent of intestinal metabolism as well as a high first-pass metabolism.

When the model was applied to predict the pharmacokinetics in hypertensive individuals, the plasma concentrations were underpredicted. The adjustment of the CYP3A4 k_cat_ for each study could improve the model predictions considerably. Hence, the k_cat_ values were reduced on average by 32% (range: 20–43%), resulting in an overall lower felodipine clearance.

**Figure 2:**
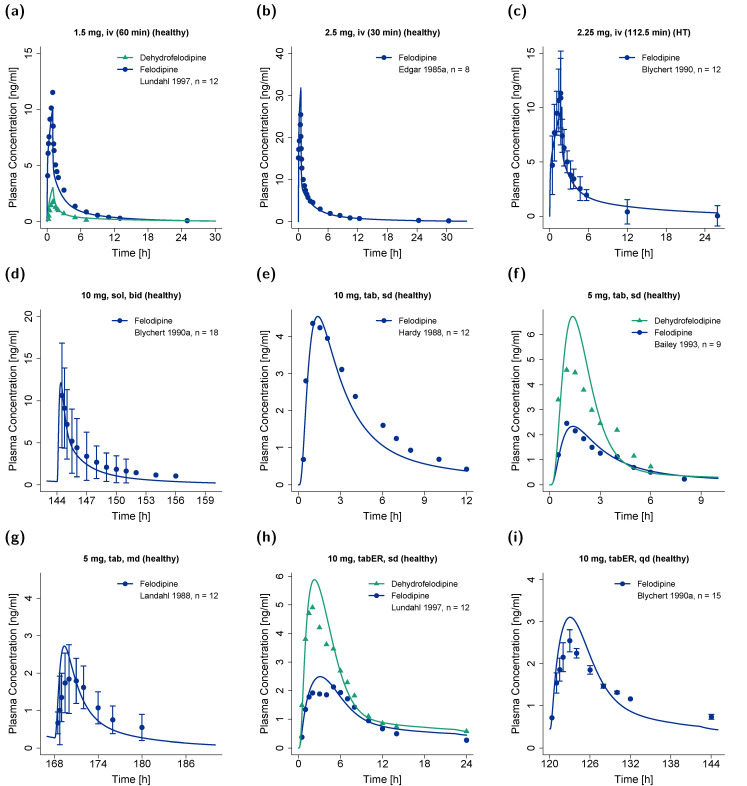
Predicted plasma concentration–time profiles of felodipine after administration as (**a**–**c**) intravenous infusion, (**d**) oral solution, (**e**–**g**) conventional tablet, or (**h**,**i**) extended-release tablet in comparison to observed data [11,35,45,47,48,51,71]. Observed data are shown as dots (felodipine) and triangles (dehydrofelodipine) ± standard deviation (if available); model predictions are shown as lines (blue: felodipine, green: dehydrofelodipine). bid: twice daily, HT: hypertensive, iv: intravenous, n: number of individuals, sd: single dose, sol: solution, tab: tablet, tabER: extended-release tablet.

**Figure 3:**
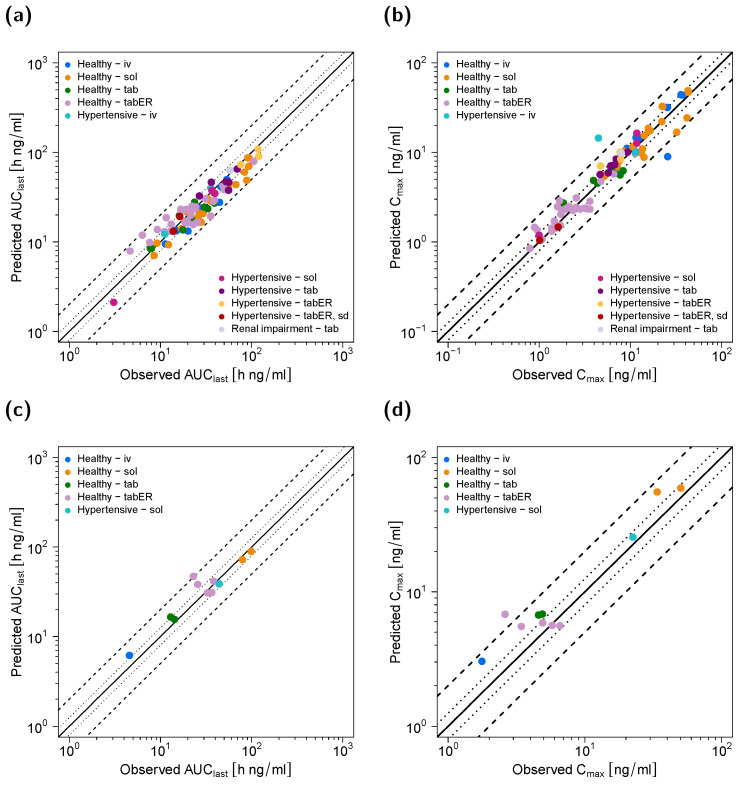
Performance of the felodipine PBPK model. Predicted compared to observed AUC_last_ and C_max_ values of (**a**,**b**) felodipine and (**c**,**d**) dehydrofelodipine stratified by route of administration and health status of the study participants. The line of identity is shown as a solid line; 1.25-fold deviation is shown as dotted lines; 2-fold deviation is shown as dashed lines. Study references are listed in Table 1. AUC_last_: area under the plasma concentration−time curve from the time of dosing to the time of last concentration measurement, C_max_: maximum plasma concentration, iv: intravenous, sol: solution, tab: tablet, tabER: extended-release tablet, sd: single dose.

Figure 2 shows selected plasma concentration−time profiles predicted by the model in comparison to observed clinical data, and Figure 3 shows goodness-of-fit plots comparing predicted versus observed AUC_last_ and C_max_ values of felodipine and dehydrofelodipine for all studies. A comprehensive evaluation of the felodipine parent–metabolite PBPK model is provided in the Supplementary Materials, including linear and semi-logarithmic plasma concentration–time profiles of all simulated studies (Figures S2 and S3); goodness-of-fit plots of predicted versus observed (1) AUC_last_, (2) C_max_, and (3) plasma concentration values (Figures S5 and S6); and the GMFE and MRD values of all studies (Tables S3 and S4). Overall, 89% and 82% of all felodipine and dehydrofelodipine predicted plasma concentrations, respectively, deviated less than two-fold from the observed values. The overall mean MRD of 1.67 and GMFE values for the AUC_laxt_ and C_max_ of 1.26 and 1.28, respectively, indicated a good model performance.

### 3.2. Pharmacodynamic Model

The felodipine parent–metabolite PBPK model was extended by a PD model, describing the effect of felodipine on the diastolic blood pressure and heart rate. Overall, 30 blood pressure–time profiles and 22 heart rate–time profiles from a total of 17 clinical studies were used to establish the PD model. The measurements were derived from healthy as well as hypertensive individuals.

Some parameters of the circadian models [28,29] were adjusted for each study individually, as described in detail in the Supplementary Materials Section 1.6. In summary, values for the circadian amplitudes (*amp*) were used as provided by the model authors, while the circadian phase and the mean diastolic blood pressure and heart rate (*BP_mean_, HR_mean_*) were optimized. After individually optimizing the diurnal model parameters for the studies of the training dataset using placebo blood pressure and heart rate profiles, parameter values of the *E_max_* model were estimated. The parameters used in the final PBPK/PD model are listed in Table 3.

**Figure 4:**
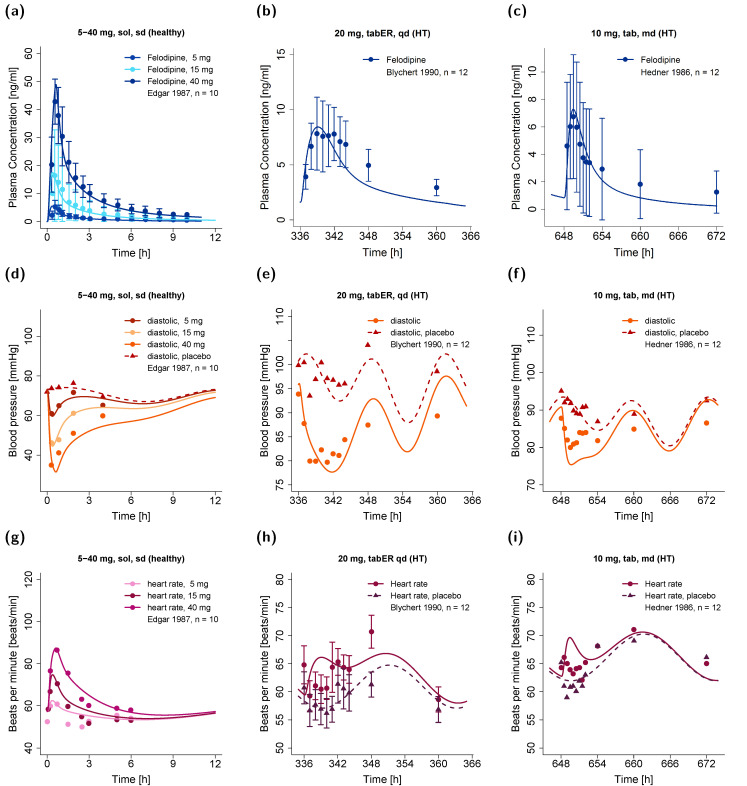
Predicted (**a**–**c**) plasma concentration–time profiles of felodipine with corresponding predicted (**d**–**f**) diastolic blood pressure (**g**–**i**) and heart rate effect–time profiles in healthy individuals (left panel) and hypertensive patients (center and right panels) in comparison to observed data [36,71,74]. Observed data are shown as dots and triangles ± standard deviation (if available); model predictions are shown as lines. HT: hypertensive, md: multiple dose, n: number of individuals, qd: once daily, sd: single dose, sol: solution, tab: tablet, tabER: extended-release tablet.

Selected effect–time profiles of diastolic blood pressure and heart rate predicted by the model in comparison to observed measurements are shown in Figure 4, along with corresponding felodipine plasma concentration–time profiles. Overall, the mean MRD values of 1.06 for both predicted diastolic blood pressure and heart rate measurements during felodipine administration indicated a good model performance. Predicted compared to observed effect–time profiles of diastolic blood pressure and heart rate for all studies are shown in Figures S9 and S10 in the Supplementary Materials. Goodness-of-fit plots of all predicted compared to observed diastolic blood pressure and heart rate measurements, as well as corresponding calculated MRD values, are shown in Figure S11 and Tables S7 and S8 in the Supplementary Materials.

### 3.3. DDI Modeling

The performance of the model for felodipine as a CYP3A4 victim drug in DDI simulations was assessed using one DDI study with erythromycin as the mechanism-based CYP3A4 inhibitor and one study with itraconazole (and its metabolites) as the competitive CYP3A4 inhibitor. Furthermore, one DDI study with carbamazepine and phenytoin as CYP3A4 inducers was used. In all studies, participants were pretreated with multiple doses of the perpetrator before felodipine was administered.

The setup of all DDI simulations is described in the Supplementary Materials Section 4.

**Figure 5:**
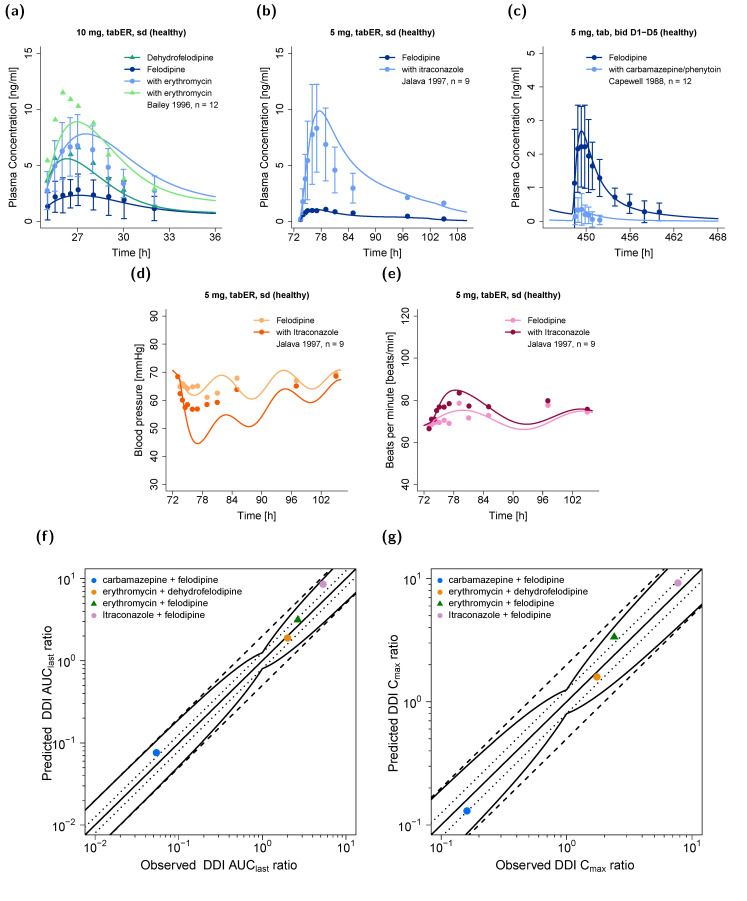
Upper row: predicted felodipine plasma concentration–time profiles with and without co-administration of the perpetrators (**a**) erythromycin, (**b**) itraconazole, and (**c**) carbamazepine and phenytoin in comparison to observed data [13,14,15]. Center row: predicted (**d**) diastolic blood pressure and (**e**) heart rate–time profiles with and without co-administration of itraconazole in comparison to observe data. Lower row: goodness-of-fit plots of predicted versus observed (**f**) DDI AUC_last_ ratios and (**g**) DDI C_max_ ratios. AUC_last_: area under the plasma concentration−time curve from the time of dosing to the time of last concentration measurement, bid: twice daily, C_max_: maximum plasma concentration, D: day, DDI: drug–drug interaction, n: number of individuals, sd: single dose, tab: tablet, tabER: extended-release tablet.

The DDI performance of the felodipine model is presented in Figure 5, showing (1) predicted compared to observed victim drug plasma concentration−time profiles, with and without the co-administration of the perpetrator drug; (2) predicted compared to observed diastolic blood pressure and heart rate effect–time profiles, with and without the co-administration of itraconazole; and (3) goodness-of-fit plots of predicted compared to observed DDI AUC_last_ and DDI C_max_ ratios. All predicted DDI AUC_last_ and DDI C_max_ ratios were within the limits proposed by Guest et al. [33], with mean GMFE values of 1.31 and 1.23, respectively.

## 4. Discussion

A whole-body parent−metabolite PBPK/PD model of felodipine and its main metabolite dehydrofelodipine was successfully established. The model was able to describe and predict plasma concentration−time profiles of felodipine and dehydrofelodipine as well as alterations in the diastolic blood pressure and heart rate after the intravenous administration (1–3 mg) of felodipine and oral administration (5–40 mg) as a solution, tablet, or extended-release tablet. The performance of the parent–metabolite PBPK/PD model was thoroughly evaluated, and the model was applied to predict DDI scenarios with felodipine as a CYP3A4 victim drug.

PBPK modeling of felodipine has previously been applied to investigate specific scenarios, such as the study of intestinal availability and metabolism [81,87,88] or the comparison of the pharmacokinetics after the administration of extended-release formulations from different manufacturing sites [89]. In contrast to prior work investigating the felodipine concentration–effect relationship [4,73,90], the presented model is the first parent–metabolite PBPK/PD model of felodipine. Moreover, the model is capable of investigating the effect of DDIs on both felodipine pharmacokinetics and pharmacodynamics. The model was developed by considering a broad range of clinical studies (n = 49, Table 1), with model development and evaluation being comprehensively documented and the model files freely available in the Open Systems Pharmacology repository (https://github.com/Open-Systems-Pharmacology, accessed on 12 July 2022).

Felodipine is extensively metabolized by CYP3A4, and the implementation of CYP3A4 as the sole route of felodipine metabolism was sufficient to describe its pharmacokinetics. It undergoes extensive metabolic degradation, and no unchanged felodipine is found in the urine, which is in line with the simulations obtained by the presented model, as only a very low fraction (<0.5%) of felodipine was predicted to be excreted unchanged in the urine. The dissolution of solid oral formulations was described by Weibull functions. For the extended-release formulation, the Lint80 dissolution model was tested as well, assuming the linear release of felodipine from the formulation until 80% of the administered dose was dissolved. Although a linear dissolution pattern was described in the literature [27], felodipine plasma concentration–time profiles could be more accurately described using a Weibull dissolution model with an estimated dissolution time (50% dissolved) of 173 min, which is in accordance with dissolution measurements from the literature [27]. However, to predict the plasma concentration–time profiles for extended-release tablets, a separate, formulation-specific felodipine solubility had to be estimated, as the application of the literature-derived felodipine solubility (7.15 mg/L) resulted in an overprediction of felodipine plasma concentrations after ~5–6 h hours. It was assumed that this overprediction resulted from an overestimation of the felodipine absorption after the transition of the tablet to distal intestinal compartments (e.g., caecum and colon ascendens). Here, the solubility was optimized as a surrogate to reduce felodipine absorption from these compartments. This resulted in an eight-fold lower solubility (compared to the literature value), which markedly improved the prediction of felodipine plasma concentrations. However, the fraction dissolved and fraction absorbed were not affected by the reduced solubility, and the model successfully described the dissolution–time profile of the extended-release tablet (Figure S4) [27].

Independent of the formulations, the model predicted the near-complete oral absorption of felodipine (fraction absorbed >95%), which was in accordance with the literature [1]. Moreover, the fraction of the administered dose metabolized by CYP3A4 in the intestines was predicted as approximately 53%, confirming the high extent of intestinal felodipine metabolism reported by Lundahl and coworkers [11]. The model also successfully described the observed low oral bioavailability of felodipine (13–18%) resulting from the extensive presystemic CYP3A4 metabolism [36]. In conclusion, intestinal absorption, metabolism, and bioavailability were well described, allowing the application of DDI simulations to investigate the influence of CYP3A4 perpetrators on felodipine pharmacokinetics. The overall good model performance could be quantified by the calculated mean GMFE values for the AUC_last_ and C_max_ of 1.26 and 1.28, respectively.

The felodipine parent–metabolite PBPK model was developed using plasma concentration–time profiles from healthy individuals and was subsequently applied to predict felodipine pharmacokinetics in hypertensive individuals, which resulted in an underprediction of the observed plasma concentration–time profiles. Higher felodipine exposure in hypertensive patients was also observed in the literature, along with a decrease in felodipine clearance by approximately two-fold, while absorption and distribution were similar compared to healthy individuals [51]. It was assumed that the observed differences in pharmacokinetics were mainly related to the older age of the hypertensive patients compared with healthy individuals and not to hypertension itself [51,91]. However, as the age of the individuals was considered during the establishment of the virtual simulations and was sufficient to describe the pharmacokinetics of felodipine in healthy middle-aged and elderly individuals (Figures S2 and S3(av,be,by)), an additional impact of hypertension-related factors may not fully be excluded. No information on the potential effects of hypertension on felodipine pharmacokinetics could be found in the literature. However, hypertension is a common risk factor for cardiovascular and renal disease [92], and inflammatory processes are involved in the genesis of hypertension and the progression of organ damage [92,93]. The downregulation of CYP enzymes, including CYP3A4, by inflammation has previously been described [94] and has also been observed in patients with, e.g., chronic kidney disease [95]. Hence, an immune-mediated downregulation of CYP3A4 in hypertensive individuals appears plausible. Therefore, CYP3A4 k_cat_ was separately optimized for healthy and hypertensive populations, yielding a mean reduction in CYP3A4 k_cat_ of 32% for hypertensive individuals and thus improving predictions in comparison to an unstratified CYP3A4 k_cat_ approach. However, clinical studies are needed to further investigate this hypothesis.

The PD model focused on the effect of felodipine on diastolic blood pressure and heart rate but not systolic blood pressure, due to a lack of data. Pronounced decreases in systolic blood pressure have been observed in hypertensives [71] but not in healthy individuals, even after high doses of up to 40 mg [5,54]. Thus, more systolic blood pressure measurements from healthy individuals would have been necessary to accurately describe the differences in PD effect magnitudes.

To describe the effect of felodipine on diastolic blood pressure and heart rate, the concentration–effect relationship was described using a direct-effect *E_max_* model without lag time, similar to other PD models of felodipine [4,73,90]. The concentration–effect relationship was established using felodipine plasma concentrations instead of heart concentrations, as felodipine shows a higher pharmacodynamic potency in vascular muscles compared to the myocardium [96]. An *EC*_50_ value of 40 nmol/L and an *E_max_* value of 56.18 mmHg were estimated for the diastolic blood pressure PD model. In contrast, other studies reported lower *EC*_50_ (~8 nmol/L) and *E_max_* (~29 mmHg) values to describe the plasma concentration–effect relationship [4,73]. However, our PBPK/PD analysis included blood pressure effect–time profiles for broader felodipine dosing, plasma concentration, and PD effect ranges, which unsurprisingly resulted in a higher estimated *E_max_* and related *EC*_50_ values.

Overall, the pharmacodynamic effect of felodipine was sufficiently described for healthy as well as hypertensive individuals, regardless of potential antihypertensive co-medication or initial baseline blood pressure. As only three studies provided heart rate data after multiple doses of felodipine, it was impossible to include the tolerance effects described in the literature [1] in the current model.

The PBPK/PD model was finally applied in DDI simulations, and the impact of CYP3A4 perpetrators on felodipine plasma concentrations was overall well described. For the DDI prediction with the CYP3A4 inhibitors erythromycin or itraconazole, C_max_ and t_max_ were slightly overpredicted. Nonetheless, the magnitude of the interaction was sufficiently explained by the model, illustrated by predicted versus observed DDI AUC_last_ and C_max_ ratios of 1.17 and 1.39 for the erythromycin–felodipine DDI and 1.60 and 1.19 for the itraconazole–felodipine DDI. The erythromycin–felodipine DDI study revealed the possible CYP3A4 metabolism of dehydrofelodipine [14]. Therefore, a CYP3A4-mediated clearance of dehydrofelodipine was implemented in addition to an unspecific hepatic clearance process. As no further information on dehydrofelodipine metabolism was available from the literature, the erythromycin–felodipine DDI was used in the training dataset to guide the estimation of the clearance parameters. Furthermore, the effect of the CYP3A4 inducers carbamazepine and phenytoin on felodipine pharmacokinetics was well described, although the carbamazepine–phenytoin-felodipine DDI study was conducted in patients undergoing long-term anticonvulsant treatment and did not provide detailed information on their treatment regimen. A decrease in felodipine bioavailability from 15% to around 1% was reported if felodipine was administered to patients on anticonvulsant therapy, while the model predicted a decrease in bioavailability from around 13% to 2% [15]. Here, DDI AUC_last_ and C_max_ ratios of 1.40 and 0.81 were calculated. Overall, the DDI AUC_last_ and DDI C_max_ effect ratios were within the limits proposed by Guest et al. [33], with mean GMFE values of 1.31 and 1.23, respectively.

The itraconazole–felodipine DDI study additionally provided pharmacodynamic measurements of diastolic blood pressure and heart rate. The model was capable of describing the observed increase in heart rate if felodipine was co-administered with itraconazole; however, the observed decrease in blood pressure was slightly overpredicted. Previous studies showed that itraconazole may increase blood pressure by inhibiting 11β-hydroxysteroid dehydrogenase type 2 [97], and various case reports in the literature have described elevated blood pressure during itraconazole treatment. However, increased blood pressure was mainly associated with high itraconazole doses (>400 mg/day) [98], and controlled studies investigating the potential effects of itraconazole on blood pressure are lacking. Based on the available data, the potential effects of itraconazole on blood pressure could not be determined for the itraconazole–felodipine DDI study, where itraconazole was administered in low doses (200 mg/day) over a short time period (4 days).

Overall, the presented analysis demonstrated that felodipine is susceptible to CYP3A4-mediated DDIs. Case reports describing the occurrence of major side effects, such as edema or tachycardia, if itraconazole or erythromycin was administered to felodipine-treated patients emphasize the clinical relevance of these interactions [12,99]. The parent–metabolite PBPK model of felodipine can be applied in DDI simulations to predict the effect of CYP3A4 perpetrators on bioavailability and plasma concentrations, which may help to support the design of dedicated clinical DDI studies. As the model also describes the effects on heart rate and diastolic blood pressure, it may also be applied to guide treatment decisions or optimizations.

## 5. Conclusions

A felodipine parent–metabolite PBPK/PD model was successfully developed to describe the pharmacokinetics of felodipine after intravenous and oral administrations over a broad dosing range, along with the effect of felodipine on diastolic blood pressure and heart rate. The pharmacokinetics of felodipine, especially its metabolism via CYP3A4, were sufficiently described, as shown by the adequate description of the oral felodipine bioavailability and the successful prediction of DDIs with CYP3A4 inhibitors and inducers. The felodipine PBPK model can be applied in DDI predictions as a CYP3A4 victim drug to evaluate the effects of CYP3A4 perpetrators, e.g., as a probe model to investigate grapefruit–drug interactions. Thereby, the PBPK model can be used to estimate the contribution of intestinal metabolism to overall bioavailability.

## Figures and Tables

**Table 1:** Clinical studies used for the development of the felodipine PBPK/PD model.

Route	Dose (mg)	n (PK)	n (DBP)	n (HR)	Reference
**Healthy Individuals**					
iv	1–3	9	1	1	[5,11,34,35,36,37,38]
sol, sd	5–40	15	8	8	[5,7,34,35,36,39,40,41,42,43,44]
sol, md	10	1	0	0	[45]
tab, sd	5–10	4	1	2	[7,46,47,48,49,50]
tab, md	5–10	4	0	0	[45,51,52]
tabER, sd	5–40	24	7	6	[7,11,13,14,53,54,55,56,57,58,59,60,61,62,63,64,65,66,67,68,69]
tabER, md	5–10	6	1	1	[45,57,68,70]
**Profiles (healthy)**		**63**	**18**	**18**	
**Hypertensive Individuals**					
iv	1–2.25	2	1	1	[71,72]
sol, sd	0.83–10	3	2	0	[34,72]
tab, sd	10	2	2	0	[73]
tab, md	5–10	5	5	1	[51,73,74,75]
tabER, sd	20	3	1	1	[71,76]
tabER, md	20	4	1	1	[71,75,76]
**Profiles (hypertensive)**		**19**	**12**	**4**	
**Profiles (total)**		**82**	**30**	**22**	

DBP: diastolic blood pressure, HR: heart rate, iv: intravenous, md: multiple dose, n: number of profiles, PK: pharmacokinetics, sd: single dose, sol: solution, tab: tablet, tabER: extended-release tablet.

**Table 2:** Drug-dependent parameters of the felodipine PBPK model.

Parameter	Unit	Model	Literature	Reference	Description
**Felodipine**					
MW	g/mol	384.25	384.25	[78]	Molecular weight
fu, plasma	%	0.36	0.36	[79]	fraction unbound in plasma
Solubility (pH)	mg/L	7.15 (6.5)	1.2 (7) 7.15 (6.5) 14.3 (7.1) 19.7 (7)	[80,81,82]	Solubility at reference pH
Solubility-tabER (pH)	mg/L	0.89 (7)	-	-	Solubility at reference pH used for extended-release tablets
logP	-	4.36	3.44 3.80 4.36 4.46 4.64	[78,81,82,83]	Lipophilicity
Intestinal permeability	cm/min	2.76 × 10^−4^	4.42 × 10^−4^ 3.06 × 10^−4^ 2.64 × 10^−4^	[81,84]	Transcellular intestinal permeability
GFR fraction	-	1	-	-	Fraction of filtered drug in the urine
K_m_-CYP3A4	µmol/L	2.81	0.94 2.81 26.4	[9,81,85]	CYP3A4 Michaelis–Menten constant
k_cat_-CYP3A4	1/min	250.44	-	-	CYP3A4 catalytic rate constant
Weibull time-tab	min	54.86	-	-	Tablet dissolution profile shape
Weibull shape-tab	-	1.32	-	-	Tablet dissolution time (50% dissolved)
Weibull time-tabER	min	173.04	-	-	Extended-release tablet dissolution profile shape
Weibull shape-tabER	-	1.30	-	-	Extended-release tablet dissolution time (50% dissolved)
Partition coefficient	-	Diverse	RR		Cell to plasma partition coefficients
Cellular permeability	cm/min	0.42	PK-Sim		Permeability into the cellular space
**Dehydrofelodipine**					
MW	g/mol	382.24	382.24	[86]	Molecular weight
pKa (base)	-	4.06	4.06	[86]	Acid dissociation constant
fu, plasma	%	0.68	-	-	fraction unbound in plasma
Solubility (pH)	mg/L	2.93	2.93	[86]	Solubility at reference pH
logP	-	3.32	4.24	[86]	Lipophilicity
Intestinal permeability	cm/min	1.38 × 10^−4^	estimated via PK-Sim^®^		Transcellular intestinal permeability
GFR fraction	-	1	-	-	Fraction of filtered drug in the urine
CL-CYP3A4	1/min	35.74	-	-	CYP3A4-mediated clearance
CL-hepatic	1/min	2.76	-	-	Unspecific hepatic clearance
Partition coefficient	-	Diverse	S		Cell to plasma partition coefficients
Cellular permeability	cm/min	0.04	CDS		Permeability into the cellular space

CYP3A4: cytochrome P450 3A4, GFR: glomerular filtration rate, PK-Sim: PK-Sim standard, RR: Rodgers and Rowland, S: Schmitt, CDS: charge-dependent Schmitt, tab: tablet, tabER: extended-release tablet.

**Table 3:** Parameters used for the diastolic blood pressure and heart rate PD model.

Parameter	Unit	Model	Literature	Reference	Description
**Diastolic Blood Pressure Model**					
*E_max_*	mmHg	56.18	-	-	Maximum effect on diastolic blood pressure
*EC*_50_	µmol/L	0.04	-	-	Concentration for half-maximal effect
amp_24_	%	2.14	2.14	[28]	Amplitude for 24 h period
amp_12_	%	5.93	5.93	[28]	Amplitude for 12 h period
phase_24_	h	5.28 (2.10) ^a^	-	-	Phase for 24 h period
phase_12_	h	0.16 (2.10) ^a^	-	-	Phase for 12 h period
BP_mean_	mmHg	70.4 (6.76) ^a^; 91.9 (6.60) ^a,b^	69.5	[28]	Mean diastolic blood pressure over 24 h
**Heart Rate Model**					
*E_max_*	bpm	39.71	-	-	Maximum effect on heart rate
EC_50_	µmol/L	0.05	-	-	Concentration for half-maximal effect
h	-	1.40	-	-	Hill coefficient
*amp*	%	6.3	6.3	[29]	Amplitude
phase	h	11.8 (5.13) ^a^	9.2	[29]	Phase
*HR_mean_*	bpm	62.2 (4.21) ^a^; 66.4 (3.32) ^a,b^	66.2	[29]	Mean heart rate over 24 h

^a^ mean (standard deviation), individually optimized values for individual simulations in Supplementary Materials Table S5. ^b^ mean diastolic blood pressure and heart rate values used for hypertensive individuals.

## Data Availability

All modeling files, including utilized clinical study data, can be found at: https://github.com/Open-Systems-Pharmacology.

## Supplementary Materials

The following are available online at https://www.mdpi.com/article/10.3390/pharmaceutics14071474/s1: Comprehensive reference manual, providing documentation of the complete model performance assessment.

## References

[1] AstraZeneca LP. Plendil (Felodipine). Extended-Release Tablets. https://www.accessdata.fda.gov/drugsatfda_docs/label/2012/019834s025lbl.pdf.

[2] Heumann Pharma Felodipin Retard Heumann. https://www.heumann.de/fileadmin/user_upload/produkte/infos/Fachinformation-Felodipin-retard-Heumann.pdf.

[3] Williams B., Mancia G., Spiering W., Agabiti Rosei E., Azizi M., Burnier M., Clement D.L., Coca A., de Simone G., Dominiczak A. (2018). 2018 ESC/ESH Guidelines for the management of arterial hypertension. Eur. Heart J..

[4] Blychert E., Edgar B., Elmfeldt D., Hedner T. (1992). Plasma concentration—Effect relationships for felodipine: A meta analysis. Clin. Pharmacol. Ther..

[5] Bengtsson-Hasselgren B., Edgar B., Rönn O. (1988). Dose-dependent effects of felodipine on diuresis and natriuresis in healthy subjects. J. Cardiovasc. Pharmacol..

[6] Benet L.Z., Broccatelli F., Oprea T.I. (2011). BDDCS applied to over 900 drugs. AAPS J..

[7] Edgar B., Lundborg P., Regårdh C.G. (1987). Clinical pharmacokinetics of felodipine. A summary. Drugs.

[8] U.S. Food and Drug Administration Drug Development and Drug Interactions. Table of Substrates, Inhibitors and Inducers. https://www.fda.gov/drugs/drug-interactions-labeling/drug-development-and-drug-interactions-table-substrates-inhibitors-and-inducers.

[9] Walsky R.L., Obach R.S. (2004). Validated assays for human cytochrome P450 activities. Drug Metab. Dispos..

[10] Fritz A., Busch D., Lapczuk J., Ostrowski M., Drozdzik M., Oswald S. (2019). Expression of clinically relevant drug-metabolizing enzymes along the human intestine and their correlation to drug transporters and nuclear receptors: An intra-subject analysis. Basic Clin. Pharmacol. Toxicol..

[11] Lundahl J., Regårdh C.G., Edgar B., Johnsson G. (1997). Effects of grapefruit juice ingestion-pharmacokinetics and haemodynamics of intravenously and orally administered felodipine in healthy men. Eur. J. Clin. Pharmacol..

[12] Neuvonen P.J., Suhonen R. (1995). Itraconazole interacts with felodipine. J. Am. Acad. Dermatol..

[13] Jalava K.M., Olkkola K.T., Neuvonen P.J. (1997). Itraconazole greatly increases plasma concentrations and effects of felodipine. Clin. Pharmacol. Ther..

[14] Bailey D.G., Bend J.R., Arnold J.M., Tran L.T., Spence J.D. (1996). Erythromycin-felodipine interaction: Magnitude, mechanism, and comparison with grapefruit juice. Clin. Pharmacol. Ther..

[15] Capewell S., Freestone S., Critchley J.A.J.H., Pottage A., Prescott L.F. (1988). Reduced felodipine bioavailability in patients taking anticonvulsants. Lancet.

[16] European Medicines Agency Guideline on the Investigation of Drug Interactions. https://www.ema.europa.eu/en/documents/scientific-guideline/guideline-investigation-drug-interactions-revision-1_en.pdf.

[17] European Medicines Agency Guideline on the Reporting of Physiologically Based Pharmacokinetic (PBPK) Modelling and Simulation. https://www.ema.europa.eu/en/documents/scientific-guideline/guideline-reporting-physiologically-based-pharmacokinetic-pbpk-modelling-simulation_en.pdf.

[18] Zhang X., Yang Y., Grimstein M., Fan J., Grillo J.A., Huang S.-M., Zhu H., Wang Y. (2020). Application of PBPK Modeling and Simulation for Regulatory Decision Making and Its Impact on US Prescribing Information: An Update on the 2018-2019 Submissions to the US FDA’s Office of Clinical Pharmacology. J. Clin. Pharmacol..

[19] U.S. Food and Drug Administration Physiologically Based Pharmacokinetic Analyses—Format and Content. https://www.fda.gov/media/101469/download.

[20] Mitchell M., Muftakhidinov B., Winchen T. Engauge Digitizer Software. http://markummitchell.github.io/engauge-digitizer.

[21] Wojtyniak J., Britz H., Selzer D., Schwab M., Lehr T. (2020). Data Digitizing: Accurate and Precise Data Extraction for Quantitative Systems Pharmacology and Physiologically-Based Pharmacokinetic Modeling. CPT Pharmacomet. Syst. Pharmacol..

[22] National Center for Health Statistics Hyattsville MD 20782 Third National Health and Nutrition Examination Survey, (NHANES III). http://www.cdc.gov/nchs/nhanes.htm.

[23] Valentin J. (2002). Basic anatomical and physiological data for use in radiological protection: Reference values. A report of age- and gender-related differences in the anatomical and physiological characteristics of reference individuals. ICRP Publication 89. Ann. ICRP.

[24] Tanaka G., Kawamura H. (1996). Anatomical and Physiological Characteristics for Asian Reference Man: Male and Female of Different Ages: Tanaka Model.

[25] Schlender J.F., Meyer M., Thelen K., Krauss M., Willmann S., Eissing T., Jaehde U. (2016). Development of a Whole-Body Physiologically Based Pharmacokinetic Approach to Assess the Pharmacokinetics of Drugs in Elderly Individuals. Clin. Pharm..

[26] Open Systems Pharmacology Suite Community PK-Sim® Ontogeny Database Documentation, Version 7.3. https://github.com/Open-Systems-Pharmacology/OSPSuite.Documentation/blob/master/PK-SimOntogenyDatabaseVersion7.3.pdf.

[27] Weitschies W., Wedemeyer R.-S., Kosch O., Fach K., Nagel S., Söderlind E., Trahms L., Abrahamsson B., Mönnikes H. (2005). Impact of the intragastric location of extended release tablets on food interactions. J. Control. Release.

[28] Chae D., Kim Y., Park K. (2019). Characterization of circadian blood pressure patterns using non-linear mixed effects modeling. Transl Clin. Pharmacol..

[29] Lott D., Lehr T., Dingemanse J., Krause A. (2018). Modeling Tolerance Development for the Effect on Heart Rate of the Selective S1P1 Receptor Modulator Ponesimod. Clin. Pharmacol. Ther..

[30] Frechen S., Dallmann A., Solodenko J. Building and Evaluation of a PBPK Model for Erythromycin in Healthy Adults. https://github.com/Open-Systems-Pharmacology/OSP-PBPK-Model-Library/blob/master/Erythromycin/Erythromycin_evaluation_report.md.

[31] Hanke N., Frechen S., Moj D., Britz H., Eissing T., Wendl T., Lehr T. (2018). PBPK Models for CYP3A4 and P-gp DDI prediction: A modeling network of rifampicin, itraconazole, clarithromycin, midazolam, alfentanil, and digoxin. CPT Pharmacomet. Syst. Pharmacol..

[32] Fuhr L.M., Marok F.Z., Hanke N., Selzer D., Lehr T. (2021). Pharmacokinetics of the CYP3A4 and CYP2B6 Inducer Carbamazepine and Its Drug-Drug Interaction Potential: A Physiologically Based Pharmacokinetic Modeling Approach. Pharmaceutics.

[33] Guest E.J., Aarons L., Houston J.B., Rostami-Hodjegan A., Galetin A. (2011). Critique of the two-fold measure of prediction success for ratios: Application for the assessment of drug-drug interactions. Drug Metab. Dispos..

[34] Edgar B., Bengtsson B., Elmfeldt D., Lundborg P., Nyberg G., Raner S., Rönn O. (1985). Acute diuretic/natriuretic properties of felodipine in man. Drugs.

[35] Edgar B., Regårdh C.G., Johnsson G., Johansson L., Lundborg P., Löfberg I., Rönn O. (1985). Felodipine kinetics in healthy men. Clin. Pharmacol. Ther..

[36] Edgar B., Regårdh C.G., Lundborg P., Romare S., Nyberg G., Rönn O. (1987). Pharmacokinetic and pharmacodynamic studies of felodipine in healthy subjects after various single, oral and intravenous doses. Biopharm. Drug Dispos..

[37] Sluiter H.E., Huysmans F.T., Thien T.A., Koene R.A.P. (1985). Haemodynamic effects of intravenous felodipine in normotensive and hypertensive subjects. Drugs.

[38] Sutfin T.A., Lind T., Gabrielsson M., Regårdh C.G. (1990). Biliary secretion of felodipine metabolites in man after intravenous [14C]felodipine. Eur. J. Clin. Pharmacol..

[39] Soons P.A., Mulders T.M.T., Uchida E., Schoemaker H.C., Cohen A.F., Breimer D.D. (1993). Stereoselective pharmacokinetics of oral felodipine and nitrendipine in healthy subjects: Correlation with nifedipine pharmacokinetics. Eur. J. Clin. Pharmacol..

[40] Abrahamsson B., Johansson D., Torstensson A., Wingstrand K. (1994). Evaluation of solubilizers in the drug release testing of hydrophilic matrix extended-release tablets of felodipine. Pharm. Res..

[41] Bengtsson-Hasselgren B., Rönn O., Blychert L.O., Edgar B., Raner S. (1990). Acute effects of felodipine and nifedipine on hepatic and forearm blood flow in healthy men. Eur. J. Clin. Pharmacol..

[42] Johnsson G., Murray G., Tweddel A., Hutton I. (1983). Haemodynamic effects of a new vasodilator drug, felodipine, in healthy subjects. Eur. J. Clin. Pharmacol..

[43] Wingstrand K., Abrahamsson B., Edgar B. (1990). Bioavailability from felodipine extended-release tablets with different dissolution properties. Int. J. Pharm..

[44] Soons P.A., Roosemalen M.C.M., Breimer D.D. (1990). Enantioselective determination of felodipine and other chiral dihydropyridine calcium entry blockers in human plasma. J. Chromatogr..

[45] Blychert E., Wingstrand K., Edgar B., Lidman K. (1990). Plasma concentration profiles and antihypertensive effect of conventional and extended-release felodipine tablets. Br. J. Clin. Pharmacol.

[46] Guo L.-Q., Chen Q.-Y., Wang X., Liu Y.-X., Chu X.-M., Cao X.-M., Li J.-H., Yamazoe Y. (2007). Different roles of pummelo furanocoumarin and cytochrome P450 3A5*3 polymorphism in the fate and action of felodipine. Curr. Drug Metab..

[47] Hardy B.G., Bartle W.R., Myers M., Bailey D.G., Edgar B. (1988). Effect of indomethacin on the pharmacokinetics and pharmacodynamics of felodipine. Br. J. Clin. Pharmacol..

[48] Bailey D.G., Arnold J.M., Munoz C., Spence J.D. (1993). Grapefruit juice-felodipine interaction: Mechanism, predictability, and effect of naringin. Clin. Pharmacol. Ther..

[49] Edgar B., Bailey D., Bergstrand R., Johnsson G., Regårdh C.G. (1992). Acute effects of drinking grapefruit juice on the pharmacokinetics and dynamics of felodipine-and its potential clinical relevance. Eur. J. Clin. Pharmacol..

[50] Sakamoto T., Ohtake Y., Itoh M., Tabata S., Kuriki T., Uno K. (1993). Determination of felodipine enantiomers using chiral stationary phase liquid chromatography and gas chromatography/mass spectrometry, and the study of their pharmacokinetic profiles in human and dog. Biomed. Chromatogr..

[51] Landahl S., Edgar B., Gabrielsson M., Larsson M., Lernfelt B., Lundborg P., Regårdh C.G. (1988). Pharmacokinetics and blood pressure effects of felodipine in elderly hypertensive patients. A comparison with young healthy subjects. Clin. Pharm..

[52] Smith S.R., Wilkins M.R., Jack D.B., Kendall M.J., Laugher S. (1987). Pharmacokinetic interactions between felodipine and metoprolol. Eur. J. Clin. Pharmacol..

[53] Goosen T.C., Cillié D., Bailey D.G., Yu C., He K., Hollenberg P.F., Woster P.M., Cohen L., Williams J.A., Rheeders M. (2004). Bergamottin contribution to the grapefruit juice-felodipine interaction and disposition in humans. Clin. Pharmacol. Ther..

[54] Hasselgren B., Rönn O., Edgar B., Johansson P., Wall B. (1990). Pharmacokinetics and hemodynamic and diuretic/natriuretic effects of felodipine administered as an extended-release tablet. Cardiovasc. Drugs Ther..

[55] Lown K.S., Bailey D.G., Fontana R.J., Janardan S.K., Adair C.H., Fortlage L.A., Brown M.B., Guo W., Watkins P.B. (1997). Grapefruit juice increases felodipine oral availability in humans by decreasing intestinal CYP3A protein expression. J. Clin. Investig..

[56] Lundahl J., Regårdh C.G., Edgar B., Johnsson G. (1995). Relationship between time of intake of grapefruit juice and its effect on pharmacokinetics and pharmacodynamics of felodipine in healthy subjects. Eur. J. Clin. Pharmacol..

[57] Lundahl J.U.E., Regårdh C.G., Edgar B., Johnsson G. (1998). The interaction effect of grapefruit juice is maximal after the first glass. Eur. J. Clin. Pharmacol..

[58] Patel Devang S., Shanker N., Shah Sweety K., Thakkar Vaishali K., Mehta Nirali N., Srivstava Ambrish K., Singh S., Patel Chitrang G. (2011). Bioequivalence study of two oral extended release formulations of felodipine 10 mg tablets in healthy volunteers under fed condition. Pharma Sci. Monit. Int. J. Pharm. Sci..

[59] Pop A., Vlase L. (2008). Leucuta Pharmacokinetic study of felodipine after single oral dose of slow release formulations in healthy volunteers. Farmacia.

[60] Xiang Q., Li C., Zhao X., Cui Y.M. (2017). The influence of CYP3A5*3 and BCRPC421A genetic polymorphisms on the pharmacokinetics of felodipine in healthy Chinese volunteers. J. Clin. Pharm. Ther..

[61] Bailey D.G., Kreeft J.H., Munoz C., Freeman D.J., Bend J.R. (1998). Grapefruit juice-felodipine interaction: Effect of naringin and 6’,7’-dihydroxybergamottin in humans. Clin. Pharmacol. Ther..

[62] Gelal A., Balkan D., Ozzeybek D., Kaplan Y.C., Gurler S., Guven H., Benowitz N.L. (2005). Effect of menthol on the pharmacokinetics and pharmacodynamics of felodipine in healthy subjects. Eur. J. Clin. Pharmacol..

[63] Madsen J.K., Jensen J.D., Jensen L.W., Pedersen E.B. (1996). Pharmacokinetic interaction between cyclosporine and the dihydropyridine calcium antagonist felodipine. Eur. J. Clin. Pharmacol..

[64] Aguilar-Carrasco J.C., Carrasco-Portugal M.d.C., Flores-Murrieta F.J., Canizales-Quinteros S. (2015). Oral pharmacokinetics of felodipine in mexican healthy volunteers: Evidence for interethnic differences. Int. J. Pharmacol..

[65] Bailey D.G., Arnold J.M., Bend J.R., Tran L.T., Spence J.D. (1995). Grapefruit juice-felodipine interaction: Reproducibility and characterization with the extended release drug formulation. Br. J. Clin. Pharmacol..

[66] Bailey D.G., Dresser G.K., Kreeft J.H., Munoz C., Freeman D.J., Bend J.R. (2000). Grapefruit-felodipine interaction: Effect of unprocessed fruit and probable active ingredients. Clin. Pharmacol. Ther..

[67] Bailey D.G., Dresser G.K., Bend J.R. (2003). Bergamottin, lime juice, and red wine as inhibitors of cytochrome P450 3A4 activity: Comparison with grapefruit juice. Clin. Pharmacol. Ther..

[68] Dresser G.K., Bailey D.G., Carruthers S.G. (2000). Grapefruit juice-felodipine interaction in the elderly. Clin. Pharmacol. Ther..

[69] Dresser G.K., Urquhart B.L., Proniuk J., Tieu A., Freeman D.J., Arnold J.M., Bailey D.G. (2017). Coffee inhibition of CYP3A4 in vitro was not translated to a grapefruit-like pharmacokinetic interaction clinically. Pharmacol. Res. Perspect..

[70] A Comparative Study on the Relative Bioavailability of 2.5 and 5mg ER Tablets of Felodipine. 19834. https://www.accessdata.fda.gov/drugsatfda_docs/nda/pre96/19834-S002_PLENDILTABLETS_BIOEQR.PDF.

[71] Blychert E., Hedner T., Dahlöf C., Elmfeldt D. (1990). Plasma concentration-effect relationships of intravenous and extended-release oral felodipine in hypertensive patients. J. Cardiovasc. Pharmacol..

[72] Edgar B., Regårdh C.G., Attman P.O., Aurell M., Herlitz H., Johnsson G. (1989). Pharmacokinetics of felodipine in patients with impaired renal function. Br. J. Clin. Pharmacol..

[73] Larsson R., Karlberg B.E., Gelin A., Aberg J., Regårdh C.-G. (1990). Acute and steady-state pharmacokinetics and antihypertensive effects of felodipine in patients with normal and impaired renal function. J. Clin. Pharmacol..

[74] Hedner T., Samuelsson O., Sjögren E., Elmfeldt D. (1986). Treatment of essential hypertension with felodipine in combination with a diuretic. Eur. J. Clin. Pharmacol..

[75] Hedner T., Elmfeldt D., Dahlöf C., Sjögren E. (1987). Comparison of antihypertensive effect and pharmacokinetics of conventional and extended release felodipine tablets in patients with arterial hypertension. Drugs.

[76] Leenen F.H.H., Coletta E. (2010). Pharmacokinetic and antihypertensive profile of amlodipine and felodipine-ER in younger versus older patients with hypertension. J. Cardiovasc. Pharmacol..

[77] Les Laboratoires Servier Servier Medical Art. https://smart.servier.com/.

[78] Human Metabolome Database Metabocard for Felodipine (HMDB0015158). https://hmdb.ca/metabolites/HMDB0015158.

[79] Regårdh C.G., Edgar B., Olsson R., Kendall M., Collste P., Shansky C. (1989). Pharmacokinetics of felodipine in patients with liver disease. Eur. J. Clin. Pharmacol..

[80] Felle K., Persson B., Vessman J. (1984). Dissolution test for felodipine tablets using chemical oxidation in situ to maintain ‘sink conditions’. J. Pharm Biomed. Anal..

[81] Takano J., Maeda K., Bolger M.B., Sugiyama Y. (2016). The Prediction of the Relative Importance of CYP3A/P-glycoprotein to the Nonlinear Intestinal Absorption of Drugs by Advanced Compartmental Absorption and Transit Model. Drug Metab. Dispos..

[82] DRUGBANK Online-Felodipine. https://go.drugbank.com/drugs/DB01023.

[83] Van der Lee R., Pfaffendorf M., Koopmans R.P., van Lieshout J.J., van Montfrans G.A., van Zwieten P.A. (2001). Comparison of the time courses and potencies of the vasodilator effects of nifedipine and felodipine in the human forearm. Blood Press.

[84] Berben P., Brouwers J., Augustijns P. (2018). Assessment of Passive Intestinal Permeability Using an Artificial Membrane Insert System. J. Pharm. Sci..

[85] Galetin A., Clarke S.E., Houston J.B. (2002). Quinidine and haloperidol as modifiers of CYP3A4 activity: Multisite kinetic model approach. Drug Metab. Dispos..

[86] Chemaxon Chemicalize-Dehydrofelodipine. https://chemicalize.com.

[87] Gertz M., Houston J.B., Galetin A. (2011). Physiologically based pharmacokinetic modeling of intestinal first-pass metabolism of CYP3A substrates with high intestinal extraction. Drug Metab. Dispos..

[88] Heikkinen A.T., Baneyx G., Caruso A., Parrott N. (2012). Application of PBPK modeling to predict human intestinal metabolism of CYP3A substrates-An evaluation and case study using GastroPlus^TM^. Eur. J. Pharm. Sci..

[89] Jamei M., Abrahamsson B., Brown J., Bevernage J., Bolger M.B., Heimbach T., Karlsson E., Kotzagiorgis E., Lindahl A., McAllister M. (2020). Current status and future opportunities for incorporation of dissolution data in PBPK modeling for pharmaceutical development and regulatory applications: OrBiTo consortium commentary. Eur. J. Pharm. Biopharm..

[90] Wade J.R., Sambol N.C. (1995). Felodipine population dose-response and concentration-response relationships in patients with essential hypertension. Clin. Pharmacol. Ther..

[91] Blychert E., Edgar B., Elmfeldt D., Hedner T. (1991). A population study of the pharmacokinetics of felodipine. Br. J. Clin. Pharmacol..

[92] Oparil S., Acelajado M.C., Bakris G.L., Berlowitz D.R., Cífková R., Dominiczak A.F., Grassi G., Jordan J., Poulter N.R., Rodgers A. (2018). Hypertension. Nat. Rev. Dis. Prim..

[93] Muller D.N., Kvakan H., Luft F.C. (2011). Immune-related effects in hypertension and target-organ damage. Curr. Opin. Nephrol. Hypertens..

[94] De Jong L.M., Jiskoot W., Swen J.J., Manson M.L. (2020). Distinct Effects of Inflammation on Cytochrome P450 Regulation and Drug Metabolism: Lessons from Experimental Models and a Potential Role for Pharmacogenetics. Genes.

[95] Zhao P., Vieira M.D.L.T., Grillo J.A., Song P., Wu T.-C., Zheng J.H., Arya V., Berglund E.G., Atkinson A.J., Sugiyama Y. (2012). Evaluation of Exposure Change of Nonrenally Eliminated Drugs in Patients With Chronic Kidney Disease Using Physiologically Based Pharmacokinetic Modeling and Simulation. J. Clin. Pharmacol.

[96] Elmfeldt D., Hedner T. (1985). Antihypertensive effects of felodipine compared with placebo. Drugs.

[97] Beck K.R., Thompson G.R., Odermatt A. (2020). Drug-induced endocrine blood pressure elevation. Pharmacol Res..

[98] Hoffmann W.J., McHardy I., Thompson G.R. (2018). Itraconazole induced hypertension and hypokalemia: Mechanistic evaluation. Mycoses.

[99] Liedholm H., Nordin G. (1991). Erythromycin-Felodipine Interaction. DICP.

