# Supplementary material for: A Physiologically Based Pharmacokinetic and Pharmacodynamic Model of the CYP3A4 Substrate Felodipine for Drug–Drug Interaction Modeling
Source: Pharmaceutics. 2022 Jul 15;14(7):1474. doi: 10.3390/pharmaceutics14071474 (PMC9322514; doi:10.3390/pharmaceutics14071474)
Supplement: Supplementary file 1 [file pharmaceutics-14-01474-s001.zip › pharmaceutics-1794012-supplementary.pdf]

pharmaceutics

A physiologically-based pharmacokinetic and pharmacodynamic model of the CYP3A4 substrate felodipine for drug-drug interaction modeling

Supplementary Materials

Laura Maria Fuhr[1], Fatima Zahra Marok[1], Maximilian Mees[1], Felix Mahfoud[2,3], Dominik Selzer[1], Thorsten Lehr[1]

[1]Clinical Pharmacy, Saarland University, Saarbrücken, Germany
[2]Department of Internal Medicine III, Cardiology, Angiology, Intensive Care Medicine, Saarland University Medical Center and Saarland University Faculty of Medicine, 66421 Homburg, Germany
[3]Institute for Medical Engineering and Science, Massachusetts Institute of Technology, Cambridge, MA 02139, USA

Funding
This research was funded by the German Federal Ministry of Education and Research (BMBF), grant number 031L0161C (“OSMOSES”).

Conflict of Interest
Thorsten Lehr has received research grants from the German Federal Ministry of Education and Research (grant 031L0161C). Felix Mahfoud is supported by Deutsche Gesellschaft für Kardiologie (DGK), Deutsche Forschungsgemeinschaft (SFB TRR219), and Deutsche Herzstiftung. He has received scientific support from Medronic and ReCor Medical and speaker honoraria from Astra-Zeneca, Bayer, Boehringer Ingelheim, Inari, Medtronic, Merck, and ReCor Medical. Laura Maria Fuhr, Fatima Zahra Marok, Dominik Selzer and Maximilian Mees declare no conflict of interest.

Corresponding Author
Thorsten Lehr, PhD
Clinical Pharmacy, Saarland University
Campus C2 2
66123 Saarbrücken, Germany
ORCID: 0000 0002 8372 1465
Phone: +49 681 302 70255
Email: thorsten.lehr@mx.uni-saarland.de

Contents

1 Physiologically based pharmacokinetic/pharmacodynamic (PBPK/PD) modeling 4

1.1 Felodipine formulations . . . 4
1.2 PBPK model evaluation . . . 4
1.3 Drug-drug interaction (DDI) model evaluation . . . 4
1.4 Sensitivity analysis . . . 5
1.5 Mathematical implementation of drug-drug interactions (DDIs) . . . 6
1.6 Pharmacodynamic (PD) model building . . . 8

2 Felodipine 10

2.1 PBPK model building . . . 10
2.2 Felodipine clinical studies . . . 11
2.3 Felodipine drug-dependent parameters . . . 15
2.4 Plasma concentration-time profiles . . . 16
2.4.1 Linear . . . 16
2.4.2 Semi-logarithmic . . . 25
2.4.3 Dissolution-time profile . . . 34
2.5 Model evaluation . . . 35
2.5.1 Plasma concentration goodness-of-fit plots . . . 35
2.5.2 Mean relative deviation of predicted plasma concentrations . . . 36
2.5.3 AUC_{last} and C_{max} goodness-of-fit plots . . . 39
2.5.4 Geometric mean fold error of predicted AUC_{last} and C_{max} values . . . 40
2.5.5 Sensitivity analysis . . . 44

3 Felodipine - pharmacodynamic modeling 47

3.1 PD model building . . . 47
3.2 PD model parameters . . . 48
3.3 Effect-time profiles . . . 51
3.3.1 Diastolic blood pressure . . . 51
3.3.2 Heart rate . . . 55
3.4 Model evaluation . . . 58
3.4.1 Goodness-of-fit plots . . . 58
3.4.2 Mean relative deviation of predicted effect measurements . . . 59

4 Felodipine DDIs 61

4.1 DDI modeling . . . 61
4.2 Erythromycin-felodipine DDI . . . 62
4.2.1 Erythromycin drug-dependent parameters . . . 63
4.2.2 Erythromycin-felodipine clinical studies . . . 64
4.2.3 Plasma concentration-time profiles . . . 65
4.2.4 DDI AUC_{last} and C_{max} ratio goodness-of-fit plots . . . 66
4.2.5 Geometric mean fold error of predicted DDI AUC_{last} and C_{max} ratios . . . 67
4.3 Itraconazole-felodipine DDI . . . 68
4.3.1 Itraconazole drug-dependent parameters . . . 69
4.3.2 Itraconazole-felodipine clinical studies . . . 71
4.3.3 Plasma concentration-time and effect-time profiles . . . 72
4.3.4 DDI AUC_{last} and C_{max} ratio goodness-of-fit plots . . . 74
4.3.5 Geometric mean fold error of predicted DDI AUC_{last} and C_{max} ratios . . . 75
4.4 Carbamazepine-felodipine DDI . . . 76
4.4.1 Carbamazepine drug-dependent parameters . . . 77

4.4.2 Carbamazepine-phenytoin-felodipine clinical studies 79
4.4.3 Plasma concentration-time profiles 80
4.4.4 DDI AUC_{last} and C_{max} ratio goodness-of-fit plots 81
4.4.5 Geometric mean fold error of predicted DDI AUC_{last} and C_{max} ratios . . . 82

5 System-dependent parameters **83**

Abbreviations **86**

1 Physiologically based pharmacokinetic/pharmacodynamic (PBPK/PD) modeling

1.1 Felodipine formulations

The dissolution of oral felodipine formulations was described using the Weibull function according to Equations S1 and S2:

$$m = 1 - exp(\frac{-(t - T_{lag}^{\beta})}{\alpha}) \tag{S1}$$

$$\alpha = (T_d)^{\beta} \tag{S2}$$

where m = fraction of dissolved drug at time t, T_{lag} = lag time before the onset of dissolution, α = scale parameter, β = shape parameter, T_d = time needed to dissolve 63% of the formulation

1.2 PBPK model evaluation

As quantitative performance measures, the mean relative deviation (MRD) of predicted plasma concentrations as well as blood pressure and heart rate measurements and the geometric mean fold error (GMFE) of predicted area under the plasma concentration-time curve values calculated from the time of drug administration to the time of the last concentration measurement (AUC_{last}) and maximum plasma concentration (C_{max}) values were calculated according to Equation S3 and Equation S4, respectively. Values ≤ 2 are considered as adequate model performance metrics.

$$MRD = 10^x, \text{ with } x = \sqrt{\frac{1}{m}\sum_{i=1}^{m}(\log_{10} c_{pred,i} - \log_{10} c_{obs,i})^2} \tag{S3}$$

where $c_{pred,i}$ = predicted plasma concentration, $c_{obs,i}$ = corresponding observed plasma concentration and m = number of observed values.

$$GMFE = 10^x, \text{ with } x = \frac{1}{n}\sum_{i=1}^{n}|\log_{10}(\frac{PK_{pred,i}}{PK_{obs,i}})| \tag{S4}$$

where $PK_{pred,i}$ = predicted AUC_{last} or C_{max} value, $PK_{obs,i}$ = corresponding observed AUC_{last} or C_{max} value and n = the number of studies.

1.3 Drug-drug interaction (DDI) model evaluation

Modeled DDIs were evaluated by comparison of predicted versus observed plasma concentration-time profiles of felodipine with and without co-administration of perpetrator drugs. Furthermore, predicted DDI AUC_{last} ratios (Equation S5) and DDI C_{max} ratios (Equation S6) were calculated and compared to observed values.

$$\text{DDI } AUC_{last} \text{ ratio} = \frac{AUC_{last} \text{ victim drug during DDI}}{AUC_{last} \text{ victim drug control}} \tag{S5}$$

$$\text{DDI } C_{max} \text{ ratio} = \frac{C_{max} \text{ victim drug during DDI}}{C_{max} \text{ victim drug control}} \quad \text{(S6)}$$

Additionally, GMFE values of the predicted DDI AUC_{last} ratios and DDI C_{max} ratios were calculated according to Equation S4.

1.4 Sensitivity analysis

Sensitivity of the final models to single parameter values (local sensitivity analysis) was calculated as relative change of the area under the plasma concentration-time curve (AUC). Sensitivity analysis was carried out using a relative perturbation of 1000% (variation range 10.0, maximum number of 9 steps). Parameters were included into the analysis if they were optimized, if they are associated with optimized parameters or if they might have a strong impact due to calculation methods used in the model.

The sensitivity to a parameter value was calculated as the ratio of the relative change of the simulated AUC to the relative variation of the parameter around its value used in the final model according to Equation S7.

$$S = \frac{\Delta AUC}{AUC} \cdot \frac{p}{\Delta p} \quad \text{(S7)}$$

where S = sensitivity of the AUC to the examined model parameter, ΔAUC = change of the simulated AUC, AUC = simulated AUC with the original parameter value, Δp = change of the examined parameter value, p = original parameter value. A sensitivity of +1.0 signifies that a 10% increase of the examined parameter value causes a 10% increase of the simulated AUC.

1.5 Mathematical implementation of drug-drug interactions (DDIs)

Competitive inhibition

Competitive inhibitors reversibly bind to the active site of an enzyme or transporter and compete with the substrate for binding. The maximum reaction velocity (v_{max}) remains unaffected, while the Michaelis-Menten constant (K_m) is increased ($K_{m,app}$, Equation S8) in the presence of the inhibitor. The reaction velocity (v) during administration of a competitive inhibitor is described by Equation S9 [1]:

$$K_{m,app} = K_m \cdot \left(1 + \frac{[I]}{K_i}\right) \tag{S8}$$

$$v = \frac{v_{max} \cdot [S]}{K_{m,app} + [S]} \tag{S9}$$

where $K_{m,app}$ = Michaelis-Menten constant in the presence of the inhibitor, K_m = Michaelis-Menten constant, $[I]$ = free inhibitor concentration, K_i = dissociation constant of the inhibitor-enzyme complex, v = reaction velocity, v_{max} = maximum reaction velocity, $[S]$ = free substrate concentration.

Mechanism-based inactivation

Mechanism-based inactivation is an irreversible type of inhibition. Baseline enzyme activity will be regained after clearance of the inactivator and de novo synthesis of the enzyme (time-dependency). The enzyme degradation rate constant (k_{deg}) is increased ($k_{deg,app}$, Equation S10), while its synthesis rate (R_{syn}) remains unaffected. The enzyme turnover during administration of a mechanism-based inactivator is described by Equation S11. As mechanism-based inactivators are also competitive inhibitors, the K_m in the Michaelis-Menten reaction velocity equation is substituted by $K_{m,app}$ as shown in Equation S12 [1]:

$$k_{deg,app} = k_{deg} + \left(\frac{k_{inact} \cdot [I]}{K_I + [I]}\right) \tag{S10}$$

$$\frac{dE(t)}{dt} = R_{syn} - k_{deg,app} \cdot E(t) \tag{S11}$$

$$v = \frac{v_{max} \cdot [S]}{K_{m,app} + [S]} = \frac{k_{cat} \cdot E(t) \cdot [S]}{K_{m,app} + [S]} \tag{S12}$$

where $k_{deg,app}$ = enzyme degradation rate constant in the presence of the mechanism-based inactivator, k_{deg} = enzyme degradation rate constant, k_{inact} = maximum inactivation rate constant, $[I]$ = free inactivator concentration, K_I = concentration for half-maximal inactivation, $E(t)$ = enzyme concentration, R_{syn} = enzyme synthesis rate, v = reaction velocity, v_{max} = maximum reaction velocity, $[S]$ = free substrate concentration, $K_{m,app}$ = Michaelis-Menten constant in the presence of inactivator, k_{cat} = catalytic rate constant.

Induction

Induction of enzymes is mediated by the activation of nuclear receptors, increasing gene expression. The baseline activity of the enzyme is regained after clearance of the inducer and degradation of the enzyme (time-dependency). The enzyme synthesis rate (R_{syn}) is increased ($R_{syn,app}$, Equation S13), while its k_{deg} remains unaffected. The enzyme turnover during administration of an inducer is described by Equation S14 [1], the reaction velocity is described by Equation S15:

$$R_{syn,app} = R_{syn} \cdot \left(1 + \frac{E_{max} \cdot [Ind]}{EC_{50} + [Ind]}\right) \tag{S13}$$

$$\frac{dE(t)}{dt} = R_{syn,app} - k_{deg} \cdot E(t) \tag{S14}$$

$$v = \frac{v_{max} \cdot [S]}{K_m + [S]} = \frac{k_{cat} \cdot E(t) \cdot [S]}{K_m + [S]} \tag{S15}$$

where $R_{syn,app}$ = enzyme synthesis rate in the presence of inducer, R_{syn} = enzyme synthesis rate, E_{max} = maximal induction effect in vivo, $[Ind]$ = free inducer concentration, EC_{50} = concentration for half-maximal induction in vivo, $E(t)$ = enzyme concentration, k_{deg} = enzyme degradation rate constant, v = reaction velocity, v_{max} = maximum reaction velocity, $[S]$ = free substrate concentration, K_m = Michaelis-Menten constant, k_{cat} = catalytic rate constant.

1.6 Pharmacodynamic (PD) model building

The PBPK model of felodipine was extended by a diastolic blood pressure and heart rate PD model.

As blood pressure and heart rate undergo fluctuations throughout the day, a circadian rhythm was implemented for blood pressure and heart rate according to models by Chae et al. [2] and Lott et al. [3], respectively. Circadian variability of blood pressure is described according to Equations S16 and S17.

$$circ_{BP}(t) = amp_{24,BP} \cdot cos(\frac{2\pi}{24} \cdot (t - phase_{24,BP})) + amp_{12,BP} \cdot cos(\frac{2\pi}{12} * (t - phase_{12,BP})) \quad \text{(S16)}$$

$$BP(t) = BP_{mean} \cdot (1 + circ_{BP}(t)) \quad \text{(S17)}$$

where $circ_{BP}$ = circadian rythm of blood pressure at t, t = time, $amp_{24,BP}$ / $amp_{12,BP}$ = amplitudes, for period 24 h and 12 h, respectively, $phase_{24,BP}$ and $phase_{12,BP}$ = shift over time for period 24 h and 12 h, respectively, BP_{mean} = mean blood pressure over 24 h.

Similarly, circadian variability of heart rate is described according to Equations S18 and S19.

$$circ_{HR}(t) = amp_{HR} \cdot cos(\frac{2\pi}{24} \cdot (t - phase_{HR})) \quad \text{(S18)}$$

$$HR(t) = HR_{mean} \cdot (1 + circ_{HR}(t)) \quad \text{(S19)}$$

where $circ_{HR}$ = circadian rhythm of heart rate at t, t = time, amp_{HR} = amplitude, $phase_{HR}$ = shift over time, HR_{mean} = mean heart rate over 24h.

The effect was described using a direct maximum effect (E_{max}) model without lag time according to Equation S20.

$$E(t) = \frac{E_{max} \cdot C(t)^h}{EC_{50}^h + C(t)^h} \quad \text{(S20)}$$

where E = effect, t = time, E_{max} = maximum effect of felodipine on heart rate or blood pressure, EC_{50} = concentration necessary to achieve half of E_{max}, h = hill coefficient and C = felodipine plasma concentration.

Subsequently, blood pressure and heart rate are defined according to Equations S21 and S22, respectively.

$$BP(t) = BP_{mean} \cdot (1 + circ_{BP}(t)) - E(t) \quad \text{(S21)}$$

$$HR(t) = HR_{mean} \cdot (1 + circ_{HR}(t)) + E(t) \quad \text{(S22)}$$

The circadian rhythm of blood pressure and heart rate was optimized for each study individually. The circadian amplitudes ($amp_{24,BP}$, $amp_{12,BP}$, amp_{HR}) were adopted from Chae et al. [2] and Lott et al. [3], while circadian phases ($phase_{24,BP}$, $phase_{12,BP}$, $phase_{HR}$) and mean heart rate and blood pressure values (HR_{mean}, BP_{mean}) were optimized using the parameter identification tool implemented in MoBi. The interval between $phase_{24,BP}$ and $phase_{12,BP}$ identified by Chae et al. [2] was maintained. If clinical studies provided placebo profiles, the circadian parameters were optimized based on the placebo profiles and subsequently transferred to the

corresponding effect-time profiles measured during felodipine administration. If no placebo profiles were available, the parameters were carefully optimized based on the effect-time profiles during felodipine administration. For studies that displayed changes from baseline blood pressure instead of absolute blood pressure data in the effect-time profiles, absolute values were calculated based on the indicated baseline value before felodipine administration (BP_0). The parameters of the E_{max} model (EC_{50} and E_{max}) were optimized based on the blood pressure and heart rate training datasets, preserving the previously optimized parameters of the circadian model.

2 Felodipine

2.1 PBPK model building

All clinical studies used for model building, including information on participant demographics, the study protocol and assignment to training and test dataset, are listed in Table S1.

Felodipine has a chiral center at C4 [4], and is administered as racemic mixture. The two enantiomers are enantioselectively metabolized by cytochrome P450 (CYP) 3A4 to dehydrofelodipine, resulting in 2-fold higher maximum plasma concentrations of (S)-felodipine [4]. The stereoselective pharmacokinetics of the enantiomers could be described assuming different K_m values, without changing other drug-dependent parameters. Estimated K_m values are in accordance with literature, where approximately 2-fold higher K_m values were reported for (S)-felodipine [5]. Due to the small size of studies investigating the pharmacokinetics of (R)- and (S)-felodipine, the ability of the model to predict the stereoselective pharmacokinetics could no be thoroughly evaluated. All processes implemented in the final model are illustrated in Figure S1 and drug-dependent parameters used in the final model in comparison to parameters obtained from literature are listed in Table S2.

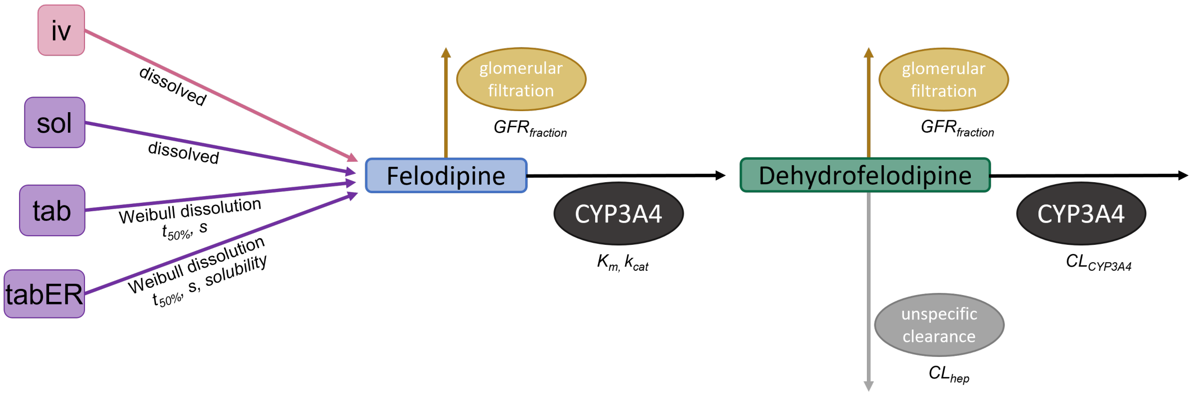

Figure S1: Implemented metabolic pathways of felodipine and dehydrofelodipine. The model describes intravenous and oral administration of felodipine. Dissolution of solid oral formulations (conventional and extended release tablets) is described using Weibull functions. Felodipine is metabolized by CYP3A4 to dehydrofelodipine and eliminated by passive glomerular filtration. Dehydrofelodipine undergoes CYP3A4 mediated clearance as well as unspecific hepatic clearance and is also eliminated by passive glomerular filtration. CL_{CYP3A4}: CYP3A4 mediated clearance, CL_{hep}: unspecific hepatic clearance, CYP3A4: cytochrome P450 3A4, $GFR_{fraction}$: Fraction of filtered drug in the urine, iv: intravenous, K_m: Michaelis-Menten constant, k_{cat}: catalytic rate constant, s: dissolution shape, sol: solution, $t_{50\%}$: dissolution time (50% dissolved), tab: tablet, tabER: extended release tablet.

Predicted compared to observed plasma concentration-time profiles of felodipine and dehydrofelodipine are displayed in Figures S2 (linear) and S3 (semi-logarithmic). Goodness-of-fit plots for plasma concentrations are presented in Figure S5, corresponding MRD values are listed in Table S3. The correlation of predicted and observed AUC_{last} and C_{max} values is presented in Figure S6. Corresponding values and overall GMFE values of all studies are listed in Table S4.

2.2 Felodipine clinical studies

Table S1: Clinical studies used for the development of the felodipine PBPK/PD model

Dose [mg]	Route	n	Women [%]	Age[a] [y]	Weight[a] [kg]	Height[a] [cm]	DHF	HR	BP	Dataset	Reference
**Healthy individuals**											
1	iv (20 min)	10	0	26 (22-33)	73 (67-82)		no	no	no	training	Bengtsson 1988 [6]
1	iv (20 min)	10	0	26 (22-33)	73 (66-82)	181 (171-188)	no	no	no	test	Edgar 1987 [7]
1	iv (30 min)	4	0	28 (23-36)	72 (69-73)		no	no	no	test	Sutfin 1990 [8]
1.5	iv (150 min)	10	0	32 (26-40)	73 ± 7.3	180 ± 5	no	yes	yes	test	Sluiter 1985 [9]
1.5	iv (60 min)	12	0	25 ±2	75 ±6		yes	no	no	training	Lundahl 1997 [10]
2.5	iv (30 min)	10	0	25 ± 1.3 (23-31)	74 ± 2 (66-82)		no	no	no	test	Edgar 1985 [11]
2.5	iv (30 min)	8	0	25 (22-31)	74 (69-82)		no	no	no	training	Edgar 1985a [12]
3	iv (20 min)	10	0	26 (22-33)	73 (67-82)		no	no	no	training	Bengtsson 1988 [6]
3	iv (20 min)	10	0	26 (22-33)	73 (66-82)	181 (171-188)	no	no	no	test	Edgar 1987 [7]
5	po (sol), sd	10	0	26 (22-33)	73 (67-82)		no	yes	yes	training	Bengtsson 1988 [6]
5	po (sol), sd	10	0	26 (22-33)	73 (66-82)	181 (171-188)	no	yes	yes	test	Edgar 1987 [7]
10	po (sol), sd	16	0				no	no	no	test	Abrahamsson 1994 [13]
10	po (sol), sd	9	0	28 ± 5	77 ± 4.5		no	yes	yes	test	Bengtsson 1990 [14]
10	po (sol), sd	12	0				no	no	no	test	Edgar 1987a [15]
10	po (sol), sd	16	0	24 (20-35)	72 (64-81)		no	no	no	training	Wingstrand 1990 [16]
10	po (sol), bid	18	0	(20-34)			no	no	no	test	Blychert 1990a [17]
10.35	po (sol), 0, 35 min	8	0	(22-31)			no	yes	yes	test	Johnsson 1983 [18]
15	po (sol), sd	10	0	26 (22-33)	73 (67-82)		no	yes	yes	test	Bengtsson 1988 [6]
15	po (sol), sd	10	0	26 (22-33)	73 (66-82)	181 (171-188)	no	yes	yes	test	Edgar 1987 [7]
20	po (sol), sd	12	0	25 ± 5 (20-34)	71 ± 7 (52-81)		yes	no	no	test	Soons 1993 [19]
20	po (sol), sd	4	0				yes	no	no	training	Soons 1990 [4]
27.5	po (sol), sd	10	0	25 ± 1.3 (23-31)	74 ± 2 (66-82)		no	no	no	test	Edgar 1985 [11]
27.5	po (sol), sd	8	0	25 (22-31)	75 (69-82)		no	no	no	test	Edgar 1985a [12]

assumed

[a]: mean ± standard deviation (range)

[c]: Patients were usually receiving other hypertensive medication, including beta blockers and diuretics

[d]: Doses between 0.5 - 1.5 mg were administered

[e]: 0.02 mg/min, for 105-120 min

[f]: Patients with renal impairment

° tablet brand: Plendil, * tablet brand: Hydac

- : no data available, bid: twice daily, BP: blood pressure; D: day; DHF: dehydrofelodipine, HR: heart rate, iv: intravenous, n: number of individuals, po: oral, qd: once daily, sd: single dose, sol: solution, tab: tablet, tabER: extended release tablet

Table S1: Clinical studies used for the development of the felodipine PBPK/PD model *(continued)*

Dose [mg]	Route	n	Women [%]	Age[a] [y]	Weight[a] [kg]	Height[a] [cm]	DHF	HR	BP	Dataset	Reference
40	po (sol), sd	10	0	26 (22-33)	73 (67-82)		no	yes	yes	training	Bengtsson 1988 [6]
40	po (sol), sd	10	0	26 (22-33)	73 (66-82)	181 (171-188)	no	yes	yes	test	Edgar 1987 [7]
5	po (*tab*), sd	9	0	(19-40)			yes	no	no	test	Bailey 1993 [20]
5	po (*tab*), sd	9	0	44 ± 5 (40-53)	77 ± 6.8 (66-85)		yes	no	no	training	Edgar 1992 [21]
5	po (*tab*), bid	12	0	26 (20-34)	75 (69-85)		no	no	no	test	Landahl 1988 [22]
5	po (*tab*), bid D1-D5	12	8	29 ± 8.9	72 ± 9.0		no	no	no	test	Capewell 1988 [23]
10	po (tab), sd	12	0				no	no	no	test	Edgar 1987a [15]
10	po (tab), sd	12	0	(20-27)	(51-78)	(168-183)	no	yes	yes	test	Guo 2007 [24]
10	po (tab), sd	1					no	no	no	test	Lindmark 2002 [25]
10	po (tab), sd	12	0	(22-39)	(69-92)		no	yes	yes	training	Hardy 1988 [26]
10	po (tab), bid	18	0	(20-34)			no	no	no	training	Blychert 1990a [17]
10	po (tab), qd	15	0	(18-38)			no	no	no	test	Blychert 1990a [17]
2.5, 5	po,tabER D1-D2: 2.5 mg qd; D3-8: 5 mg qd	6	67	(71-77)			no	yes	yes	test	Dresser 2000 [27]
5	po (tabER*), sd	9	44	24 (22-26)	60 (51-73)		no	yes	yes	test	Jalava 1997 [28]
5	po (tabER°), sd	30	47	26 ± 1.4	64 ± 1.2	166 ± 1.24	no	no	no	test	Aguilar-Carrasco 2015 [29]
5	po (tabER°), sd	12	58	(70-83)			no	yes	yes	training	Dresser 2000 [27]
5	po (tabER°), sd	12	0	(21-24)			no	no	no	test	Goosen 2004 [30]
5	po (tabER), sd	45	0	33 ± 2.8 (28-39)	63 ± 5.6 (50-77)		no	no	no	test	Xiang 2017 [31]
2x 2.5	po (tabER°), qd	20	0	(20-32)			no	no	no	test	Bioequivalence 1994 [32]
5	po (tabER°), qd	20	0	(20-32)			no	no	no	training	Bioequivalence 1994 [32]
10	po (tabER), sd	12	0	(18-45)			no	no	no	test	Bailey 1995 [33]
10	po (tabER), sd	12	0	29 ± 4.9	79 ± 9.0		yes	yes	yes	test	Madsen1996 [34]
10	po (tabER°), sd	12	0	(18-45)			yes	no	no	training	Bailey 1996 [35]
10	po (tabER°), sd	12	0	(18-45)			yes	no	no	test	Bailey 1998 [36]
10	po (tabER°), sd	12	17	28			no	no	no	test	Bailey 2000 [37]

assumed

[a]: mean ± standard deviation (range)

[c]: Patients were usually receiving other hypertensive medication, including beta blockers and diuretics

[d]: Doses between 0.5 - 1.5 mg were administered

[e]: 0.02 mg/min, for 105-120 min

[f]: Patients with renal impairment

° tablet brand: Plendil, * tablet brand: Hydac

- : no data available, bid: twice daily, BP: blood pressure; D: day; DHF: dehydrofelodipine, HR: heart rate, iv: intravenous, n: number of individuals, po: oral, qd: once daily, sd: single dose, sol: solution, tab: tablet, tabER: extended release tablet

Table S1: Clinical studies used for the development of the felodipine PBPK/PD model *(continued)*

Dose [mg]	Route	n	Women [%]	Age[a] [y]	Weight[a] [kg]	Height[a] [cm]	DHF	HR	BP	Dataset	Reference
10	po (tabER°), sd	6	25	(23-45)	64 (1.2)		no	no	no	test	Bailey 2003 [38]
10	po (tabER°), sd	5	40	46 (31-56)			no	no	no	test	Dresser 2017 [39]
10	po (tabER°), sd	12	0				no	no	no	test	Edgar 1987a [15]
10	po (tabER°), sd	12	0	27 (23-30)	76 (68-86)		no	yes	yes	test	Hasselgren 1990 [40]
10	po (tabER°), sd	10	0	(18-65)			no	no	no	test	Lown 1997 [41]
10	po (tabER°), sd	9	0	(23-33)	(69-86)		yes	yes	yes	test	Lundahl 1995 [42]
10	po (tabER°), sd	12	0	25 ±2	75 ±6		yes	no	no	test	Lundahl 1997 [10]
10	po (tabER°), sd	12	0	26 ±1	76 ±6		no	no	no	training	Lundahl 1998 [43]
10	po (tabER°), sd	24					no	no	no	test	Pop 2008 [44]
10	po (tabER°), sd	10	90	26 ± 5.3 (21-36)	57 ± 8.4 (52-80)	169 ± 8.8 (160-190)	yes	yes	yes	test	Gelal 2005 [45]
10	po (tabER), sd	6	50	(21-31)	(53-90)		no	no	no	test	Weitschies 2005 [46]
10	po (tabER°, fed), sd	56	0	29 ± 5.9 (18.42)	61 ± 6.7 (50-74)	168 ± 5.7 (159-182)	no	no	no	test	Patel 2011 [47]
10	po (*tab*), bid	8	0	20 (19-22)	71 (60-83)		no	no	no	test	Smith 1987 [48]
10	po (tabER), qd	15	0	(18-38)			no	no	no	test	Blychert 1990a [17]
10	po (tabER°), qd	12	0	26 ± 1	76 ± 6		no	no	no	training	Lundahl 1998 [43]
20	po (tabER°), sd	12	0	27 (23-30)	76 (68-86)		no	yes	yes	training	Hasselgren 1990 [40]
40	po (tabER°), sd	12	0	27 (23-30)	76 (68-86)		no	yes	yes	training	Hasselgren 1990 [40]
**Hypertensive individuals[c]**											
1[d]	iv (15 min)	7	0	45 (32-57)	75 (68-91)		no	no	no	test	Edgar 1989 [49][f]
2.25[e]	iv (105-120 min)	12	8	64 ±2	87.75		no	yes	yes	test	Blychert 1990 [50]
0.83	po (sol), sd	12	25	59 ± 2.6 (39-68)	83 ± 3.6 (62-108)		no	no	yes	test	Edgar 1985 [11]
8.3	po (sol), sd	12	25	59 ± 2.6 (39-68)	83 ± 3.6 (62-108)		no	no	yes	test	Edgar 1985 [11]
10	po (*tab*), sd	6	33	46 (38-49)	91 (54-113)		no	no	yes	test	Larsson 1990 [51]
10	po (*tab*), sd	12	17	59 (36-74)	80 (54-99)		no	no	yes	test	Larsson 1990 [51][f]
10	po (*tab*), D1: 10mg sd, D2-D6: 5mg bid, D7-D29: 10mg bid	4	20	46.75 (38-49)	99.75 (78-113)		no	no	yes	test	Larsson 1990 [51]

assumed

[a]: mean ± standard deviation (range)

[c]: Patients were usually receiving other hypertensive medication, including beta blockers and diuretics

[d]: Doses between 0.5 - 1.5 mg were administered

[e]: 0.02 mg/min, for 105-120 min

[f]: Patients with renal impairment

° tablet brand: Plendil, * tablet brand: Hydac

- : no data available, bid: twice daily, BP: blood pressure; D: day; DHF: dehydrofelodipine, HR: heart rate, iv: intravenous, n: number of individuals, po: oral, qd: once daily, sd: single dose, sol: solution, tab: tablet, tabER: extended release tablet

Table S1: Clinical studies used for the development of the felodipine PBPK/PD model *(continued)*

Dose [mg]	Route	n	Women [%]	Age[a] [y]	Weight[a] [kg]	Height[a] [cm]	DHF	HR	BP	Dataset	Reference
10	po (*tab*), D1: 10mg sd, D2-D6: 5mg bid, D7-D29: 10mg bid	12	17	58.8 (36-74)	80.25 (54-99)		no	no	yes	test	Larsson 1990 [51][f]
10	po (tab), bid	10	10	61 ±1	88 ±4	175 ±2	no	no	no	test	Hedner 1987 [52]
2.5, 5	D1-D7: po (*tab*), bid; D8-D14: po (*tab*), bid	11	73	74 (67-79)	71 (61-89)		no	no	no	test	Landahl 1988 [22]
5	po (tabER), qd	28	64	41 ± 2 (22-50)	86 ± 4		no	no	yes	test	Leenen 2010 [53]
5	po (tabER), qd	35	71	67 ± 1 (60-77)	79 ± 2		no	no	yes	test	Leenen 2010 [53]
5, 10	D1-D14: po (*tab*), bid; D15-D28: po (*tab*), bid	12	0	57 (52-63)			no	yes	yes	test	Hedner 1986 [54]
20	po (tabER), qd	9	0	64 ±3	89 ±5	176 ±3	no	no	no	test	Hedner 1987 [52]
20	po (tabER), sd	12	8	64 ±2	88		no	yes	yes	test	Blychert 1990 [50]
20	po (tabER), qd	12	8	64 ±2	88		no	yes	yes	test	Blychert 1990 [50]

assumed

[a]: mean ± standard deviation (range)

[c]: Patients were usually receiving other hypertensive medication, including beta blockers and diuretics

[d]: Doses between 0.5 - 1.5 mg were administered

[e]: 0.02 mg/min, for 105-120 min

[f]: Patients with renal impairment

° tablet brand: Plendil, * tablet brand: Hydac

- : no data available, bid: twice daily, BP: blood pressure; D: day; DHF: dehydrofelodipine, HR: heart rate, iv: intravenous, n: number of individuals, po: oral, qd: once daily, sd: single dose, sol: solution, tab: tablet, tabER: extended release tablet

2.3 Felodipine drug-dependent parameters

Table S2: Drug-dependent parameters of the final felodipine parent-metabolite PBPK model

Parameter	Unit	Value (model)	Value (literature)	Reference	Description
Felodipine					
MW	g/mol	384.25 (lit)	384.25	[55]	Molecular weight
fu	%	0.36 (lit)	0.36	[56]	Fraction unbound in plasma
Solubility (pH)	mg/l	7.15 (7) (lit)	0.5 (6.5); 1.2 (7); 7.15 (7); 14.3 (7.1); 19.7 (7)	[57–59]	Solubility
Solubility tabER (pH)	mg/l	0.89 (7.0) (opt)	-	-	Solubility (extended release tablets)
logP		4.36 (lit)	3.44; 3.80; 4.36; 4.46; 4.64	[55, 58–60]	Lipophilicity
Intestinal permeability	cm/min	2.76E-4 (opt)	4.42E-4; 3.06E-4; 2.64E-4	[59, 61]	Transcellular intestinal permeability
GFR fraction		1 (asm)	-	-	Fraction of filtered drug in the urine
K_M (CYP3A4)	µmol/l	2.81 (lit)	0.648; 0.94; 2.81; 26.4	[59, 62, 63]	CYP3A4 Michaelis-Menten constant
K_M (CYP3A4) - (R)-Felodipine	µmol/l	2.16 (opt)	6.1 (3.4 - 8.7)	[5]	CYP3A4 Michaelis-Menten constant
K_M (CYP3A4) - (S)-Felodipine	µmol/l	4.27 (opt)	12.2 (5.9 - 21.6)	[5]	CYP3A4 Michaelis-Menten constant
k_{cat} (CYP3A4)	1/min	250.44 (opt)			CYP3A4 catalytic rate constant
s (tab)		1.32(opt)	-	-	Dissolution profile shape
$t_{50\%}$ (tab)	min	54.86 (opt)	-	-	Dissolution time (50% dissolved)
s (tabER)		1.30 (opt)	-	-	Dissolution profile shape
$t_{50\%}$ (tabER)	min	173.04 (opt)	-	-	Dissolution time (50% dissolved)
Partition coefficient	-	diverse	Rodgers and Rowland	[64, 65]	Cell to plasma partition coefficients
Cellular permeability	cm/min	0.42 (calc)	PK-Sim Standard	[66]	Permeability into the cellular space
Dehydrofelodipine					
MW	g/mol	382.2 (lit)	382.2	[67]	Molecular weight
pKa (base)	-	4.06 (lit)	4.06	[67]	Acid dissociation constant
f_u	%	0.68 (lit)	-	-	Fraction unbound in plasma
Solubility (pH)	mg/l	2.93 (7) (lit)	2.93 (7)	[67]	Solubility
logP		3.32 (opt)	4.24	[67]	Lipophilicity
CL (CYP3A4)	1/min	35.74 (opt)	-	-	CYP3A4 clearance process
CL_{hep}	1/min	2.76 (opt)	-	-	Unspecific hepatic clearance process
Intestinal permeability	cm/min	1.38E-4 (calc)	-	-	Transcellular intestinal permeability
GFR fraction		1 (asm)	-	-	Fraction of filtered drug in the urine
Partition coefficient	-	diverse	Schmitt	[68]	Cell to plasma partition coefficients
Cellular permeability	cm/min	0.04 (calc)	Charge dependent Schmitt	[1]	Permeability into the cellular space

asm: assumed, calc: calculated, CYP3A4: cytochrom P450 3A4, GFR: glomerular filtration rate, lit: literature value, opt: optimized value, tab: tablet, tabER: extended release tablet

2.4 Plasma concentration-time profiles

2.4.1 Linear

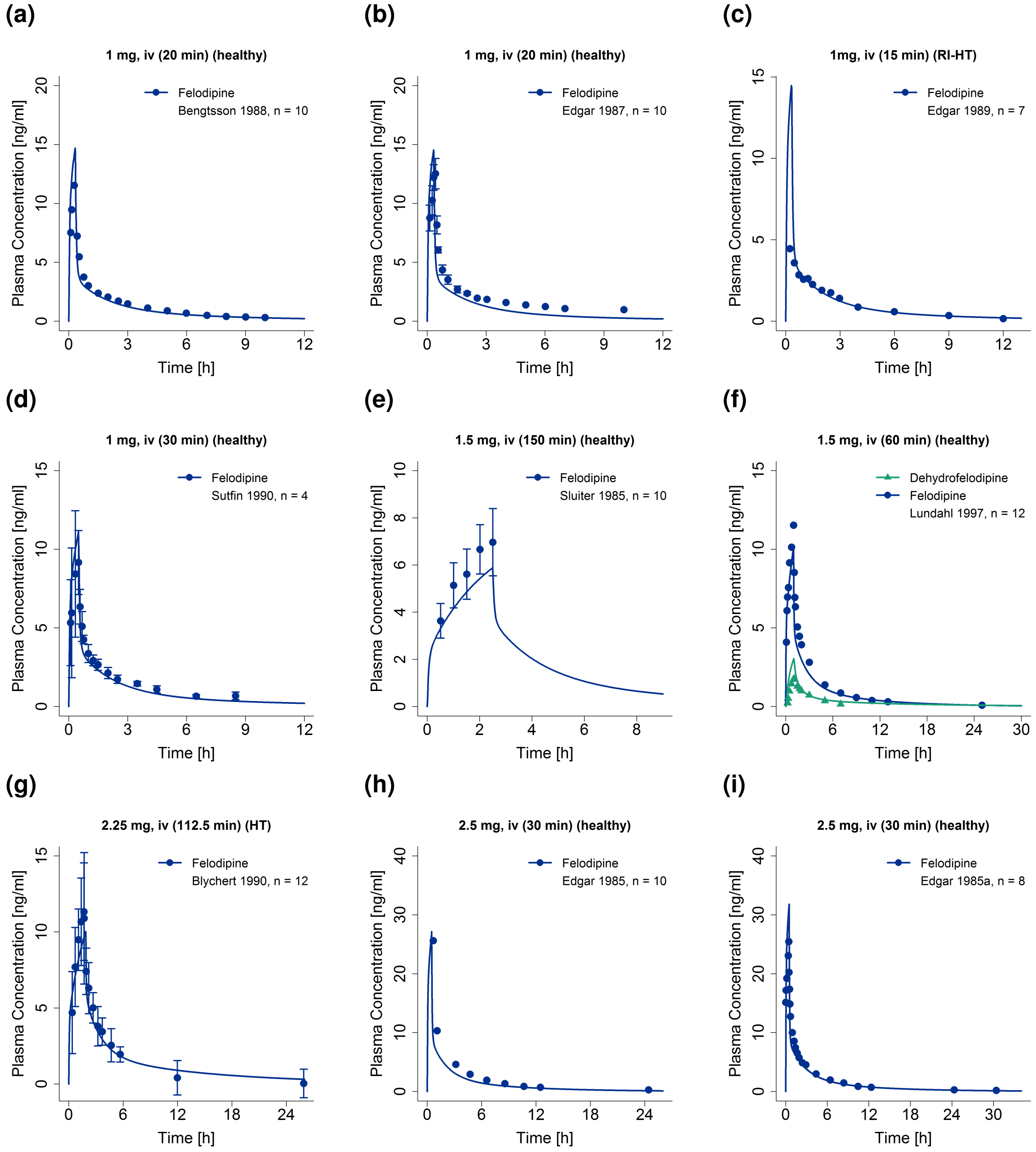

Figure S2: Predicted compared to observed felodipine and dehydrofelodipine plasma concentration-time profiles (linear) after intravenous and oral administration of felodipine. Observed data are shown as dots and triangles ± standard deviation (if available); model predictions are shown as solid lines. Details on dosing regimens, study populations and literature references are listed in Table S1. bid: twice daily, HT: hypertension, iv: intravenous, md: multiple dose, n: number of individuals, qd: once daily, RI: renal impairment, sol: solution, sd: single dose, tab: tablet, tabER: extended release tablet.

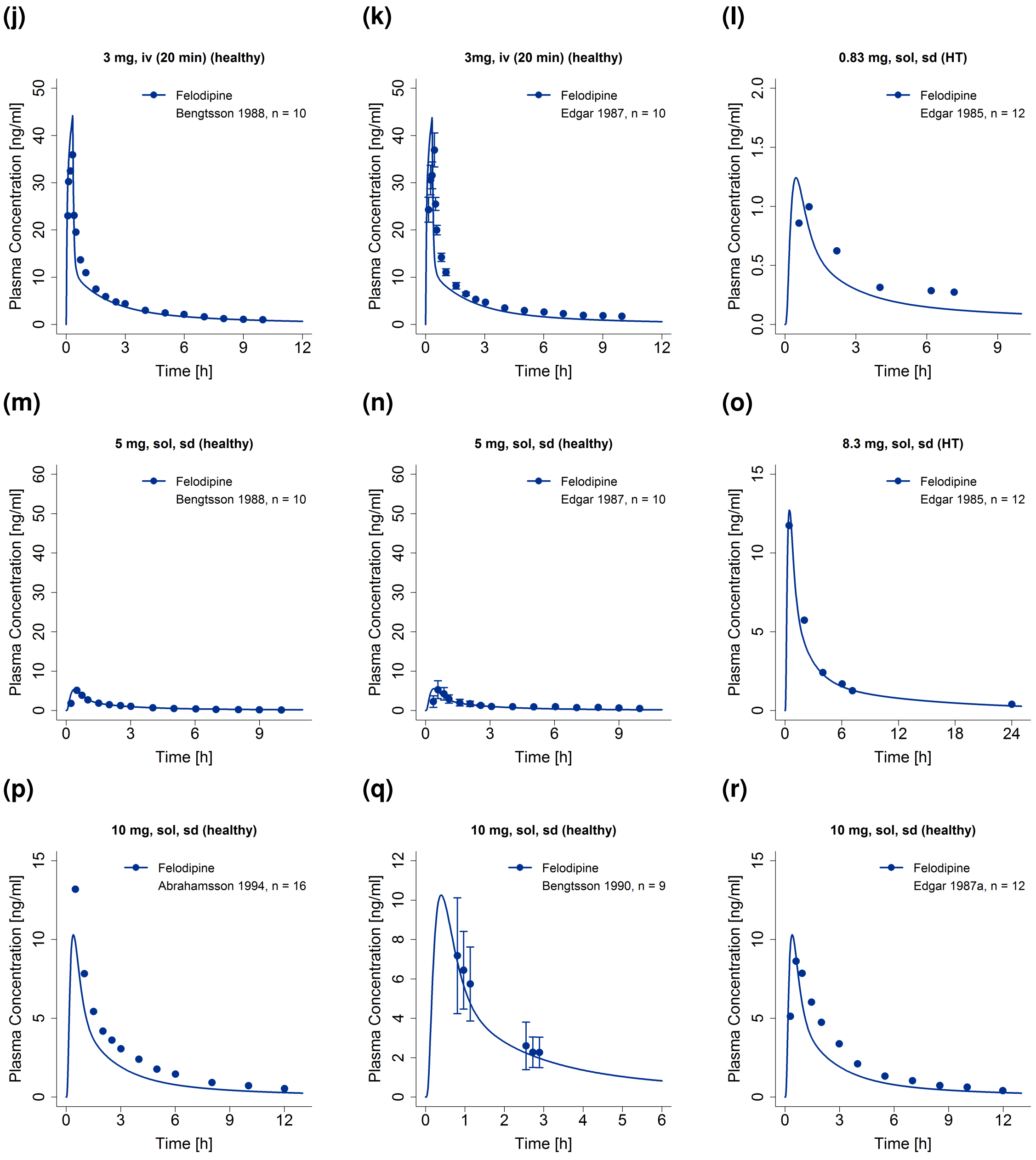

Figure S2: Predicted compared to observed felodipine and dehydrofelodipine plasma concentration-time profiles (linear) after intravenous and oral administration of felodipine. Observed data are shown as dots and triangles ± standard deviation (if available); model predictions are shown as solid lines. Details on dosing regimens, study populations and literature references are listed in Table S1. bid: twice daily, HT: hypertension, iv: intravenous, md: multiple dose, n: number of individuals, qd: once daily, RI: renal impairment, sol: solution, sd: single dose, tab: tablet, tabER: extended release tablet. *(continued)*

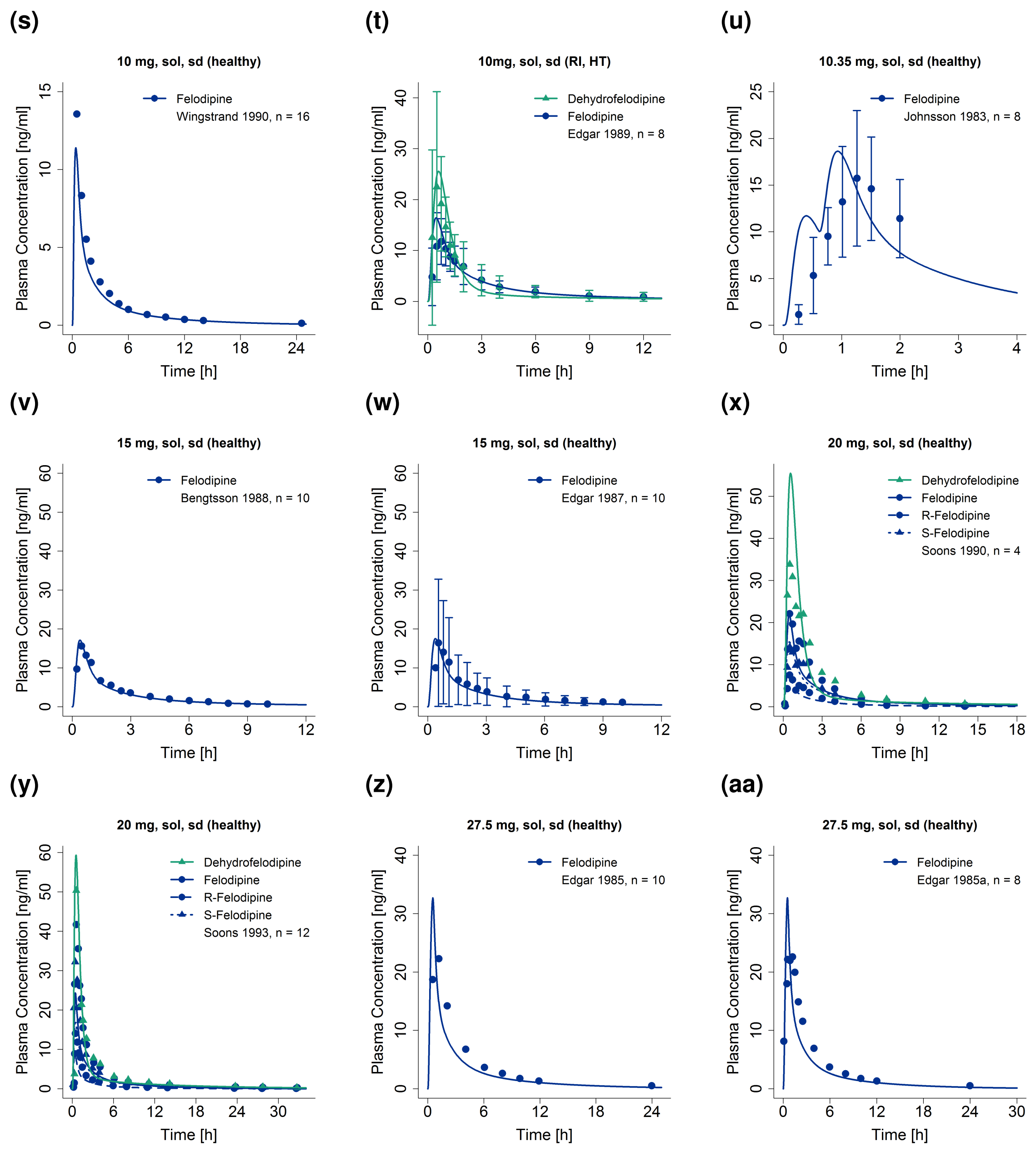

Figure S2: Predicted compared to observed felodipine and dehydrofelodipine plasma concentration-time profiles (linear) after intravenous and oral administration of felodipine. Observed data are shown as dots and triangles ± standard deviation (if available); model predictions are shown as solid lines. Details on dosing regimens, study populations and literature references are listed in Table S1. bid: twice daily, HT: hypertension, iv: intravenous, md: multiple dose, n: number of individuals, qd: once daily, RI: renal impairment, sol: solution, sd: single dose, tab: tablet, tabER: extended release tablet. *(continued)*

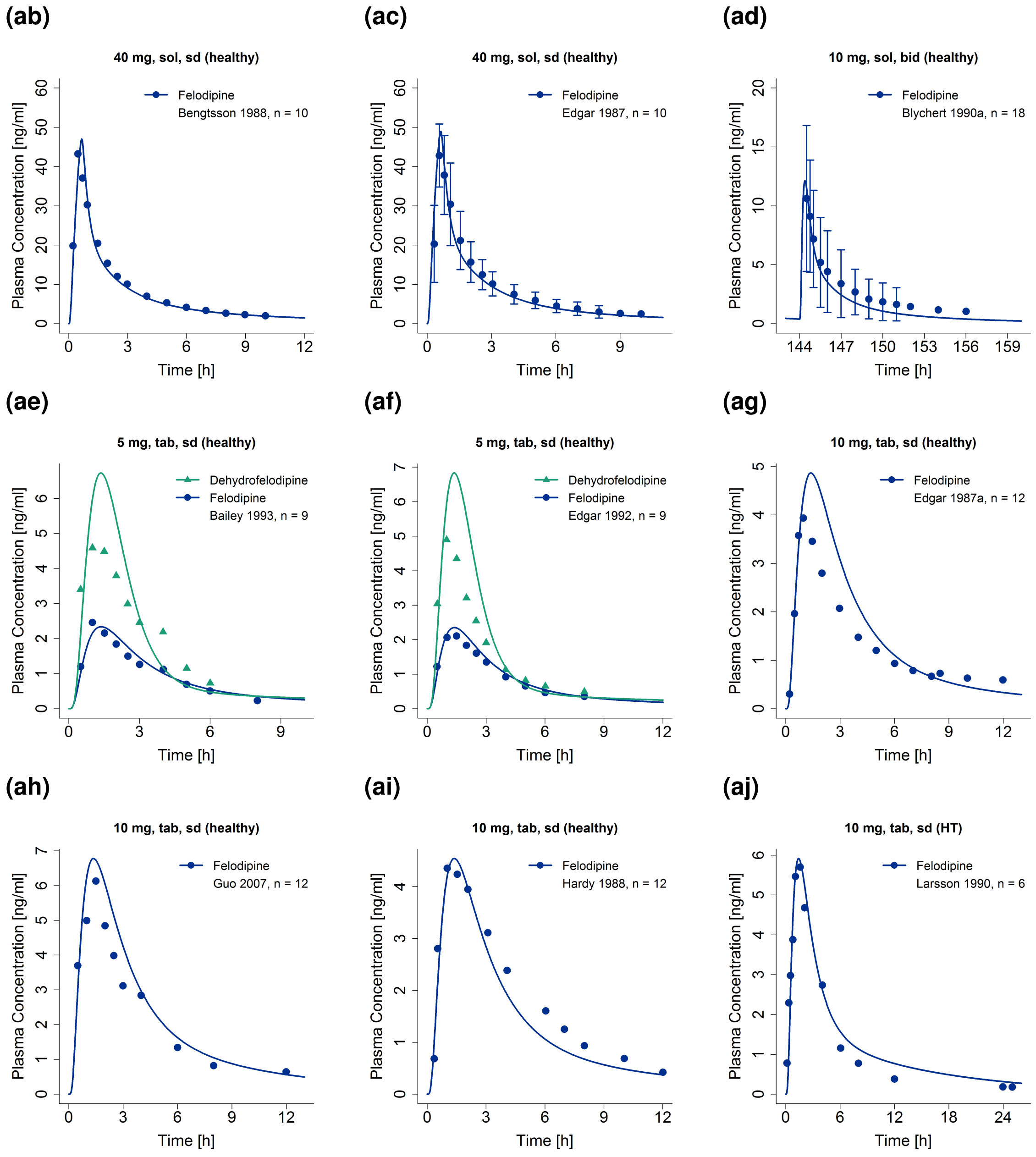

Figure S2: Predicted compared to observed felodipine and dehydrofelodipine plasma concentration-time profiles (linear) after intravenous and oral administration of felodipine. Observed data are shown as dots and triangles ± standard deviation (if available); model predictions are shown as solid lines. Details on dosing regimens, study populations and literature references are listed in Table S1. bid: twice daily, HT: hypertension, iv: intravenous, md: multiple dose, n: number of individuals, qd: once daily, RI: renal impairment, sol: solution, sd: single dose, tab: tablet, tabER: extended release tablet. *(continued)*

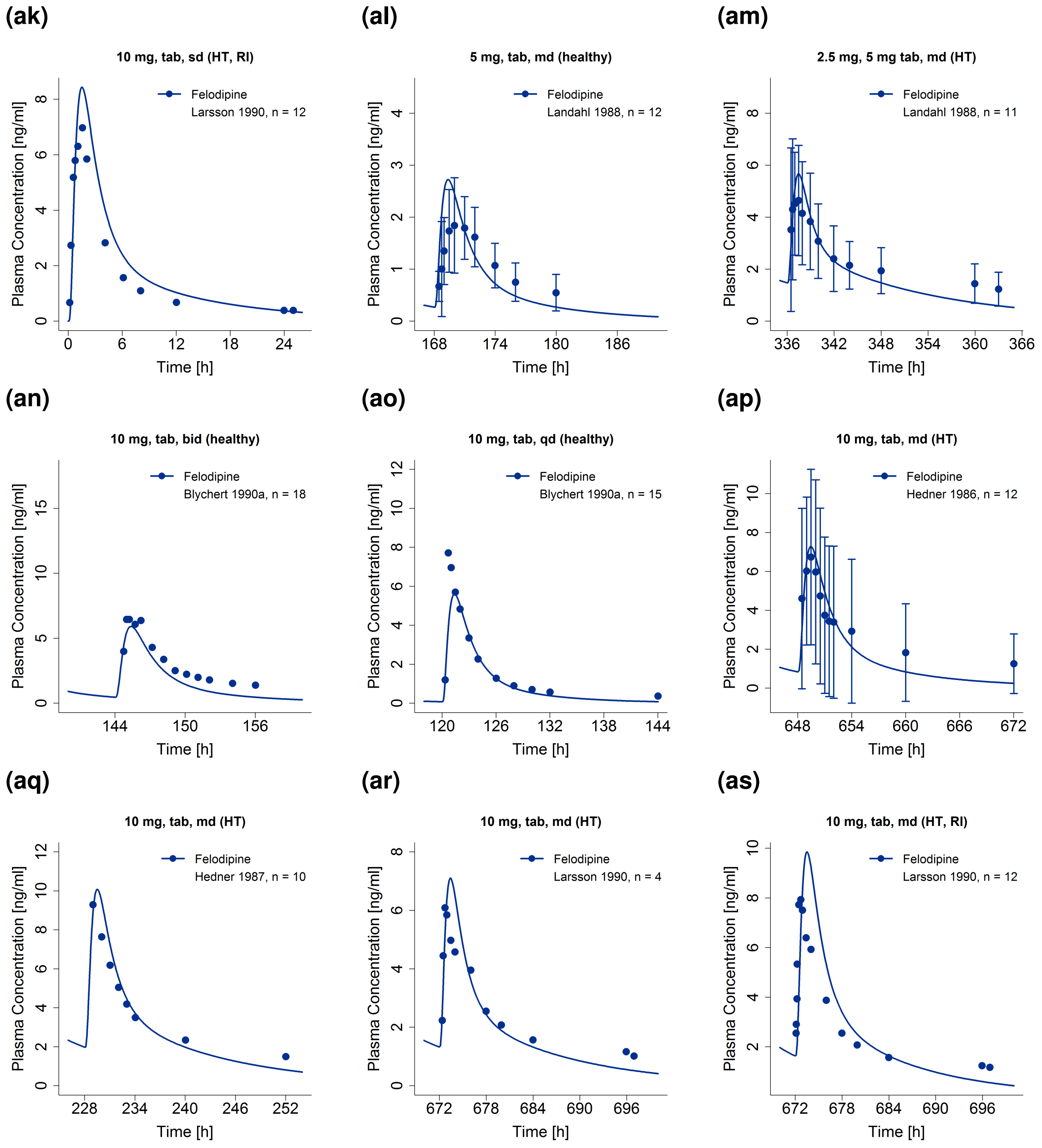

Figure S2: Predicted compared to observed felodipine and dehydrofelodipine plasma concentration-time profiles (linear) after intravenous and oral administration of felodipine. Observed data are shown as dots and triangles ± standard deviation (if available); model predictions are shown as solid lines. Details on dosing regimens, study populations and literature references are listed in Table S1. bid: twice daily, HT: hypertension, iv: intravenous, md: multiple dose, n: number of individuals, qd: once daily, RI: renal impairment, sol: solution, sd: single dose, tab: tablet, tabER: extended release tablet. *(continued)*

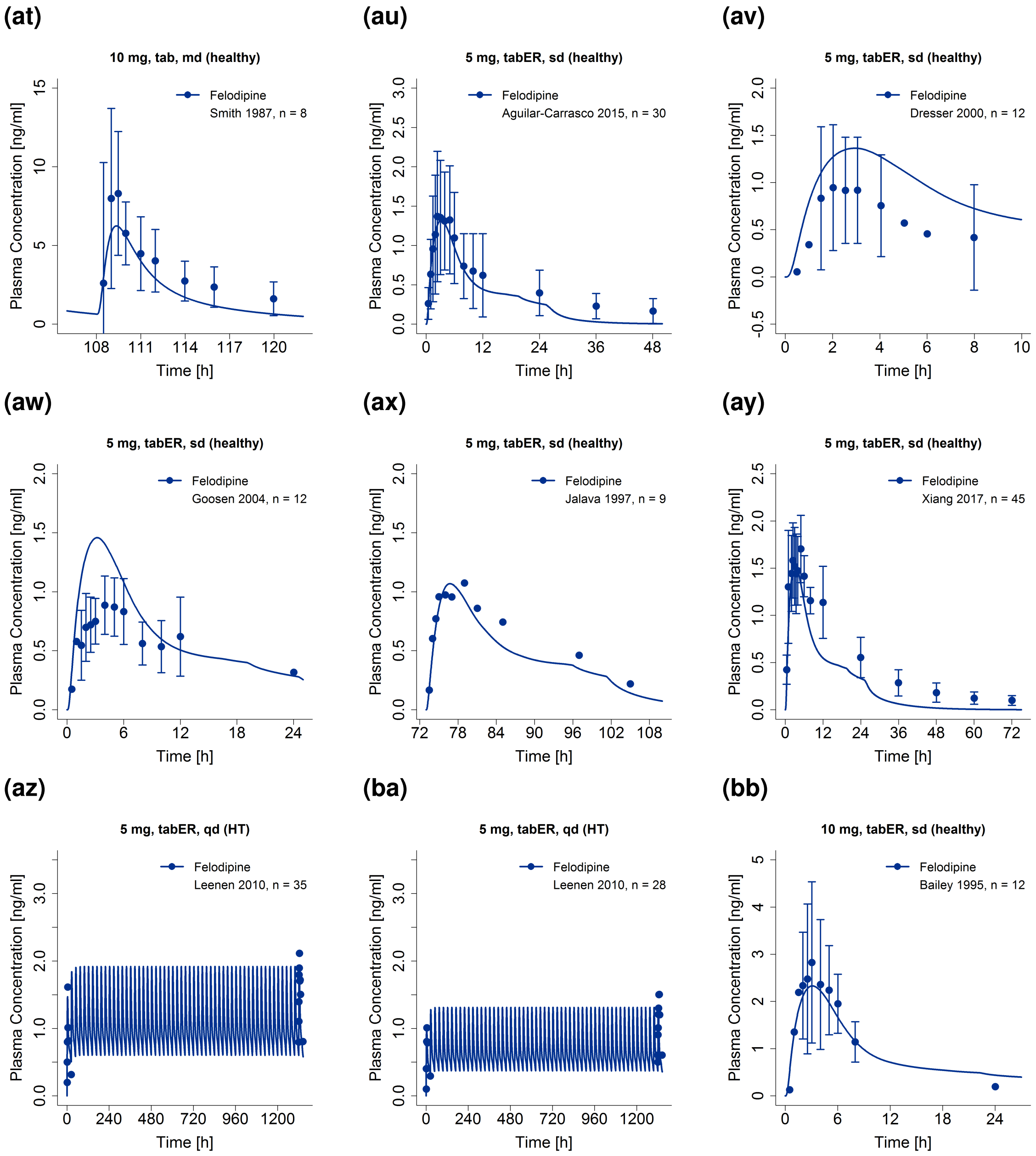

Figure S2: Predicted compared to observed felodipine and dehydrofelodipine plasma concentration-time profiles (linear) after intravenous and oral administration of felodipine. Observed data are shown as dots and triangles ± standard deviation (if available); model predictions are shown as solid lines. Details on dosing regimens, study populations and literature references are listed in Table S1. bid: twice daily, HT: hypertension, iv: intravenous, md: multiple dose, n: number of individuals, qd: once daily, RI: renal impairment, sol: solution, sd: single dose, tab: tablet, tabER: extended release tablet. *(continued)*

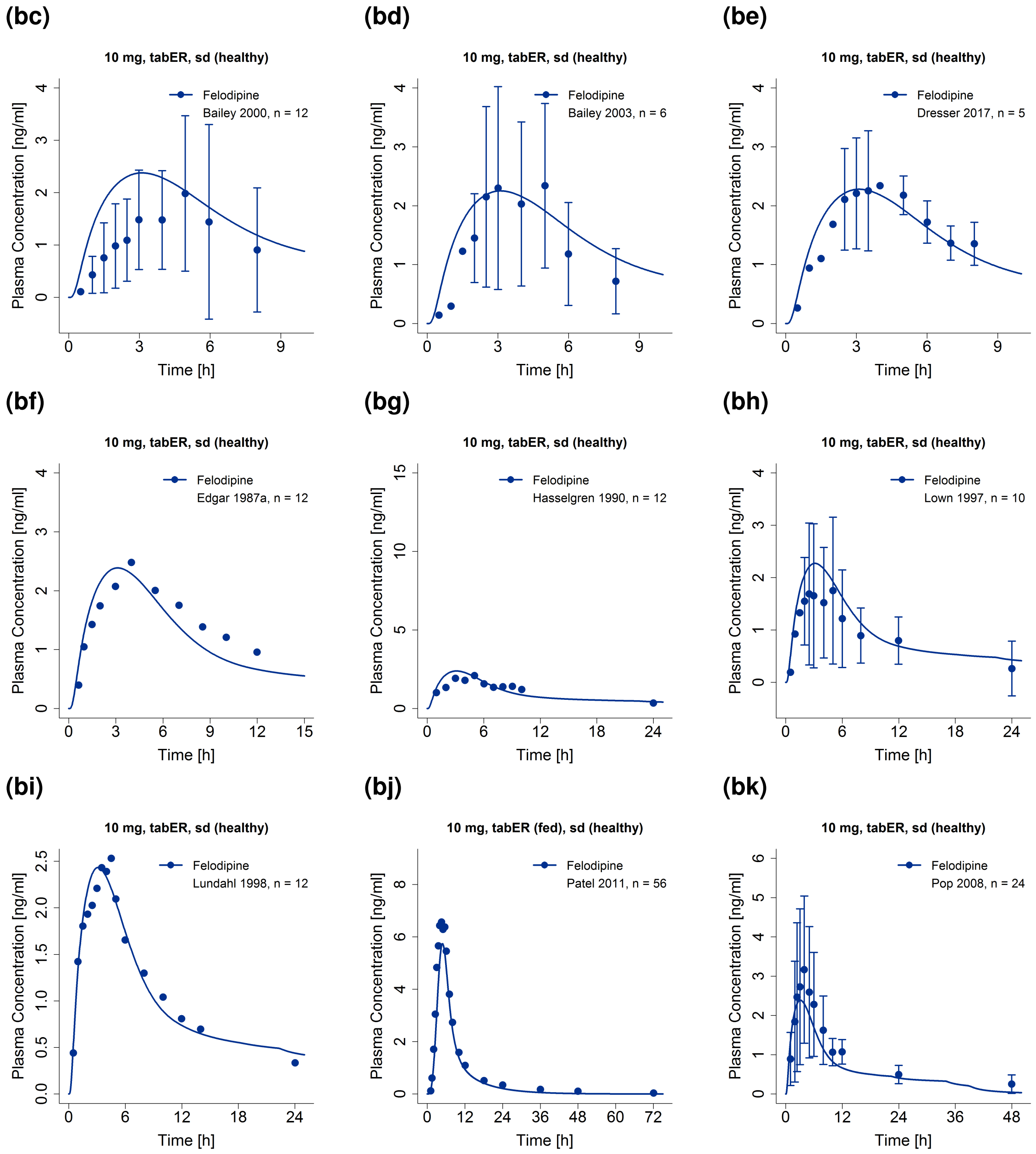

Figure S2: Predicted compared to observed felodipine and dehydrofelodipine plasma concentration-time profiles (linear) after intravenous and oral administration of felodipine. Observed data are shown as dots and triangles ± standard deviation (if available); model predictions are shown as solid lines. Details on dosing regimens, study populations and literature references are listed in Table S1. bid: twice daily, HT: hypertension, iv: intravenous, md: multiple dose, n: number of individuals, qd: once daily, RI: renal impairment, sol: solution, sd: single dose, tab: tablet, tabER: extended release tablet. *(continued)*

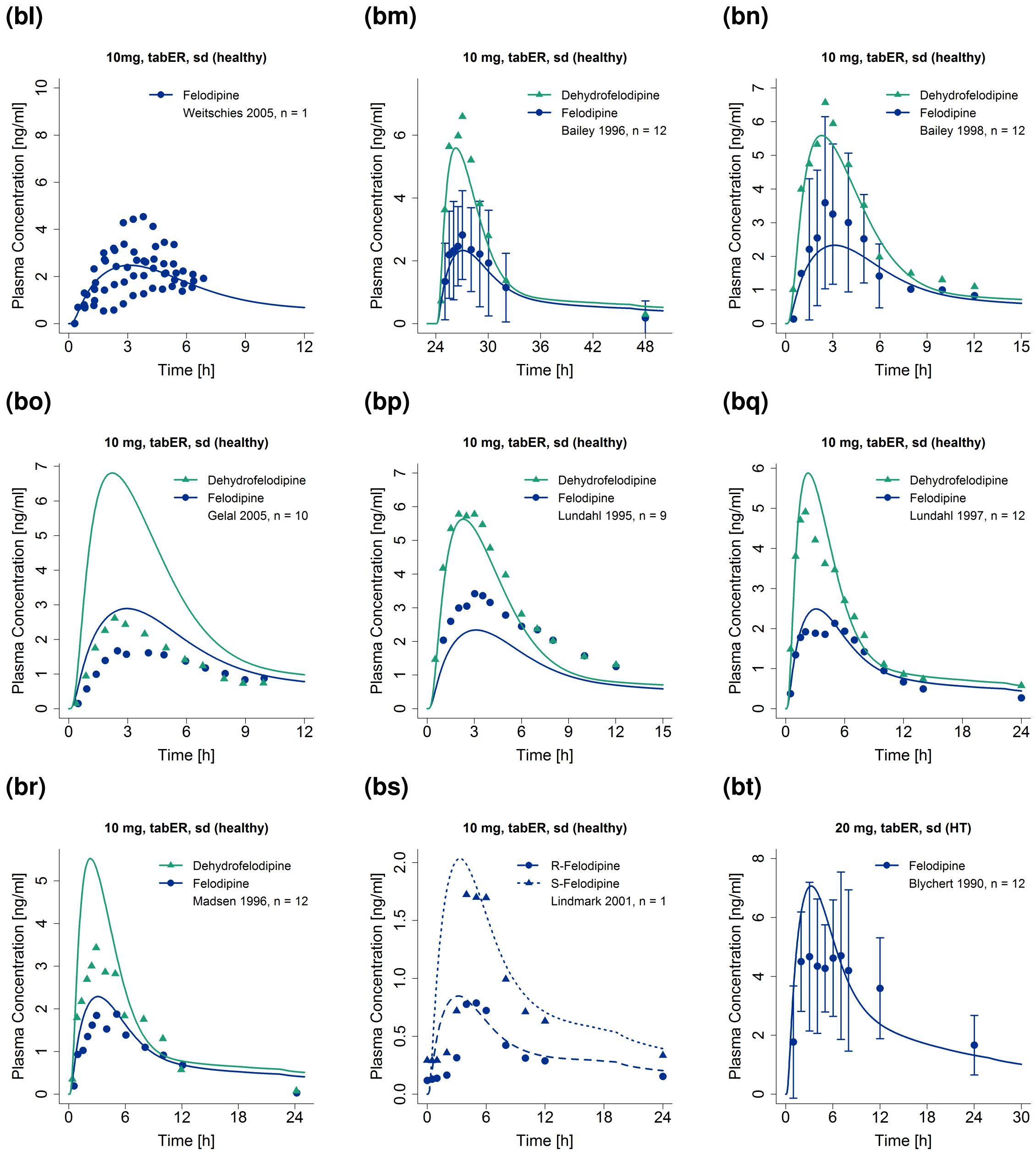

Figure S2: Predicted compared to observed felodipine and dehydrofelodipine plasma concentration-time profiles (linear) after intravenous and oral administration of felodipine. Observed data are shown as dots and triangles ± standard deviation (if available); model predictions are shown as solid lines. Details on dosing regimens, study populations and literature references are listed in Table S1. bid: twice daily, HT: hypertension, iv: intravenous, md: multiple dose, n: number of individuals, qd: once daily, RI: renal impairment, sol: solution, sd: single dose, tab: tablet, tabER: extended release tablet. *(continued)*

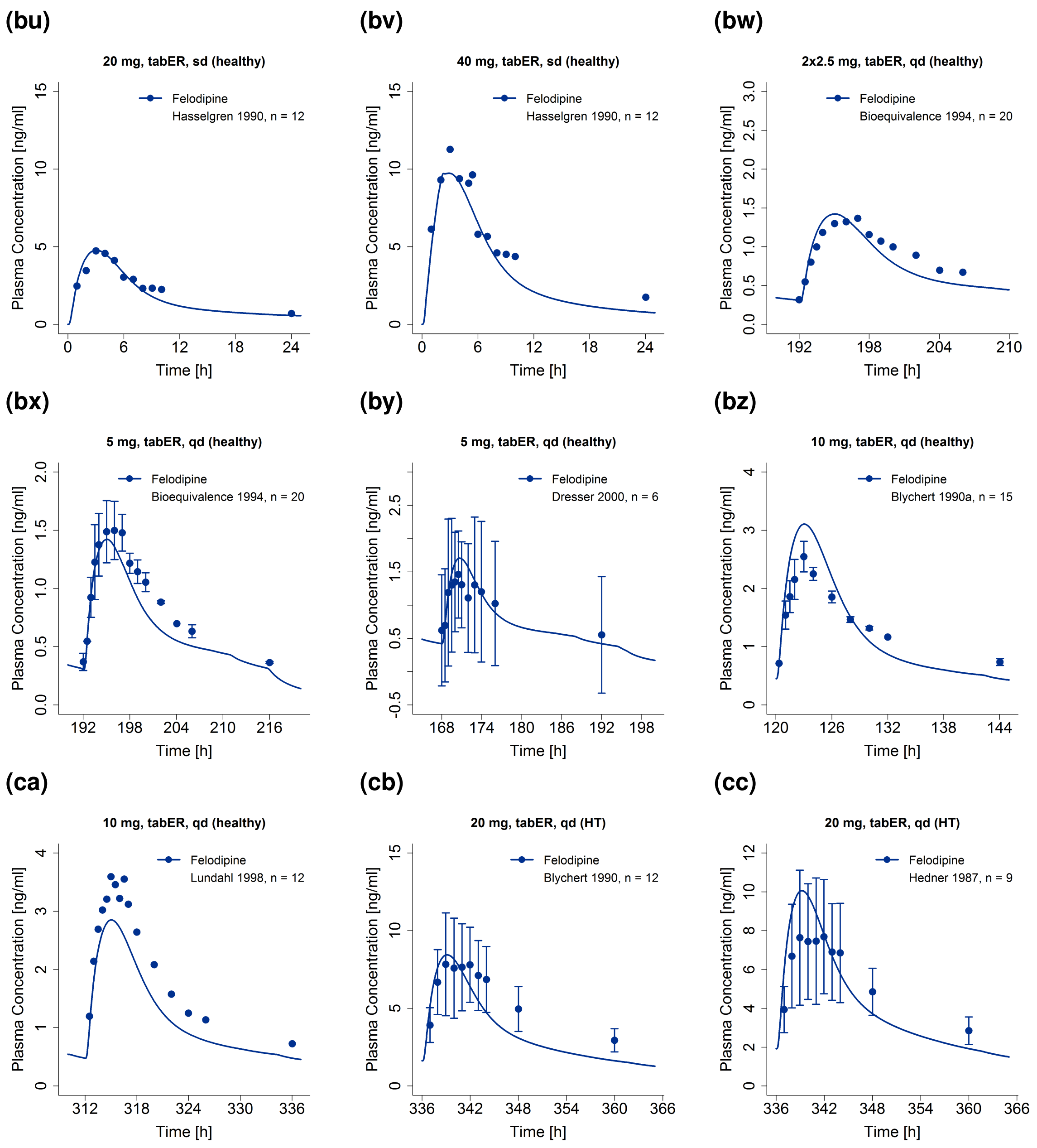

Figure S2: Predicted compared to observed felodipine and dehydrofelodipine plasma concentration-time profiles (linear) after intravenous and oral administration of felodipine. Observed data are shown as dots and triangles ± standard deviation (if available); model predictions are shown as solid lines. Details on dosing regimens, study populations and literature references are listed in Table S1. bid: twice daily, HT: hypertension, iv: intravenous, md: multiple dose, n: number of individuals, qd: once daily, RI: renal impairment, sol: solution, sd: single dose, tab: tablet, tabER: extended release tablet. *(continued)*

2.4.2 Semi-logarithmic

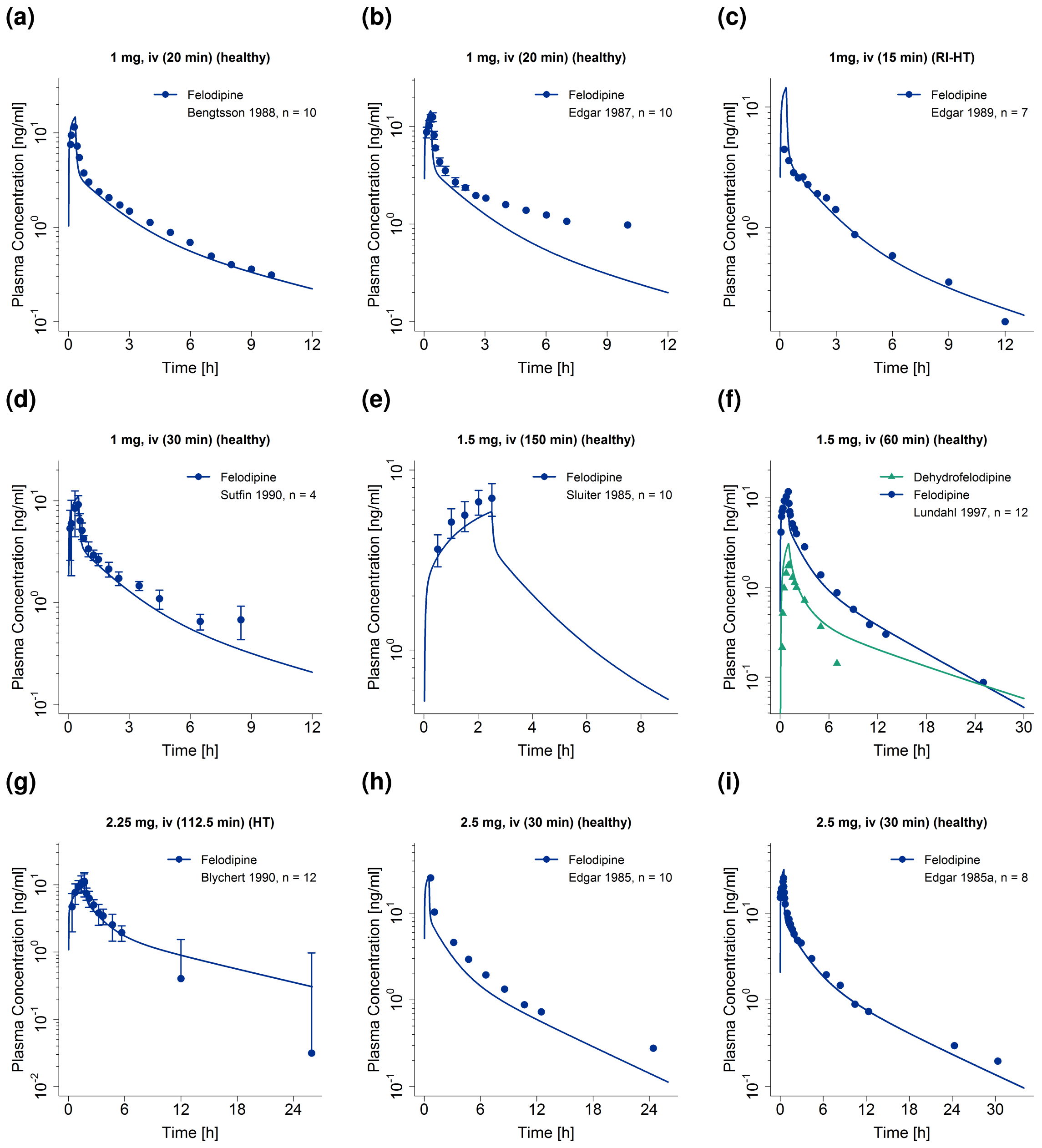

Figure S3: Predicted compared to observed felodipine and dehydrofelodipine plasma concentration-time profiles (semi-logarithmic) after intravenous and oral administration of felodipine. Observed data are shown as dots and triangles ± standard deviation; model predictions are shown as solid lines. Details on dosing regimens, study populations and literature references are listed in Table S1. bid: twice daily, HT: hypertension, iv: intravenous, md: multiple dose, n: number of individuals, qd: once daily, RI: renal impairment, sol: solution, sd: single dose, tab: tablet, tabER: extended release tablet.

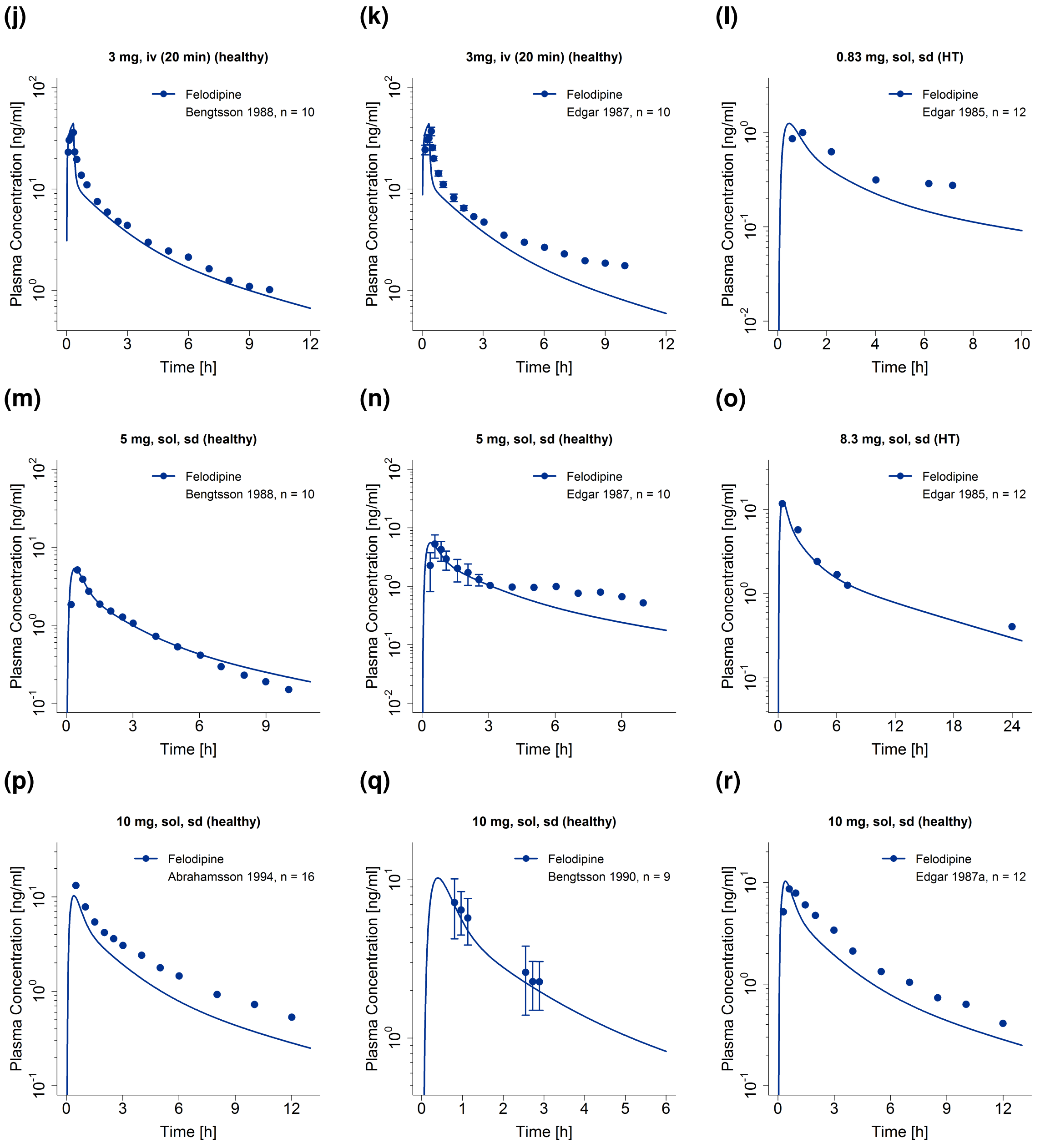

Figure S3: Predicted compared to observed felodipine and dehydrofelodipine plasma concentration-time profiles (semi-logarithmic) after intravenous and oral administration of felodipine. Observed data are shown as dots and triangles ± standard deviation; model predictions are shown as solid lines. Details on dosing regimens, study populations and literature references are listed in Table S1. bid: twice daily, HT: hypertension, iv: intravenous, md: multiple dose, n: number of individuals, qd: once daily, RI: renal impairment, sol: solution, sd: single dose, tab: tablet, tabER: extended release tablet. *(continued)*

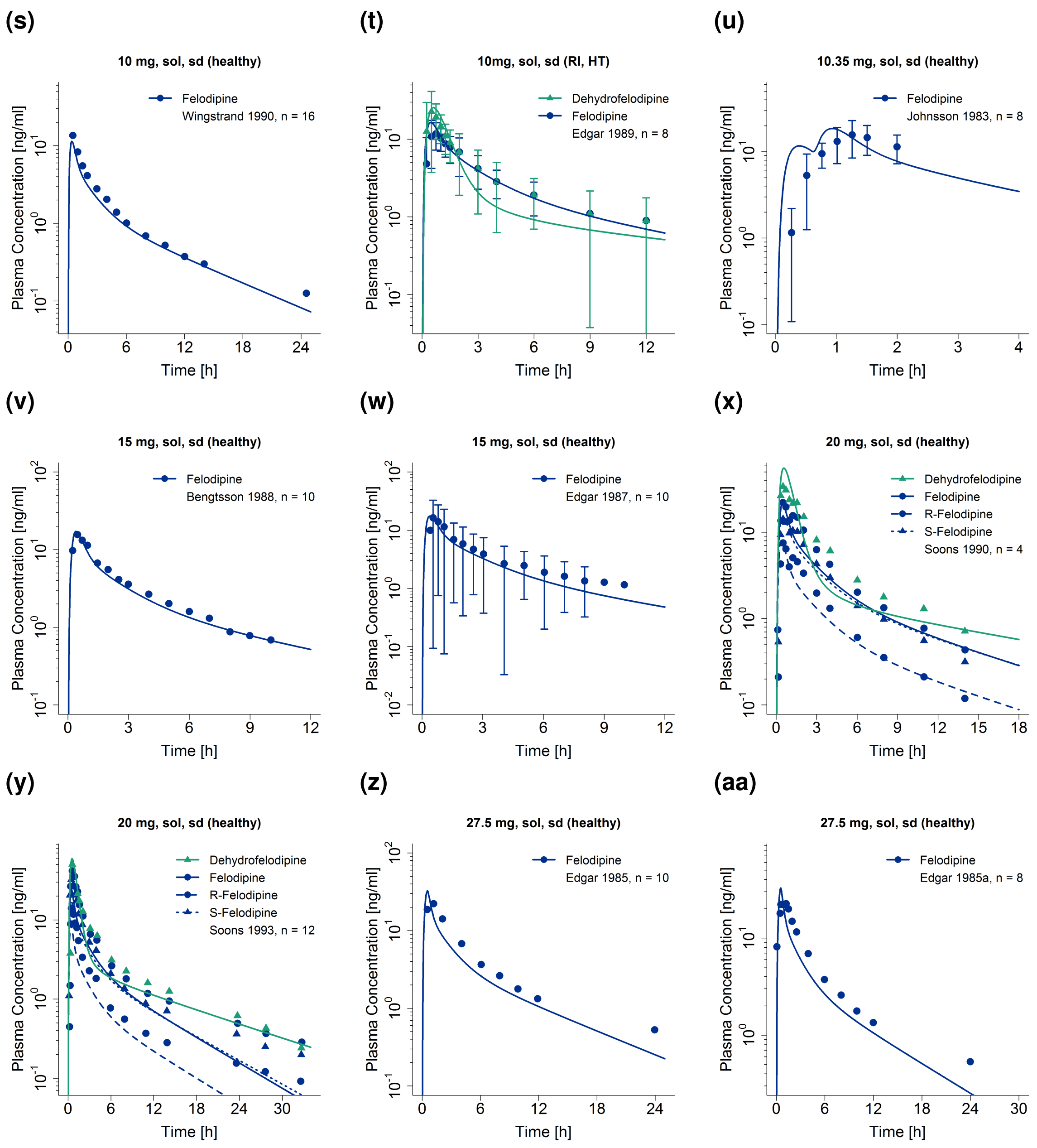

Figure S3: Predicted compared to observed felodipine and dehydrofelodipine plasma concentration-time profiles (semi-logarithmic) after intravenous and oral administration of felodipine. Observed data are shown as dots and triangles ± standard deviation; model predictions are shown as solid lines. Details on dosing regimens, study populations and literature references are listed in Table S1. bid: twice daily, HT: hypertension, iv: intravenous, md: multiple dose, n: number of individuals, qd: once daily, RI: renal impairment, sol: solution, sd: single dose, tab: tablet, tabER: extended release tablet. *(continued)*

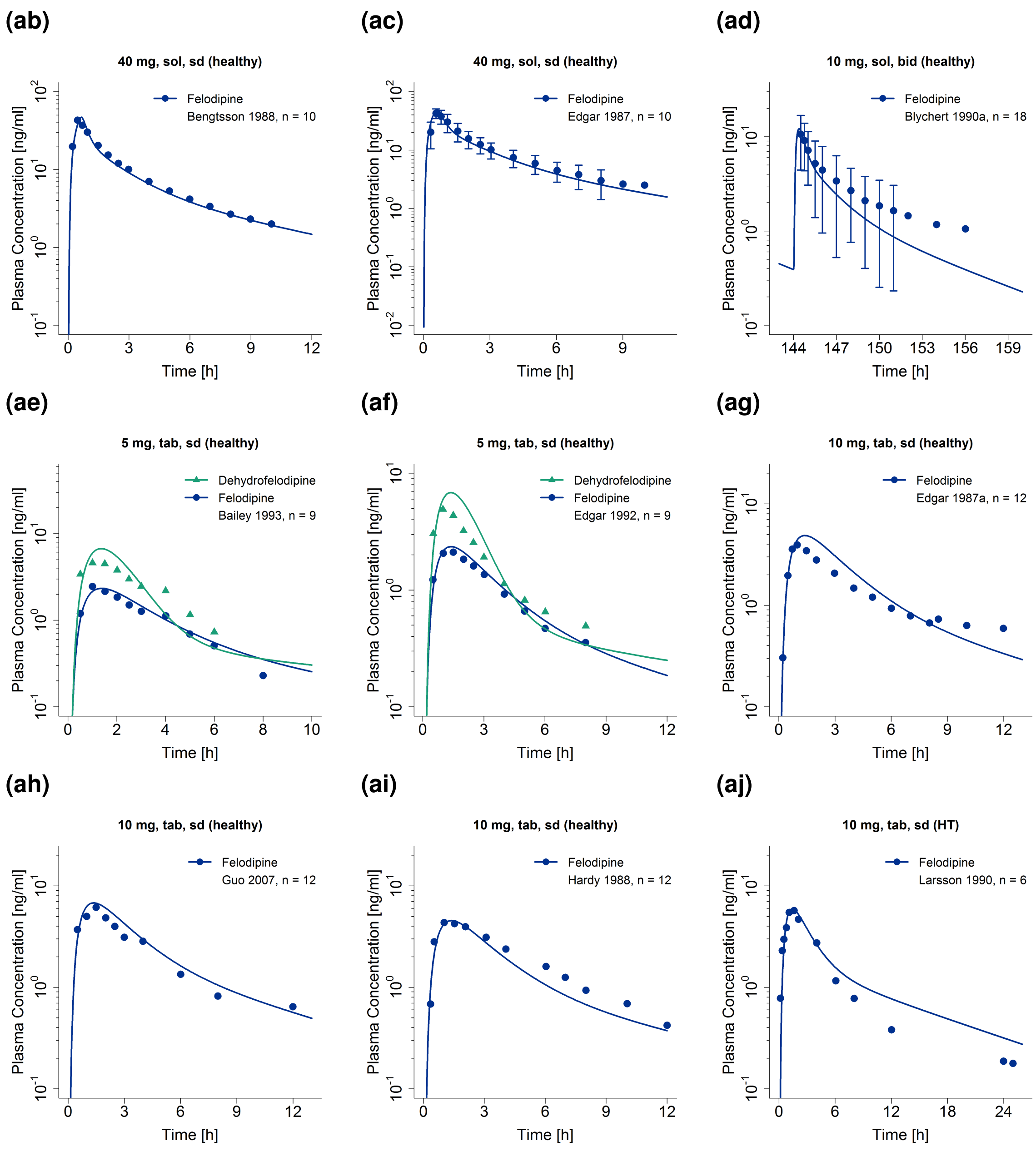

Figure S3: Predicted compared to observed felodipine and dehydrofelodipine plasma concentration-time profiles (semi-logarithmic) after intravenous and oral administration of felodipine. Observed data are shown as dots and triangles ± standard deviation; model predictions are shown as solid lines. Details on dosing regimens, study populations and literature references are listed in Table S1. bid: twice daily, HT: hypertension, iv: intravenous, md: multiple dose, n: number of individuals, qd: once daily, RI: renal impairment, sol: solution, sd: single dose, tab: tablet, tabER: extended release tablet. *(continued)*

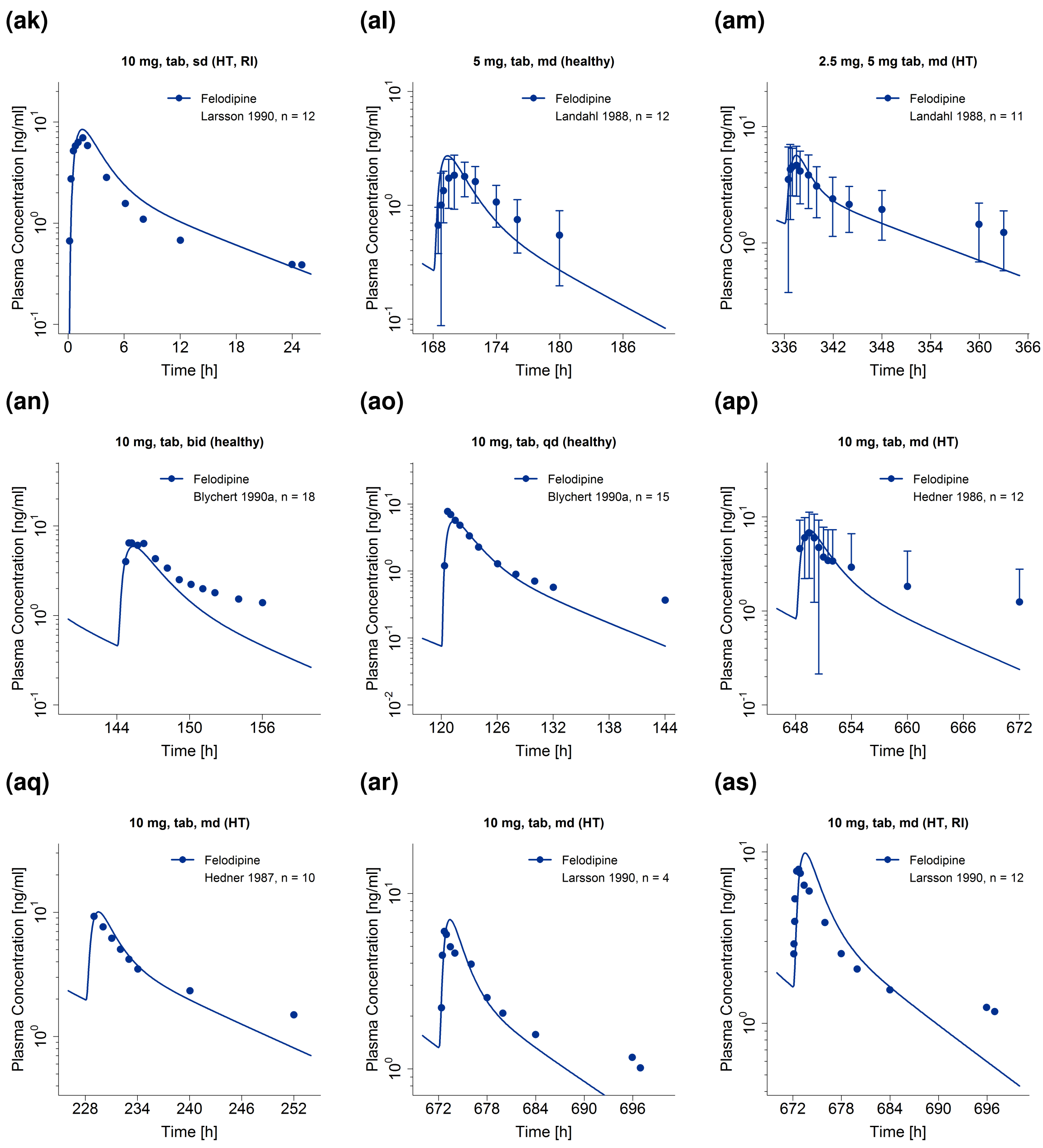

Figure S3: Predicted compared to observed felodipine and dehydrofelodipine plasma concentration-time profiles (semi-logarithmic) after intravenous and oral administration of felodipine. Observed data are shown as dots and triangles ± standard deviation; model predictions are shown as solid lines. Details on dosing regimens, study populations and literature references are listed in Table S1. bid: twice daily, HT: hypertension, iv: intravenous, md: multiple dose, n: number of individuals, qd: once daily, RI: renal impairment, sol: solution, sd: single dose, tab: tablet, tabER: extended release tablet. *(continued)*

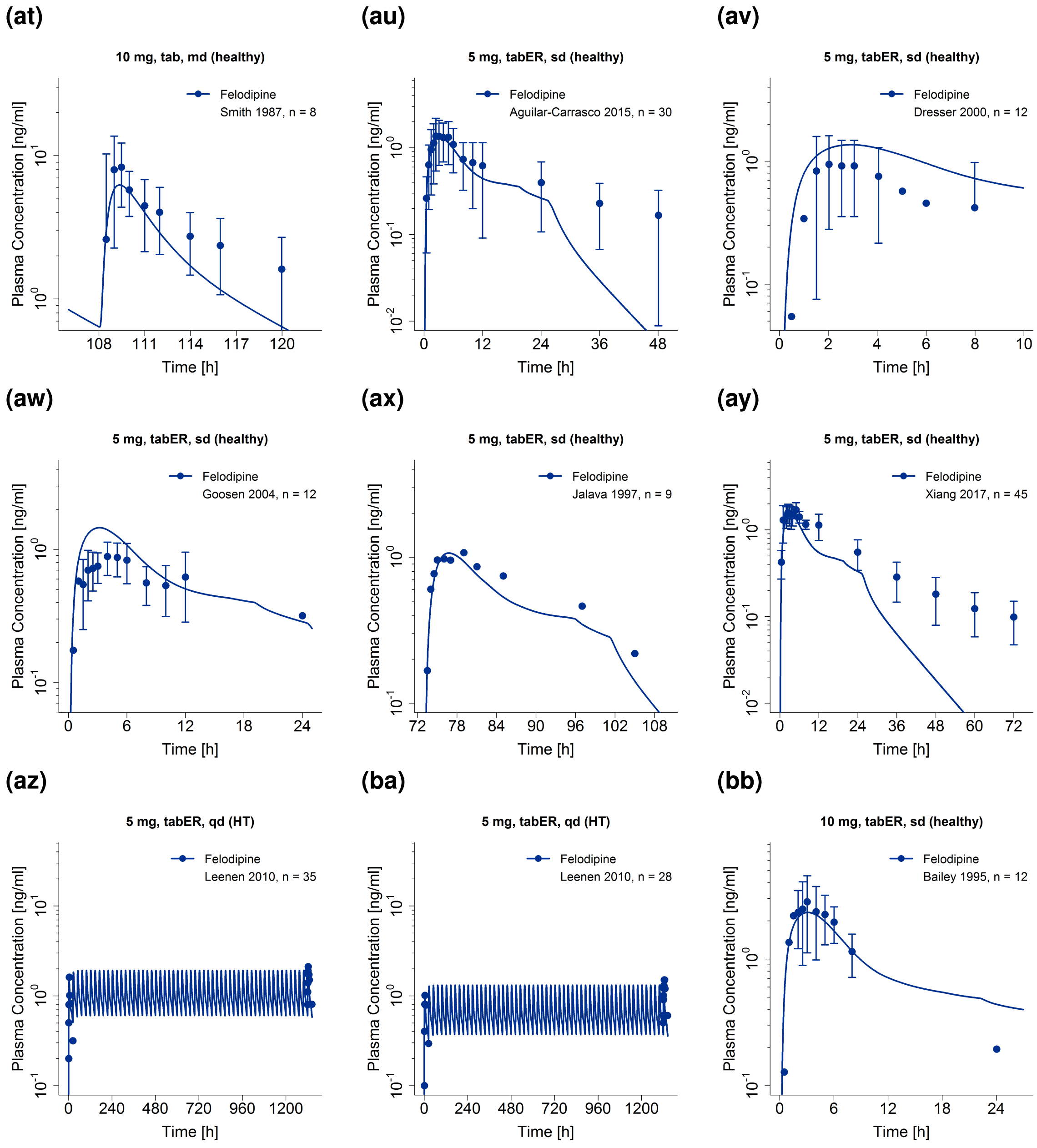

Figure S3: Predicted compared to observed felodipine and dehydrofelodipine plasma concentration-time profiles (semi-logarithmic) after intravenous and oral administration of felodipine. Observed data are shown as dots and triangles ± standard deviation; model predictions are shown as solid lines. Details on dosing regimens, study populations and literature references are listed in Table S1. bid: twice daily, HT: hypertension, iv: intravenous, md: multiple dose, n: number of individuals, qd: once daily, RI: renal impairment, sol: solution, sd: single dose, tab: tablet, tabER: extended release tablet. *(continued)*

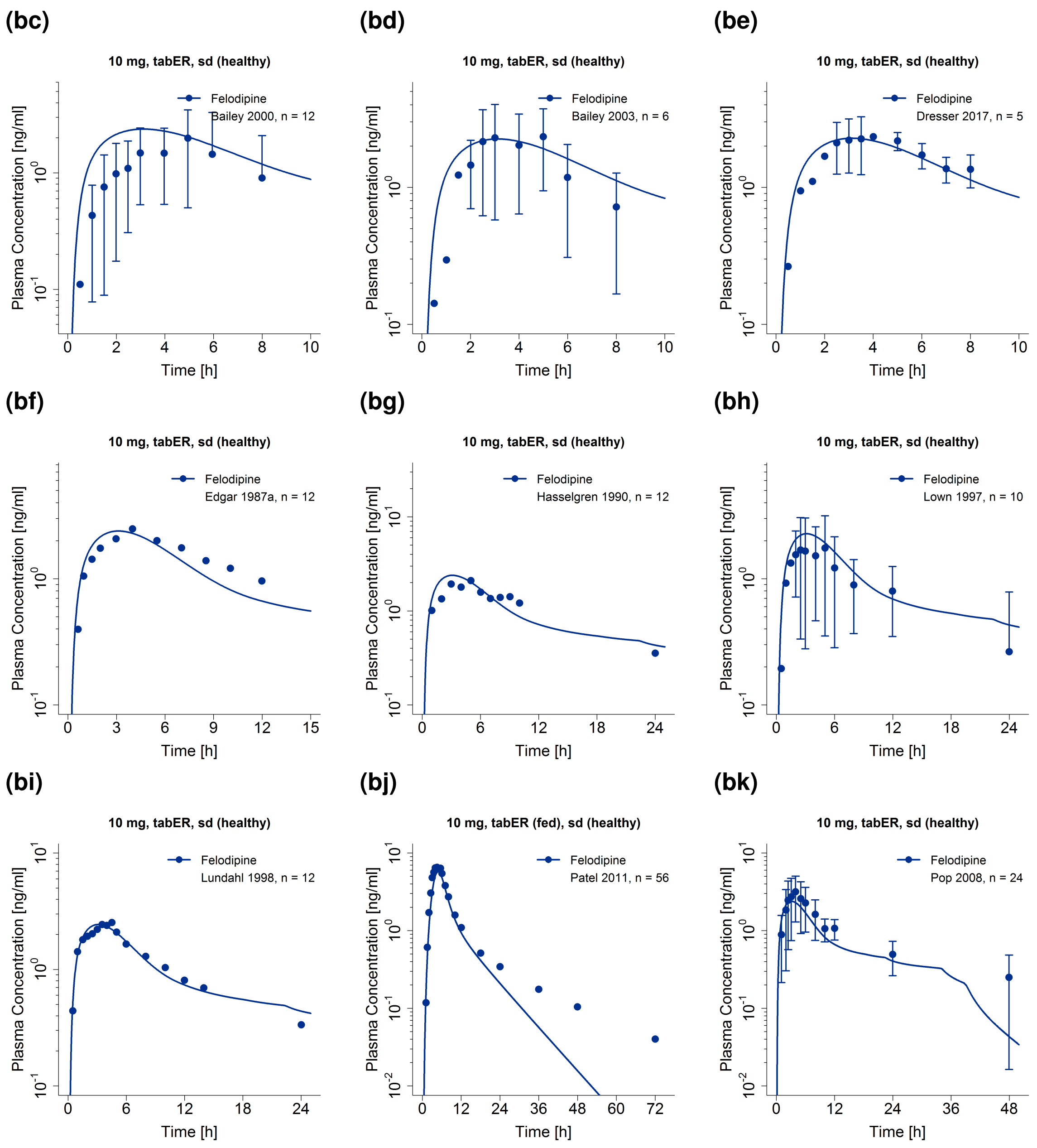

Figure S3: Predicted compared to observed felodipine and dehydrofelodipine plasma concentration-time profiles (semi-logarithmic) after intravenous and oral administration of felodipine. Observed data are shown as dots and triangles ± standard deviation; model predictions are shown as solid lines. Details on dosing regimens, study populations and literature references are listed in Table S1. bid: twice daily, HT: hypertension, iv: intravenous, md: multiple dose, n: number of individuals, qd: once daily, RI: renal impairment, sol: solution, sd: single dose, tab: tablet, tabER: extended release tablet. *(continued)*

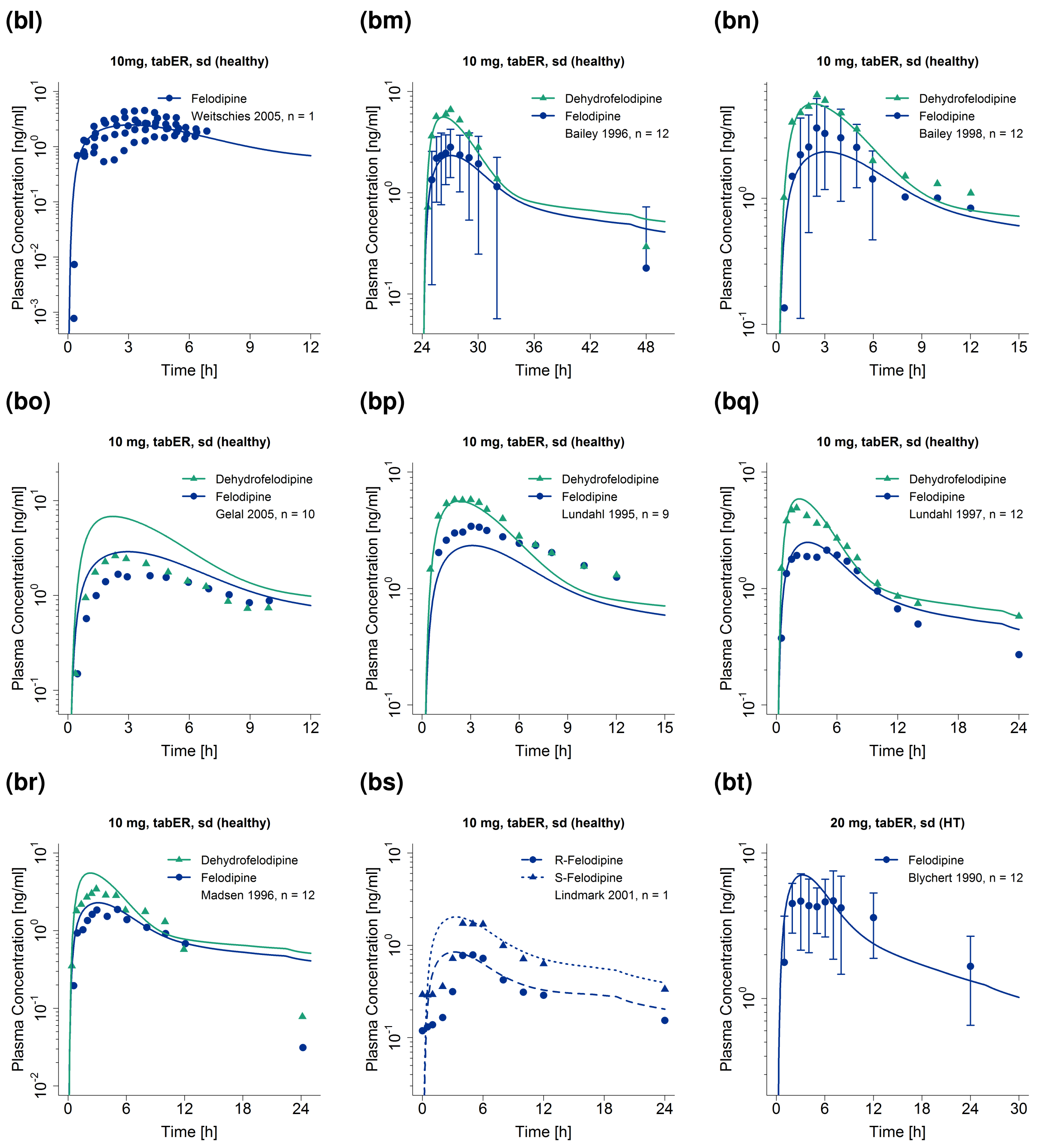

Figure S3: Predicted compared to observed felodipine and dehydrofelodipine plasma concentration-time profiles (semi-logarithmic) after intravenous and oral administration of felodipine. Observed data are shown as dots and triangles ± standard deviation; model predictions are shown as solid lines. Details on dosing regimens, study populations and literature references are listed in Table S1. bid: twice daily, HT: hypertension, iv: intravenous, md: multiple dose, n: number of individuals, qd: once daily, RI: renal impairment, sol: solution, sd: single dose, tab: tablet, tabER: extended release tablet. *(continued)*

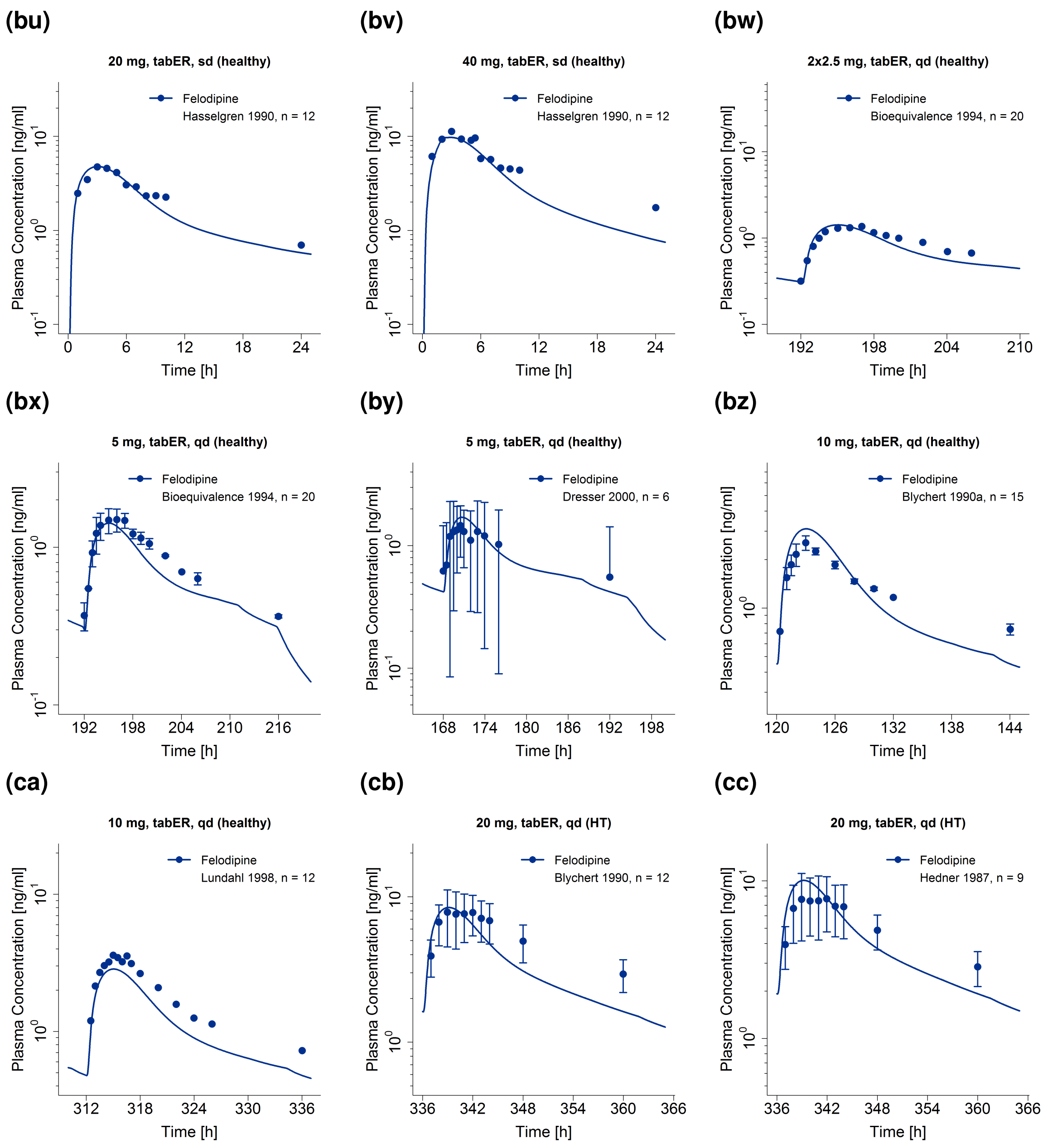

Figure S3: Predicted compared to observed felodipine and dehydrofelodipine plasma concentration-time profiles (semi-logarithmic) after intravenous and oral administration of felodipine. Observed data are shown as dots and triangles ± standard deviation; model predictions are shown as solid lines. Details on dosing regimens, study populations and literature references are listed in Table S1. bid: twice daily, HT: hypertension, iv: intravenous, md: multiple dose, n: number of individuals, qd: once daily, RI: renal impairment, sol: solution, sd: single dose, tab: tablet, tabER: extended release tablet. *(continued)*

2.4.3 Dissolution-time profile

(a)

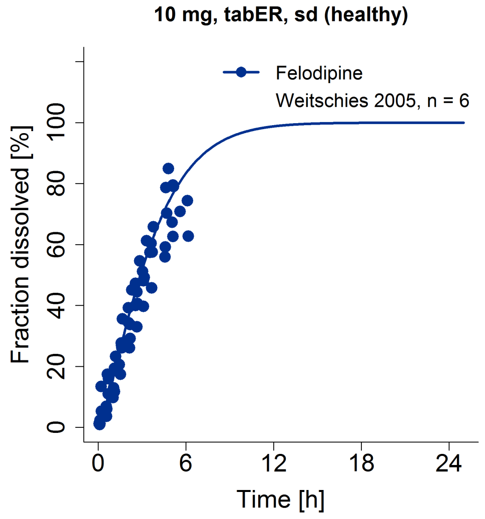

Figure S4: Predicted compared to observed dissolution-time profiles after administration of 10 mg felodipine as extended release tablet. Observed data are shown as dots; the model prediction is shown as solid line. Details on dosing regimen, study population and literature reference are listed in Table S1. n: number of individuals, sd: single dose, tabER: extended release tablet

2.5 Model evaluation

2.5.1 Plasma concentration goodness-of-fit plots

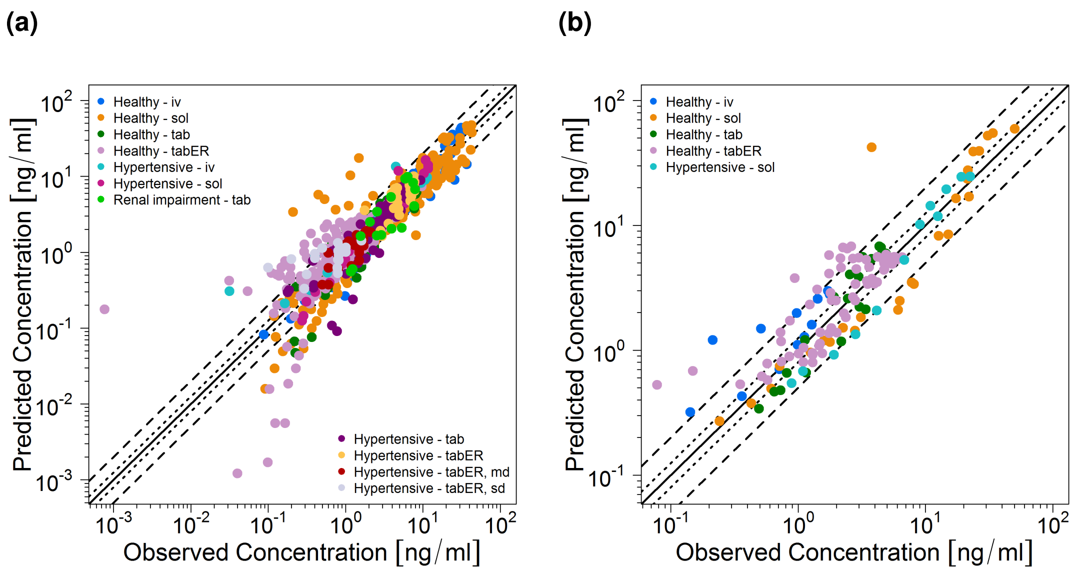

Figure S5: Predicted compared to observed plasma concentrations of (a) felodipine and (b) dehydrofelodipine. The solid line marks the line of identity. Dotted lines indicate 1.25-fold, dashed lines indicate 2-fold deviation. iv: intravenous, sol: solution, tab: tablet, tabER: extended release tablet.

2.5.2 Mean relative deviation of predicted plasma concentrations

Table S3: Mean relative deviation values of predicted plasma concentrations of felodipine and dehydrofelodipine

Compound	Felodipine route	Felodipine dose [mg]	MRD	Reference
Felodipine	iv (20 min)	1	1.23	Bengtsson 1988 [6]
Felodipine	iv (20 min)	1	1.82	Edgar 1987 [7]
Felodipine	iv (15 min)	1	1.39	Edgar 1989 [49]
Felodipine	iv (30 min)	1	1.32	Sutfin 1990 [8]
Felodipine	iv (150 min)	1.5	1.19	Sluiter 1985 [9]
Felodipine	iv (60 min)	1.5	1.26	Lundahl 1997 [10]
Dehydrofelodipine	iv (60 min)	1.5	2.06	Lundahl 1997 [10]
Felodipine	iv (105 - 120 min)	2.25	1.89	Blychert 1990 [50]
Felodipine	iv (30 min)	2.5	1.71	Edgar 1985 [11]
Felodipine	iv (30 min)	2.5	1.26	Edgar 1985a [12]
Felodipine	iv (20 min)	3	1.24	Bengtsson 1988 [6]
Felodipine	iv (20 min)	3	1.66	Edgar 1987 [7]
Felodipine	po (sol), sd	0.83	1.67	Edgar 1985 [11]
Felodipine	po (sol), sd	5	1.29	Bengtsson 1988 [6]
Felodipine	po (sol), sd	5	1.83	Edgar 1987 [7]
Felodipine	po (sol), sd	8.3	1.19	Edgar 1985 [11]
Felodipine	po (sol), sd	10	1.67	Abrahamsson 1994 [13]
Felodipine	po (sol), sd	10	1.14	Bengtsson 1990 [14]
Felodipine	po (sol), sd	10	1.57	Edgar 1987a [15]
Felodipine	po (sol), sd	10	1.28	Wingstrand 1990 [16]
Felodipine	po (sol), sd	10	1.36	Edgar 1989 [49]
Dehydrofelodipine	po (sol), sd	10	1.56	Edgar 1989 [49]
Felodipine	po (sol), sd	10.35	2.49	Johnsson 1983 [18]
Felodipine	po (sol), sd	15	1.15	Bengtsson 1988 [6]
Felodipine	po (sol), sd	15	1.42	Edgar 1987 [7]
Felodipine	po (sol), sd	20	1.72	Soons 1990 [4]
Dehydrofelodipine	po (sol), sd	20	1.74	Soons 1990 [4]
R-Felodipine	po (sol), sd	20	2.26	Soons 1990 [4]
S-Felodipine	po (sol), sd	20	1.89	Soons 1990 [4]
Felodipine	po (sol), sd	20	2.67	Soons 1993 [19]
Dehydrofelodipine	po (sol), sd	20	2.15	Soons 1993 [19]
R-Felodipine	po (sol), sd	20	2.69	Soons 1993 [19]
S-Felodipine	po (sol), sd	20	1.88	Soons 1993 [19]
Felodipine	po (sol), sd	27.5	1.57	Edgar 1985 [11]
Felodipine	po (sol), sd	27.5	1.93	Edgar 1985a [12]
Felodipine	po (sol), sd	40	1.13	Bengtsson 1988 [6]
Felodipine	po (sol), sd	40	1.20	Edgar 1987 [7]
Felodipine	po (sol), md	10	1.67	Blychert 1990a [17]
Felodipine	po (tab), sd	5	1.19	Bailey 1993 [20]
Dehydrofelodipine	po (tab), sd	5	1.51	Bailey 1993 [20]
Felodipine	po (tab), sd	5	1.12	Edgar 1992 [21]
Dehydrofelodipine	po (tab), sd	5	1.43	Edgar 1992 [21]
Felodipine	po (tab), sd	10	1.34	Edgar 1987a [15]
Felodipine	po (tab), sd	10	1.23	Guo 2007 [24]
Felodipine	po (tab), sd	10	1.31	Hardy 1988 [26]
Felodipine	po (tab), sd	10	2.03	Larsson 1990 [51]
Felodipine	po (tab), sd	10	1.90	Larsson 1990 [51]
Felodipine	po (tab), bid	5	2.07	Landahl 1988 [22]
Felodipine	po (tab), bid	5	1.39	Landahl 1988 [22]
Felodipine	po (tab), bid	10	1.67	Blychert 1990a [17]
Felodipine	po (tab), qd	10	1.70	Blychert 1990a [17]

bid: twice daily, iv: intravenous, MRD: mean relative deviation, po: oral, qd: once daily, sd: single dose, sol: solution, tab: tablet, tabER: extended release tablet

Table S3: Mean relative deviation values of predicted plasma concentrations of felodipine and dehydrofelodipine *(continued)*

Compound	Felodipine route	Felodipine dose [mg]	MRD	Reference
Felodipine	po (tab), bid	10	1.81	Hedner 1986 [54]
Felodipine	po (tab), bid	10	1.28	Hedner 1987 [52]
Felodipine	po (tab), bid	10	1.41	Larsson 1990 [51]
Felodipine	po (tab), bid	10	1.67	Larsson 1990 [51]
Felodipine	po (tab), bid	10	1.58	Smith 1987 [48]
Felodipine	po (tabER), sd	5	2.82	Aguilar-Carrasco 2015 [29]
Felodipine	po (tabER), sd	5	2.13	Dresser 2000 [27]
Felodipine	po (tabER), sd	5	1.60	Goosen 2004 [30]
Felodipine	po (tabER), sd	5	1.24	Jalava 1997 [28]
Felodipine	po (tabER), sd	5	4.39	Xiang 2017 [31]
Felodipine	po (tabER), sd	5	1.87	Leenen 2010 [53]
Felodipine	po (tabER), sd	5	2.10	Leenen 2010 [53]
Felodipine	po (tabER), sd	10	1.64	Bailey 1995 [33]
Felodipine	po (tabER), sd	10	2.20	Bailey 2000 [37]
Felodipine	po (tabER), sd	10	1.91	Bailey 2003 [38]
Felodipine	po (tabER), sd	10	1.29	Dresser 2017 [39]
Felodipine	po (tabER), sd	10	1.35	Edgar 1987a [15]
Felodipine	po (tabER), sd	10	1.29	Hasselgren 1990 [40]
Felodipine	po (tabER), sd	10	1.48	Lown 1997 [41]
Felodipine	po (tabER), sd	10	1.13	Lundahl 1998 [43]
Felodipine	po (tabER), sd	10	2.61	Patel 2011 [47]
Felodipine	po (tabER), sd	10	1.78	Pop 2008 [44]
Felodipine	po (tabER), sd	10	5.36	Weitschies 2005 [46]
Felodipine	po (tabER), sd	10	1.36	Bailey 1996 [35]
Felodipine	po (tabER), sd	10	1.57	Bailey 1998 [36]
Dehydrofelodipine	po (tabER), sd	10	1.18	Bailey 1998 [36]
Felodipine	po (tabER), sd	10	1.93	Gelal 2005 [45]
Dehydrofelodipine	po (tabER), sd	10	2.56	Gelal 2005 [45]
Felodipine	po (tabER), sd	10	1.55	Lundahl 1995 [42]
Dehydrofelodipine	po (tabER), sd	10	1.29	Lundahl1995 [42]
Felodipine	po (tabER), sd	10	1.25	Lundahl 1997 [10]
Dehydrofelodipine	po (tabER), sd	10	1.17	Lundahl 1997 [10]
Felodipine	po (tabER), sd	10	2.30	Madsen 1996 [34]

bid: twice daily, iv: intravenous, MRD: mean relative deviation, po: oral, qd: once daily, sd: single dose, sol: solution, tab: tablet, tabER: extended release tablet

Table S3: Mean relative deviation values of predicted plasma concentrations of felodipine and dehydrofelodipine *(continued)*

Compound	Felodipine route	Felodipine dose [mg]	MRD	Reference
Dehydrofelodipine	po (tabER), sd	10	2.00	Madsen 1996 [34]
R-Felodipine	po (tabER), sd	10	2.00	Lindmark 2002 [25]
S-Felodipine	po (tabER), sd	10	2.01	Lindmark 2002 [25]
Felodipine	po (tabER), sd	20	1.44	Blychert 1990 [50]
Felodipine	po (tabER), sd	20	1.19	Hasselgren 1990 [40]
Felodipine	po (tabER), sd	40	1.35	Hasselgren 1990 [40]
Felodipine	po (tabER), qd	5	1.19	Bioequivalence 1994 [32]
Felodipine	po (tabER), qd	5	1.20	Bioequivalence 1994 [32]
Felodipine	po (tabER), qd	5	1.23	Dresser 2000 [27]
Felodipine	po - tabER, md	5	1.25	Leenen 2010 [53]
Felodipine	po - tabER, md	5	1.48	Leenen 2010 [53]
Felodipine	po (tabER), qd	10	1.30	Blychert 1990a [17]
Felodipine	po (tabER), qd	10	1.31	Lundahl 1998 [43]
Felodipine	po (tabER), qd	20	1.35	Blychert 1990 [50]
Felodipine	po (tabER), qd	20	1.30	Hedner 1987 [52]
**MRD (Felodipine)**			**1.64 (1.12-5.36)**	
**MRD (Dehydrofelodipine)**			**1.70 (1.17-2.56)**	
**MRD (Enantiomers)**			**2.12 (1.88-2.69)**	
**MRD (total)**			**1.67 (1.12-5.36)**	
			**79/99 with MRD < 2**	

bid: twice daily, iv: intravenous, MRD: mean relative deviation, po: oral, qd: once daily, sd: single dose, sol: solution, tab: tablet, tabER: extended release tablet

2.5.3 AUC_{last} and C_{max} goodness-of-fit plots

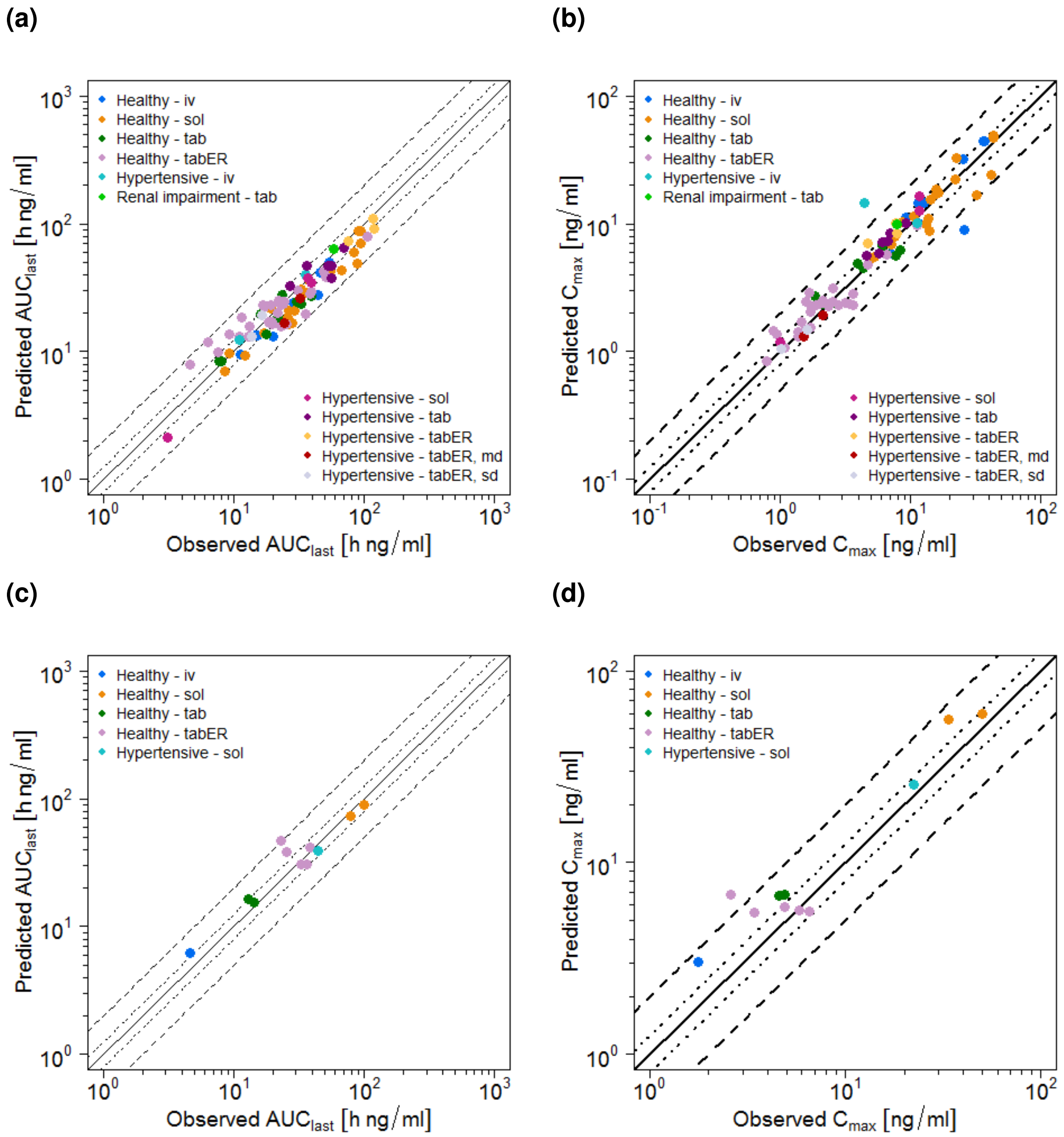

Figure S6: Predicted compared to observed AUC_{last} and C_{max} values of (a,b) felodipine and (c,d) dehydrofelodipine. The solid line marks the line of identity. Dotted lines indicate 1.25-fold, dashed lines indicate 2-fold deviation. AUC_{last}: area under the plasma concentration-time curve from the time of drug administration to the last concentration measurement, C_{max}: maximum plasma concentration, iv: intravenous, sol: solution, tab: tablet, tabER: extended release tablet.

2.5.4 Geometric mean fold error of predicted AUC_{last} and C_{max} values

Table S4: Predicted and observed AUC_{last} and C_{max} values with geometric mean fold errors of felodipine and dehydrofelodipine

			**AUC_{last} $[\frac{h \cdot ng}{ml}]$**			**C_{max} $\frac{ng}{ml}$**			
**Compound**	**Felodipine route**	**Felodipine dose**	**Pred**	**Obs**	**Pred/Obs**	**Pred**	**Obs**	**Pred/Obs**	**Reference**
Felodipine	iv (20 min)	1	13.51	14.68	0.92	14.69	11.53	1.27	Bengtsson 1988 [6]
Felodipine	iv (20 min)	1	13.24	20.09	0.66	14.54	12.53	1.16	Edgar 1987 [7]
Felodipine	iv (15 min)	1	12.25	11.14	1.10	14.46	4.46	3.25	Edgar 1989 [49]
Felodipine	iv (30 min)	1	13.41	15.05	0.89	11.10	9.16	1.21	Sutfin 1990 [8]
Felodipine	iv (150 min)	1.5	9.49	11.34	0.84	5.88	6.97	0.84	Sluiter 1985 [9]
Felodipine	iv (60 min)	1.5	24.01	28.34	0.85	10.12	11.52	0.88	Lundahl 1997 [10]
Dehydrofelodipine	iv (60 min)	1.5	6.17	4.57	1.35	3.04	1.77	1.72	Lundahl 1997 [10]
Felodipine	iv (105 - 120 min)	2.25	39.95	35.79	1.12	10.03	11.33	0.89	Blychert 1990 [50]
Felodipine	iv (30 min)	2.5	27.73	44.48	0.62	8.97	25.61	0.35	Edgar 1985 [11]
Felodipine	iv (30 min)	2.5	49.76	53.54	0.93	31.78	25.45	1.25	Edgar 1985a [12]
Felodipine	iv (20 min)	3	41.18	46.19	0.89	44.16	35.89	1.23	Bengtsson 1988 [6]
Felodipine	iv (20 min)	3	39.02	51.62	0.76	43.77	36.93	1.19	Edgar 1987 [7]
Felodipine	po (sol), sd	0.83	2.12	3.07	0.69	1.19	1.00	1.19	Edgar 1985 [11]
Felodipine	po (sol), sd	5	9.70	9.16	1.06	5.37	5.13	1.05	Bengtsson 1988 [6]
Felodipine	po (sol), sd	5	9.31	12.25	0.76	5.55	5.29	1.05	Edgar 1987 [7]
Felodipine	po (sol), sd	8.3	34.82	39.55	0.88	12.72	11.75	1.08	Edgar 1985 [11]
Felodipine	po (sol), sd	10	16.50	26.15	0.63	9.87	13.20	0.75	Abrahamsson 1994[13]
Felodipine	po (sol), sd	10	7.05	8.52	0.83	6.88	7.18	0.96	Bengtsson 1990 [14]
Felodipine	po (sol), sd	10	18.61	25.76	0.72	10.30	8.63	1.19	Edgar 1987a [15]
Felodipine	po (sol), sd	10	20.96	26.53	0.79	10.95	13.57	0.81	Wingstrand 1990[16]
Felodipine	po (sol), sd	10	37.62	36.65	1.03	16.41	11.75	1.40	Edgar 1989 [49]
Dehydrofelodipine	po (sol), sd	10	38.85	44.19	0.88	25.54	22.48	1.14	Edgar 1989 [49]
Felodipine	po (sol), sd	10.35	21.87	19.14	1.14	18.62	15.73	1.18	Johnsson 1983[18]
Felodipine	po (sol), sd	15	30.40	32.90	0.92	17.13	15.65	1.09	Bengtsson 1988[6]
Felodipine	po (sol), sd	15	28.63	35.30	0.81	17.50	16.43	1.07	Edgar 1987 [7]
Felodipine	po (sol), sd	20	42.08	55.35	0.76	22.07	22.11	1.00	Soons 1990 [4]
Dehydrofelodipine	po (sol), sd	20	72.60	79.22	0.92	55.48	33.80	1.64	Soons 1990 [4]
R-Felodipine	po (sol), sd	20	14.07	17.10	0.82	8.00	7.55	1.06	Soons 1990 [4]
S-Felodipine	po (sol), sd	20	35.73	37.97	0.94	15.42	14.17	1.09	Soons 1990 [4]
Felodipine	po (sol), sd	20	48.79	88.45	0.55	24.34	41.70	0.58	Soons 1993 [19]

AUC_{last}: area under the plasma concentration-time curve calculated from the time of drug administration to the time of the last concentration measurement, bid: twice daily, C_{max}: maximum plasma concentration, GMFE: geometric mean fold error, iv: intravenous, obs: observed, pred: predicted, po: oral, qd: once daily, sd: single dose, sol: solution, tab: tablet, tabER: extended release tablet

Table S4: Predicted and observed AUC_{last} and C_{max} values with geometric mean fold errors of felodipine and dehydrofelodipine *(continued)*

			AUC_{last} $[\frac{h \cdot ng}{ml}]$			C_{max} $\frac{ng}{ml}$			
**Compound**	**Felodipine route**	**Felodipine dose**	**Pred**	**Obs**	**Pred/Obs**	**Pred**	**Obs**	**Pred/Obs**	**Reference**
Dehydrofelodipine	po (sol), sd	20	89.59	98.76	0.91	59.32	50.36	1.18	Soons 1993 [19]
R-Felodipine	po (sol), sd	20	16.60	28.01	0.59	8.83	14.08	0.63	Soons 1993 [19]
S-Felodipine	po (sol), sd	20	43.67	67.13	0.65	16.84	32.21	0.52	Soons 1993 [19]
Felodipine	po (sol), sd	27.5	60.32	83.46	0.72	32.70	22.31	1.47	Edgar 1985 [11]
Felodipine	po (sol), sd	27.5	70.19	93.20	0.75	32.70	22.59	1.45	Edgar 1985a [12]
Felodipine	po (sol), sd	40	86.91	90.82	0.96	46.99	43.21	1.09	Bengtsson 1988 [6]
Felodipine	po (sol), sd	40	87.16	93.48	0.93	48.90	42.79	1.14	Edgar 1987 [7]
Felodipine	po (sol), md	10	20.75	29.35	0.71	11.52	10.63	1.08	Blychert 1990a [17]
Felodipine	po (tab), sd	5	8.46	7.97	1.06	2.34	2.46	0.95	Bailey 1993 [20]
Dehydrofelodipine	po (tab), sd	5	15.58	14.24	1.09	6.72	4.59	1.47	Bailey 1993 [20]
Felodipine	po (tab), sd	5	8.47	7.75	1.09	2.35	2.11	1.11	Edgar 1992 [21]
Dehydrofelodipine	po (tab), sd	5	16.54	12.95	1.28	6.83	4.89	1.40	Edgar 1992 [21]
Felodipine	po (tab), sd	10	19.67	16.14	1.22	4.87	3.94	1.24	Edgar 1987a [15]
Felodipine	po (tab), sd	10	27.77	23.67	1.17	6.78	6.13	1.11	Guo 2007 [24]
Felodipine	po (tab), sd	10	18.60	21.76	0.85	4.54	4.35	1.04	Hardy 1988 [26]
Felodipine	po (tab), sd	10	32.63	26.75	1.22	5.91	5.70	1.04	Larsson 1990 [51]
Felodipine	po (tab), sd	10	46.50	36.14	1.29	8.43	6.97	1.21	Larsson 1990 [51]
Felodipine	po (tab), bid	5	13.65	17.69	0.77	2.72	1.84	1.48	Landahl 1988 [22]
Felodipine	po (tab), bid	5	46.45	56.27	0.83	5.67	4.64	1.22	Landahl 1988 [22]
Felodipine	po (tab), bid	10	23.51	33.07	0.71	5.91	6.45	0.92	Blychert 1990a [17]
Felodipine	po (tab), qd	10	24.48	30.50	0.80	5.60	7.72	0.73	Blychert 1990a [17]
Felodipine	po (tab), bid	10	37.86	55.95	0.68	7.28	6.73	1.08	Hedner 1986 [54]
Felodipine	po (tab), bid	10	65.28	69.52	0.94	10.07	9.29	1.08	Hedner 1987 [52]
Felodipine	po (tab), bid	10	47.19	52.40	0.90	7.10	6.09	1.17	Larsson 1990 [51]
Felodipine	po (tab), bid	10	63.28	58.02	1.09	9.86	7.93	1.24	Larsson 1990 [51]
Felodipine	po (tab), bid	10	27.20	39.09	0.70	6.24	8.30	0.75	Smith 1987 [48]
Felodipine	po (tabER), sd	5	15.68	22.89	0.68	1.32	1.37	0.97	Aguilar-Carrasco 2015 [29]
Felodipine	po (tabER), sd	5	7.92	4.65	1.70	1.36	0.95	1.44	Dresser 2000 [27]
Felodipine	po (tabER), sd	5	15.79	13.10	1.21	1.46	0.89	1.65	Goosen 2004 [30]
Felodipine	po (tabER), sd	5	16.30	19.76	0.82	1.07	1.07	0.99	Jalava 1997 [28]
Felodipine	po (tabER), sd	5	19.48	35.60	0.55	1.54	1.70	0.90	Xiang 2017 [31]
Felodipine	po (tabER), md	5	19.26	16.31	1.18	1.47	1.61	0.91	Leenen 2010 [53]
Felodipine	po (tabER), md	5	13.15	13.86	0.95	1.04	1.01	1.03	Leenen 2010 [53]

AUC_{last}: area under the plasma concentration-time curve calculated from the time of drug administration to the time of the last concentration measurement, bid: twice daily, C_{max}: maximum plasma concentration, GMFE: geometric mean fold error, iv: intravenous, obs: observed, pred: predicted, po: oral, qd: once daily, sd: single dose, sol: solution, tab: tablet, tabER: extended release tablet

Table S4: Predicted and observed AUC_{last} and C_{max} values with geometric mean fold errors of felodipine and dehydrofelodipine *(continued)*

			AUC_{last} $[\frac{h \cdot ng}{ml}]$			C_{max} $\frac{ng}{ml}$			
**Compound**	**Felodipine route**	**Felodipine dose**	**Pred**	**Obs**	**Pred/Obs**	**Pred**	**Obs**	**Pred/Obs**	**Reference**
Felodipine	po (tabER), sd	10	23.72	23.46	1.01	2.33	2.83	0.82	Bailey 1995 [33]
Felodipine	po (tabER), sd	10	13.78	9.24	1.49	2.38	1.98	1.20	Bailey 2000 [37]
Felodipine	po (tabER), sd	10	13.05	11.06	1.18	2.25	2.34	0.96	Bailey 2003 [38]
Felodipine	po (tabER), sd	10	13.19	12.91	1.02	2.28	2.34	0.97	Dresser 2017 [39]
Felodipine	po (tabER), sd	10	17.04	18.51	0.92	2.39	2.48	0.96	Edgar 1987a [15]
Felodipine	po (tabER), sd	10	23.59	23.83	0.99	2.39	2.10	1.14	Hasselgren 1990 [40]
Felodipine	po (tabER), sd	10	22.99	19.15	1.20	2.27	1.75	1.30	Lown 1997 [41]
Felodipine	po (tabER), sd	10	24.56	24.43	1.01	2.43	2.53	0.96	Lundahl 1998 [43]
Felodipine	po (tabER), sd	10	38.26	50.33	0.76	5.74	6.57	0.87	Patel 2011 [47]
Felodipine	po (tabER), sd	10	28.43	38.21	0.74	2.39	3.17	0.75	Pop 2008 [44]
Felodipine	po (tabER), sd	10	11.90	6.33	1.88	2.48	1.58	1.57	Weitschies 2005 [46]
Felodipine	po (tabER), sd	10	23.20	22.78	1.02	2.33	2.82	0.83	Bailey 1996 [35]
Dehydrofelodipine	po (tabER), sd	10	38.76	40.97	0.95	5.59	6.59	0.85	Bailey 1996 [35]
Felodipine	po (tabER), sd	10	17.06	19.78	0.86	2.33	3.59	0.65	Bailey 1998 [36]
Dehydrofelodipine	po (tabER), sd	10	30.83	32.78	0.94	5.59	6.56	0.85	Bailey 1998 [36]
Felodipine	po (tabER), sd	10	18.64	11.59	1.61	2.89	1.67	1.73	Gelal 2005 [45]
Dehydrofelodipine	po (tabER), sd	10	47.05	22.95	2.05	6.81	2.61	2.61	Gelal 2005 [45]
Felodipine	po (tabER), sd	10	16.54	25.53	0.65	2.34	3.42	0.68	Lundahl 1995 [42]
Dehydrofelodipine	po (tabER), sd	10	30.97	36.52	0.85	5.63	5.77	0.98	Lundahl1995 [42]
Felodipine	po (tabER), sd	10	25.10	22.14	1.13	2.49	2.13	1.17	Lundahl 1997 [10]
Dehydrofelodipine	po (tabER), sd	10	41.50	38.38	1.08	5.88	4.91	1.20	Lundahl 1997 [10]
Felodipine	po (tabER), sd	10	23.19	16.72	1.39	2.29	1.88	1.22	Madsen 1996 [34]
Dehydrofelodipine	po (tabER), sd	10	38.31	25.63	1.49	5.52	3.43	1.61	Madsen 1996 [34]
R-Felodipine	po (tabER), sd	10	9.87	7.62	1.30	0.83	0.79	1.06	Lindmark 2002 [25]
S-Felodipine	po (tabER), sd	10	22.02	17.03	1.29	1.99	1.72	1.16	Lindmark 2002 [25]
Felodipine	po (tabER), sd	20	74.06	75.87	0.98	7.07	4.70	1.50	Blychert 1990 [50]
Felodipine	po (tabER), sd	20	42.22	48.54	0.87	4.78	4.73	1.01	Hasselgren 1990 [40]
Felodipine	po (tabER), sd	40	79.78	105.95	0.75	9.73	11.27	0.86	Hasselgren 1990 [40]
Felodipine	po (tabER), qd	5	16.58	18.68	0.89	1.42	1.37	1.04	Bioequivalence 1994 [32]
Felodipine	po (tabER), qd	5	16.59	19.52	0.85	1.42	1.50	0.95	Bioequivalence 1994 [32]
Felodipine	po (tabER), qd	5	19.88	21.58	0.92	1.71	1.46	1.17	Dresser 2000 [27]
Felodipine	po (tabER), md	5	25.88	32.24	0.80	1.92	2.11	0.91	Leenen 2010[53]
Felodipine	po (tabER), md	5	16.83	24.46	0.69	1.31	1.50	0.87	Leenen 2010[53]

AUC_{last}: area under the plasma concentration-time curve calculated from the time of drug administration to the time of the last concentration measurement, bid: twice daily, C_{max}: maximum plasma concentration, GMFE: geometric mean fold error, iv: intravenous, obs: observed, pred: predicted, po: oral, qd: once daily, sd: single dose, sol: solution, tab: tablet, tabER: extended release tablet

Table S4: Predicted and observed AUC_{last} and C_{max} values with geometric mean fold errors of felodipine and dehydrofelodipine *(continued)*

			AUC_{last} $[\frac{h \cdot ng}{ml}]$			C_{max} $\frac{ng}{ml}$			
**Compound**	**Felodipine route**	**Felodipine dose**	**Pred**	**Obs**	**Pred/Obs**	**Pred**	**Obs**	**Pred/Obs**	**Reference**
Felodipine	po (tabER), qd	10	30.28	31.24	0.97	3.11	2.55	1.22	Blychert 1990a [17]
Felodipine	po (tabER), qd	10	29.21	39.09	0.75	2.85	3.59	0.79	Lundahl 1998 [43]
Felodipine	po (tabER), qd	20	90.55	119.93	0.75	8.44	7.84	1.08	Blychert 1990 [50]
Felodipine	po (tabER), qd	20	108.61	117.57	0.92	10.06	7.69	1.31	Hedner 1987 [52]
**GMFE (Felodipine)**					**1.25 (1.01-1.88)**			**1.25 (1.00-3.25)**	
**GMFE (Dehydrofelodipine)**					**1.25 (1.06-2.05)**			**1.44 (1.03-2.61)**	
**GMFE (Enantiomers)**					**1.35 (1.06-1.69)**			**1.32 (1.06-1.91)**	
**GMFE (total)**					**1.26 (1.01-2.05)**			**1.28 (1.00-3.25)**	
					**99/100 with GMFE < 2**			**97/100 with GMFE < 2**	

AUC_{last}: area under the plasma concentration-time curve calculated from the time of drug administration to the time of the last concentration measurement, bid: twice daily, C_{max}: maximum plasma concentration, GMFE: geometric mean fold error, iv: intravenous, obs: observed, pred: predicted, po: oral, qd: once daily, sd: single dose, sol: solution, tab: tablet, tabER: extended release tablet

2.5.5 Sensitivity analysis

Sensitivity of the felodipine PBPK model to single parameters (local sensitivity analysis) was calculated as the relative change of the predicted felodipine and dehydrofelodipine (1) AUC from 0-24 h ($AUC_{0\text{-}24}$) after 1.5 mg of felodipine as intravenous infusion and (2) AUC at steady-state (AUC_{ss}) after 5 mg of felodipine once daily as extended release tablet. Parameters were included into the analysis if they were optimized (*Felodipine*: solubility tabER, intestinal permeability, k_{cat} CYP3A4, Weibull parameters [dissolution shape and dissolution time 50%]; *Dehydrofelodipine*: fraction unbound, lipophilicity, CL_{hep} and CL_{CYP3A4}), if they are associated with optimized parameters (*Felodipine*: K_m) or if they might have a strong impact due to calculation methods used in the model (*Felodipine*: fraction unbound, solubility, lipophilicity, GFR fraction; *Dehydrofelodipine*: GFR fraction). Results of the sensitivity analysis are illustrated in Figures S7 and S8. Sensitivity analyses of the felodipine parent-metabolite PBPK model revealed that the felodipine $AUC_{0\text{-}24}$ after intravenous administration and AUC_{ss} after oral administration are mainly sensitive to felodipine lipophilicity, fraction unbound in plasma and K_m and k_{cat} of CYP3A4. Dehydrofelodipine $AUC_{0\text{-}24}$ after intravenous administration of felodipine and AUC_{ss} after oral administration of felodipine are mainly sensitive to dehydrofelodipine fraction unbound in plasma, CYP3A4 clearance and lipophilicity.

(a)

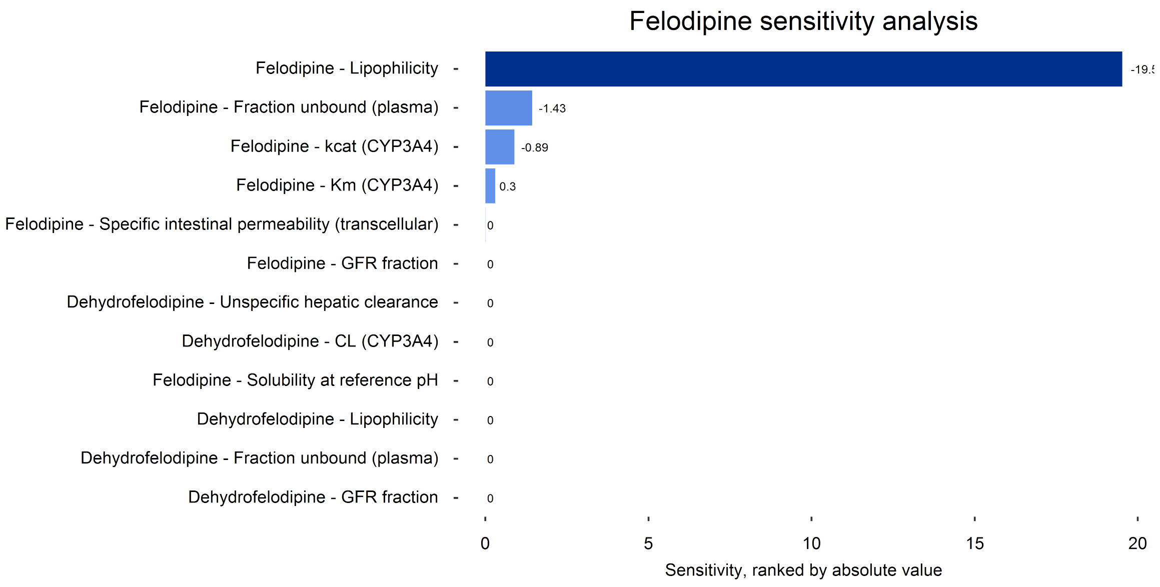

(b)

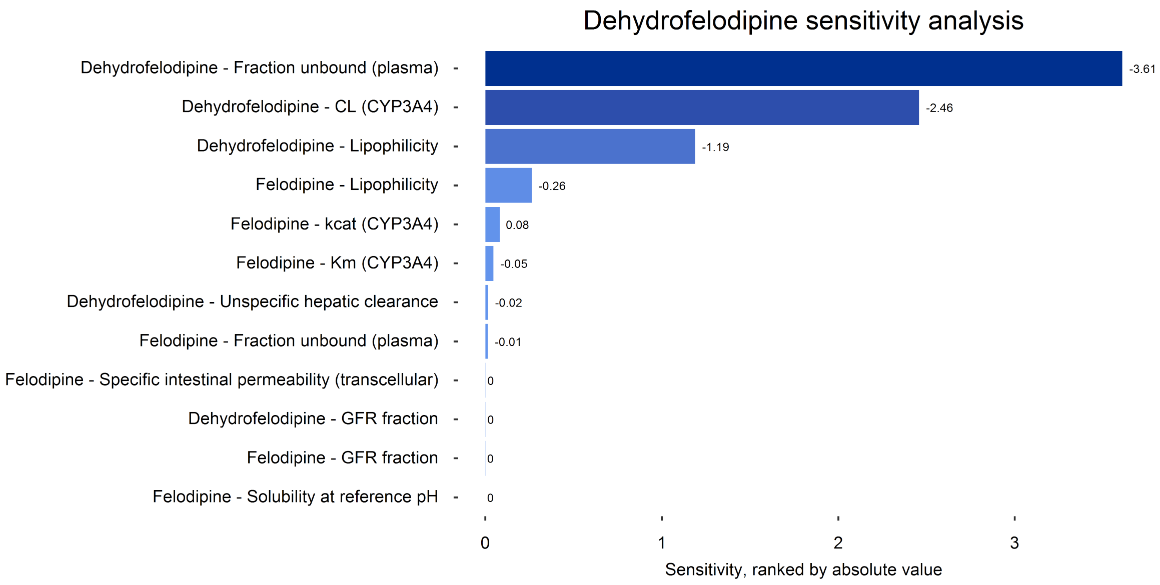

Figure S7: Felodipine parent-metabolite PBPK model sensitivity analysis for intravenous felodipine administration. Sensitivity of the felodipine PBPK model to single parameters, calculated as change of (a) the simulated felodipine $AUC_{0\text{-}24}$ and (b) the simulated dehydrofelodipine $AUC_{0\text{-}24}$ after administration of 1.5 mg felodipine as intravenous infusion. CL: clearance, CYP: cytochrome P450, GFR: glomerular filtration rate, k_{cat}: catalytic rate constant, K_m: Michaelis-Menten constant

(a)

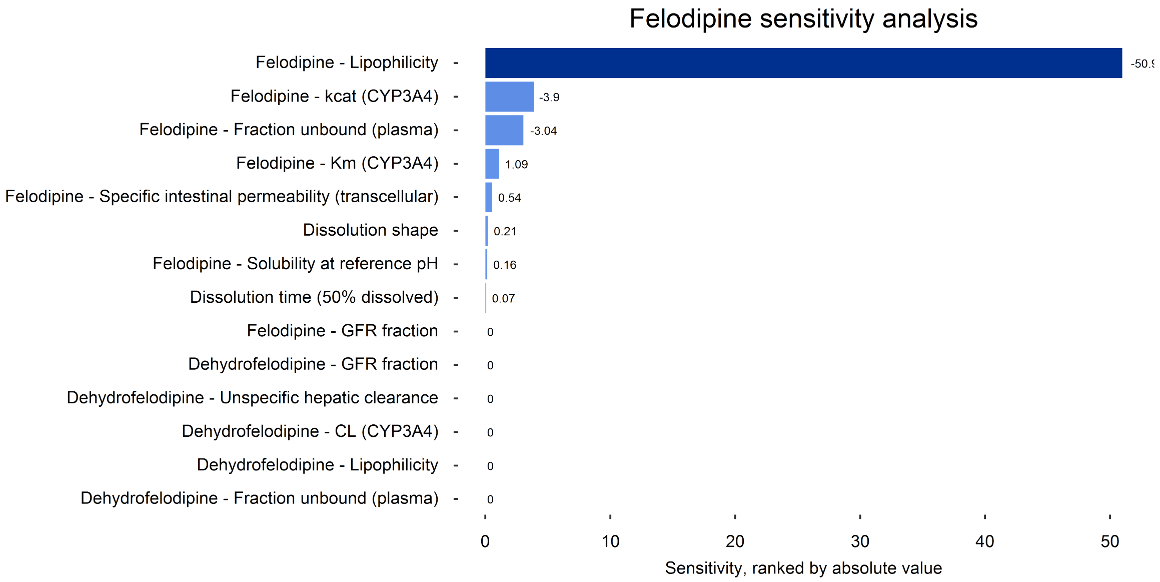

(b)

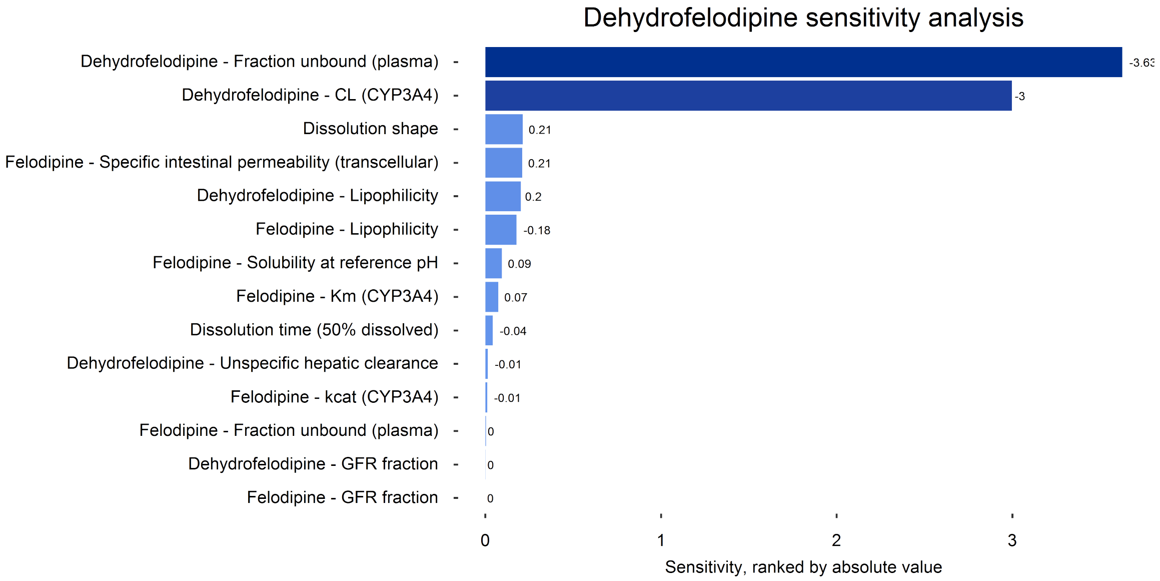

Figure S8: Felodipine parent-metabolite PBPK model sensitivity analysis for oral felodipine administration. Sensitivity of the felodipine PBPK model to single parameters, calculated as change of (a) the simulated felodipine AUC_{ss} and (b) the simulated dehydrofelodipine AUC_{ss} after administration of 5 mg of felodipine once daily as extended release tablet. CL: clearance, CYP: cytochrome P450, GFR: glomerular filtration rate, k_{cat}: catalytic rate constant, K_m: Michaelis-Menten constant

3 Felodipine - pharmacodynamic modeling

3.1 PD model building

The PD model extension describes the circadian rhythm of blood pressure and heart rate using models from Chae et al. [2] and Lott et al. [3], respectively. Circadian model parameters were optimized for each study individually, as described in Section 1. Table S5 displays optimized circadian parameters for blood pressure and heart rate for each study and indicates if placebo profiles were available. The effect of felodipine on blood pressure and heart rate was subsequently described using an E_{max} function. Parameters used in the final PD models are shown in Table S6 .

The performance of the felodipine PD model is demonstrated in linear plots of predicted compared to observed effect-time profiles in Figures S9 and S10 for blood pressure and heart rate, respectively. Goodness-of-fit plots are presented in Figure S11, corresponding MRD values are listed in Tables S7 and S8.

3.2 PD model parameters

Table S5: Optimized parameters of the circadian blood pressure and heart rate models

Dose	Route	BP_{mean}	$phase_{12,BP}$ [h]	$phase_{24,BP}$ [h]	placebo available	HR_{mean}	$phase_{HR}$	placebo available	Reference
**Healthy**									
1.5	iv (20min)	71.86	3.20	8.32	yes	62.18	17.31	yes	Sluiter 1985 [9]
5	po (sol), sd	68.54	0.89	6.01	yes	57.97	12.81	yes	Bengtsson 1988 [6]
5	po (sol), sd	69.57	0.95	6.07	yes	56.81	18.48	no	Edgar 1987 [7]
10	po (sol), sd	62.38	1.46	6.58	yes	56.06	12.47	yes	Bengtsson 1990 [14]
10.35	po (sol), sd	96.27	3.47	8.59	no	68.88	6.91	no	Johnsson 1983 [18]
15	po (sol), sd	68.54	0.89	6.01	yes	57.97	12.81	yes	Bengtsson 1988 [6]
15	po (sol), sd	69.57	0.95	6.07	yes	56.81	18.48	no	Edgar 1987 [7]
40	po (sol), sd	68.54	0.89	6.01	yes	57.97	12.81	yes	Bengtsson 1988 [6]
40	po (sol), sd	69.57	0.95	6.07	yes	56.81	18.48	no	Edgar 1987 [7]
10	po (tab), sd	-	-	-	-	79.03	6.68	no	Guo 2007
10	po (tab), sd	77.47	2.98	8.10	yes	77.46	8.65	yes	Hardy 1988 [26]
2.5; 5	po,tabER D1-D2: 2.5 mg qd; D3-8: 5 mg qd	64.78	-0.04	5.08	no	67.11	14.54	no	Dresser 2000 [27]
5	po (tabER), sd	67.42	-1.93	3.19	no	70.36	8.11	no	Jalava 1997 [28]
5	po (tabER), sd	67.42	-1.93	3.19	no	70.36	8.11	no	Jalava 1997 [28]
5	po (tabER), sd	69.5	-1.53	3.59	no	64.80	6.73	no	Dresser 2000 [27]
10	po (tabER), sd	70.81	2.18	7.3	yes	55.80	10.47	yes	Hasselgren 1990 [40]
10	po (tabER), sd	71.44	-4.42	0.70	no	62.79	0.98	no	Lundahl 1995 [42]
20	po (tabER), sd	70.81	2.18	7.3	yes	55.80	10.47	yes	Hasselgren 1990 [40]
40	po (tabER), sd	70.81	2.18	7.3	yes	55.80	10.47	yes	Hasselgren 1990 [40]
**Hypertensive**									
2.25	iv (15 min)	95.08	-0.71	4.41	yes	61.37	13.45	yes	Blychert 1990 [50]
0.83	po (sol), sd	77.50	-0.74	4.38	yes	-	-	-	Edgar 1985 [11]
8.3	po (sol), sd	77.50	-0.74	4.38	yes	-	-	-	Edgar 1985 [11]
10	po (tab), sd	88.63	-1.55	3.57	no	-	-	-	Larsson 1990 [51]
10	po (tab), sd	88.26	-1.59	3.53	no	-	-	-	Larsson 1990 [51]

assumed

- : no data available, bid: twice daily, BP_{mean}: mean blood pressure over 24 h, D: day, HR_{mean}: mean heart rate over 24 h, iv: intravenous, phase: circadian phase, po: oral, qd: once daily, sd: single dose, tab: tablet, tabER: extended release tablet

Table S5: Optimized parameters of the circadian blood pressure and heart rate models *(continued)*

Dose	Route	BP_{mean}	$phase_{12,BP}$ [h]	$phase_{24,BP}$ [h]	placebo available	HR_{mean}	$phase_{HR}$	placebo available	Reference
10	po (*tab*), D1: 10mg sd, D2-D6: 5mg bid, D7-D29: 10mg bid	88.63	-1.55	3.57	no	-	-	-	Larsson 1990 [51]
10	po (*tab*), D1: 10mg sd, D2-D6: 5mg bid, D7-D29: 10mg bid	88.26	-1.59	3.53	no	-	-	-	Larsson 1990 [51]
10	po (tab), bid	94.06	3.59	8.71	no	-	-	-	Hedner 1987 [52]
5; 10	D1-D14: po (*tab*), bid; D15-D28: po (*tab*), bid	87.70	-0.02	5.10	yes	66.13	13.63	yes	Hedner 1986 [54]
20	po (tabER), qd	97.16	2.60	7.72	no	-	-	-	Hedner 1987 [52]
20	po (tabER), sd	95.08	-0.71	4.41	yes	61.37	13.45	yes	Blychert 1990 [50]
20	po (tabER), qd	95.88	1.01	6.13	yes	60.92	15.24	yes	Blychert 1990 [50]

assumed

- : no data available, bid: twice daily, BP_{mean}: mean blood pressure over 24 h, D: day, HR_{mean}: mean heart rate over 24 h, iv: intravenous, phase: circadian phase, po: oral, qd: once daily, sd: single dose, tab: tablet, tabER: extended release tablet

Table S6: Drug-dependent parameter of the final felodipine diastolic blood pressure and heart rate PD model

Parameter	Unit	Value (model)	Value (literature)	Reference	Description
**Pharmacokinetic model**					
Diastolic blood pressure					
E_{max}	mmHg	56.18 (opt)	-	-	Maximum effect
EC_{50}	µmol/l	0.04 (opt)	-	-	Concentration for half-maximal effect
amp_{24}	%	2.14 (lit)	2.14	[2]	Amplitude for 24 h period
amp_{12}	%	5.93 (lit)	5.93	[2]	Amplitude for 12 h period
$phase_{24}$	h	individual[a]	-	-	Phase for 24 h period
$phase_{12}$	h	individual[a]	-	-	Phase for 12 h period
BP_{mean}	mmHg	individual[a]	69.5	[2]	Mean blood pressure over 24 h
Heart rate					
E_{max}	bpm	39.71 (opt)	-	-	Maximum effect
EC_{50}	µmol/l	0.05 (opt)	-	-	Concentration for half-maximal effect
h	-	1.40 (opt)	-	-	Hill coefficient
amp	%	6.3 (lit)	6.3	[3]	Amplitude
phase	h	individual$_a$	9.2	[3]	Phase
HR_{mean}	bpm	individual[a]	66.2	[3]	Mean heart rate over 24 h

lit: literature value, opt: optimized value, [a]: individual parameters for phases and BP_{mean} and HR_{mean} are listed in Table S5

3.3 Effect-time profiles

3.3.1 Diastolic blood pressure

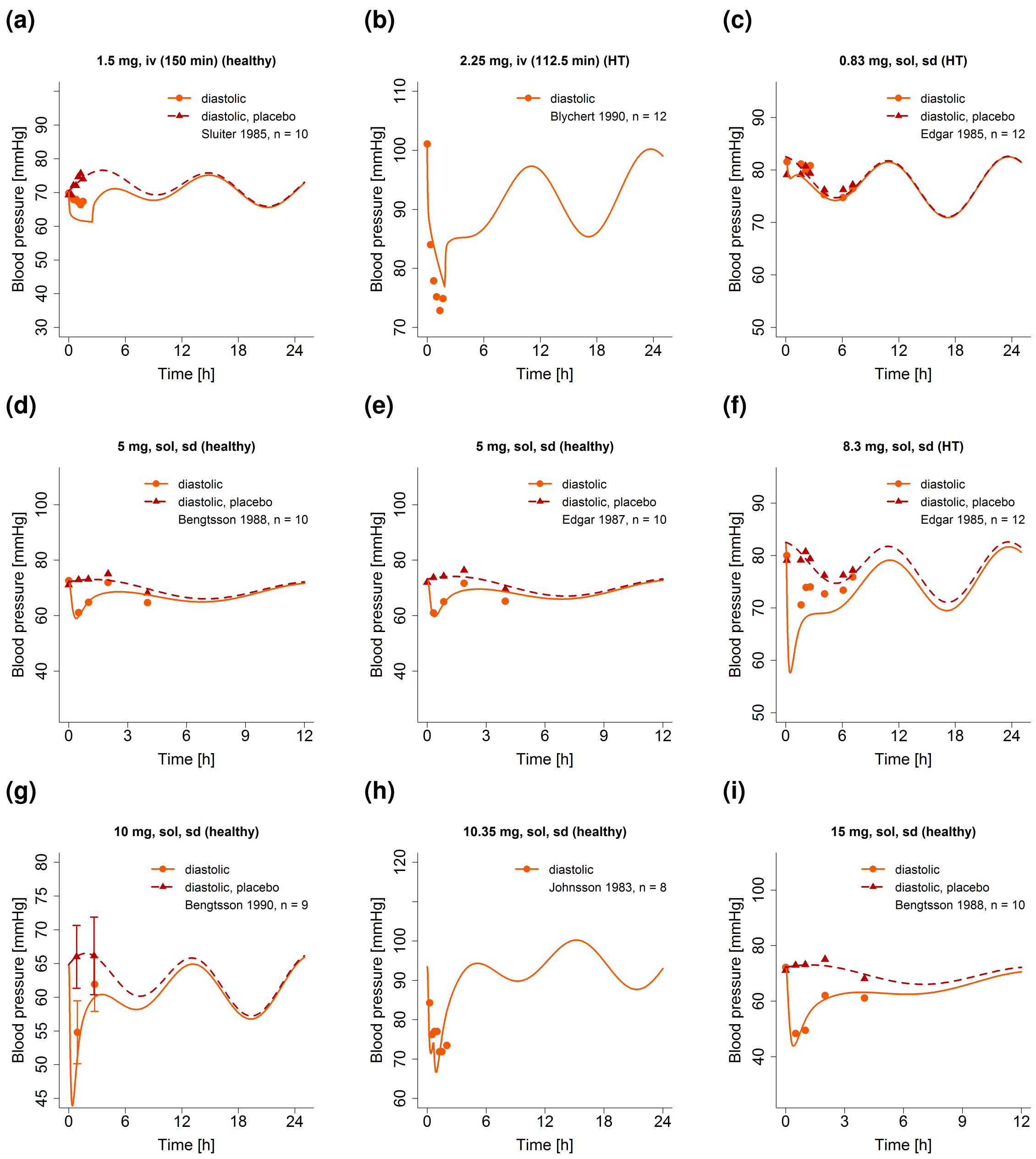

Figure S9: Predicted compared to observed effect-time profiles of diastolic blood pressure after intravenous and oral administration of felodipine. Observed data are shown as dots and triangles ± standard deviation (if available); model predictions are shown as solid lines. Details on dosing regimens, study populations and literature references are listed in Table S1. bid: twice daily, HT: hypertension, iv: intravenous, md: multiple dose, n: number of individuals, qd: once daily, RI: renal impairment, sol: solution, sd: single dose, tab: tablet, tabER: extended release tablet.

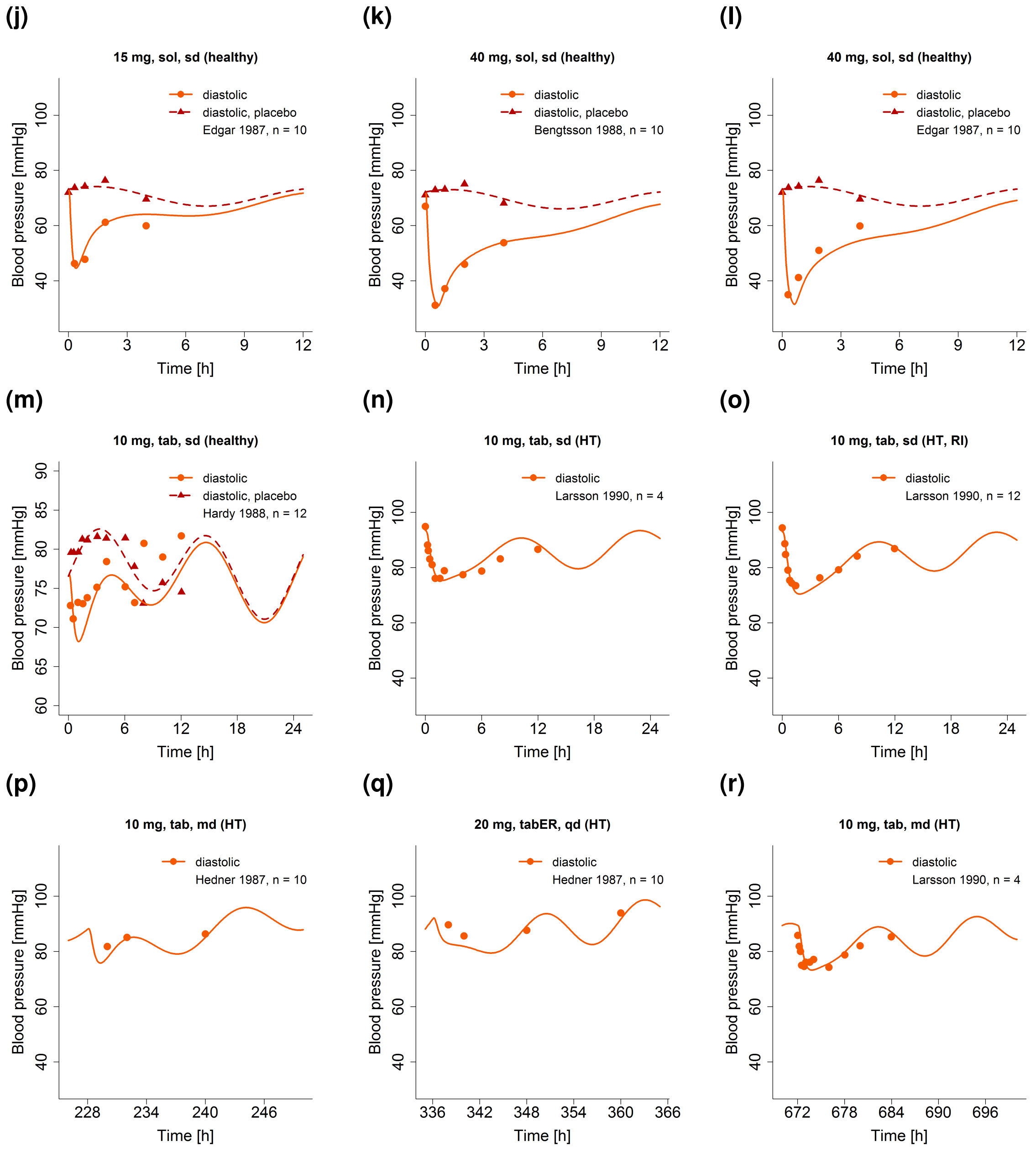

Figure S9: Predicted compared to observed effect-time profiles of diastolic blood pressure after intravenous and oral administration of felodipine. Observed data are shown as dots and triangles ± standard deviation (if available); model predictions are shown as solid lines. Details on dosing regimens, study populations and literature references are listed in Table S1. bid: twice daily, HT: hypertension, iv: intravenous, md: multiple dose, n: number of individuals, qd: once daily, RI: renal impairment, sol: solution, sd: single dose, tab: tablet, tabER: extended release tablet. *(continued)*

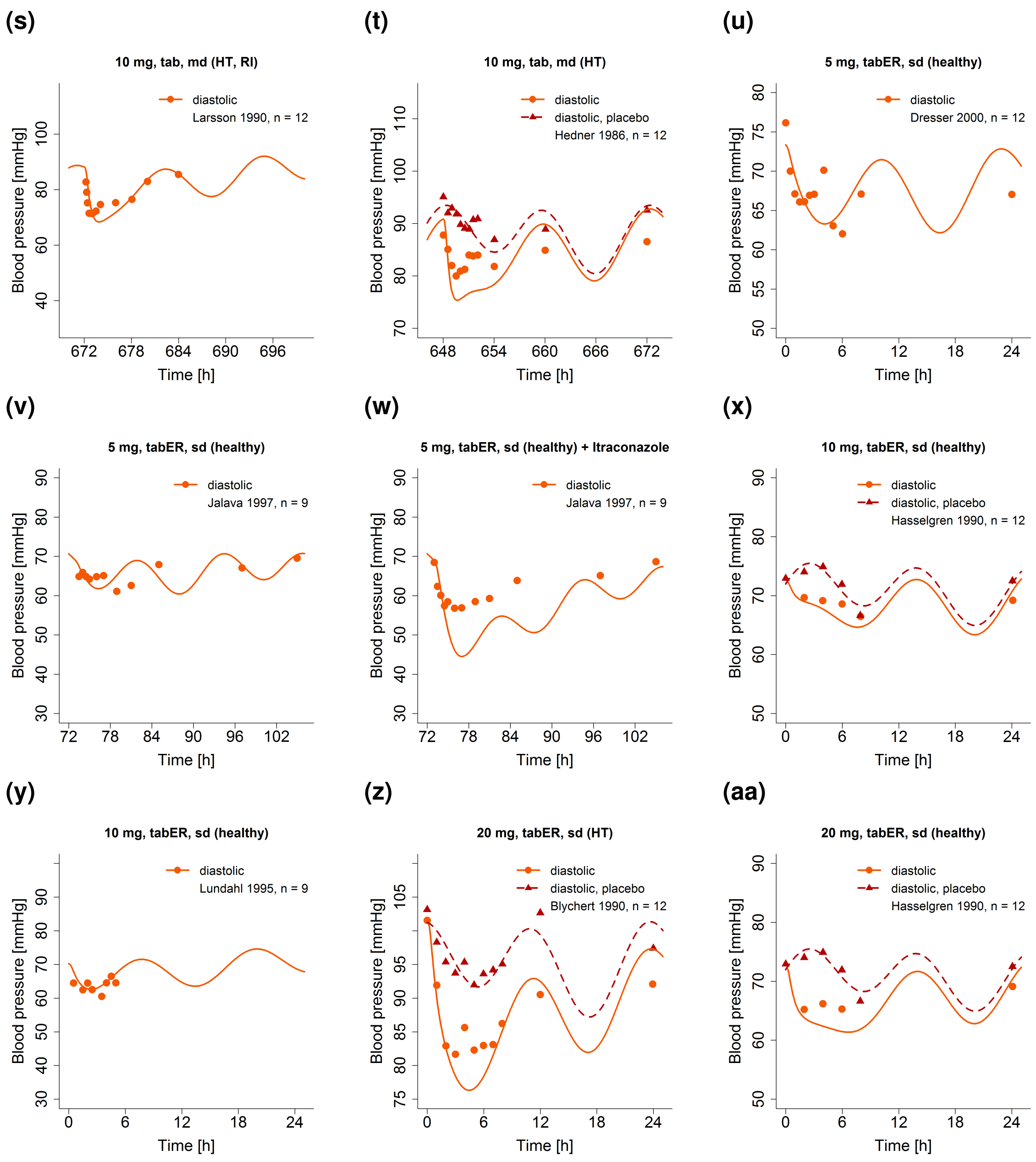

Figure S9: Predicted compared to observed effect-time profiles of diastolic blood pressure after intravenous and oral administration of felodipine. Observed data are shown as dots and triangles ± standard deviation (if available); model predictions are shown as solid lines. Details on dosing regimens, study populations and literature references are listed in Table S1. bid: twice daily, HT: hypertension, iv: intravenous, md: multiple dose, n: number of individuals, qd: once daily, RI: renal impairment, sol: solution, sd: single dose, tab: tablet, tabER: extended release tablet. *(continued)*

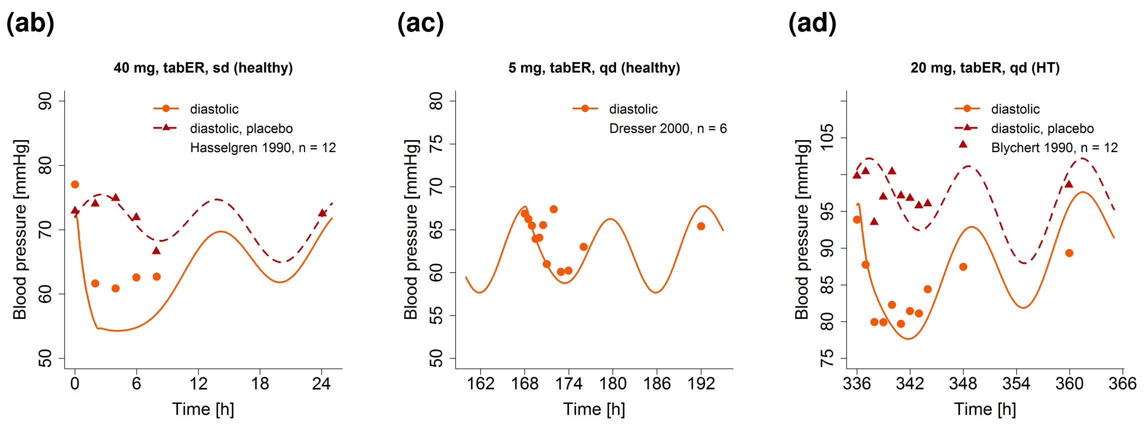

Figure S9: Predicted compared to observed effect-time profiles of diastolic blood pressure after intravenous and oral administration of felodipine. Observed data are shown as dots and triangles ± standard deviation (if available); model predictions are shown as solid lines. Details on dosing regimens, study populations and literature references are listed in Table S1. bid: twice daily, HT: hypertension, iv: intravenous, md: multiple dose, n: number of individuals, qd: once daily, RI: renal impairment, sol: solution, sd: single dose, tab: tablet, tabER: extended release tablet. *(continued)*

3.3.2 Heart rate

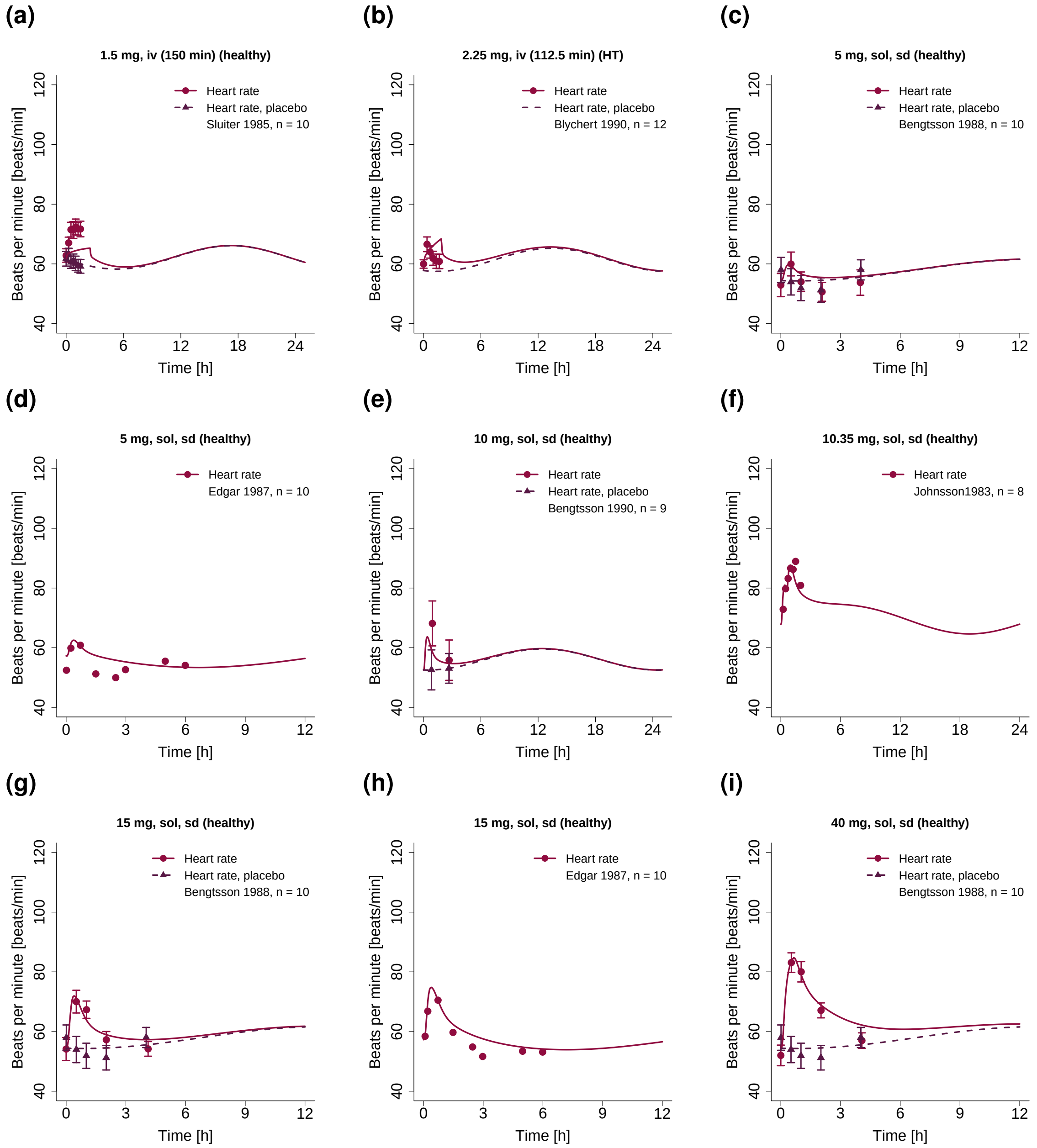

Figure S10: Predicted compared to observed effect-time profiles of heart rate after intravenous and oral administration of felodipine. Observed data are shown as dots and triangles ± standard deviation (if available); model predictions are shown as solid lines. Details on dosing regimens, study populations and literature references are listed in Table S1. bid: twice daily, HT: hypertension, iv: intravenous, md: multiple dose, n: number of individuals, qd: once daily, RI: renal impairment, sol: solution, sd: single dose, tab: tablet, tabER: extended release tablet.

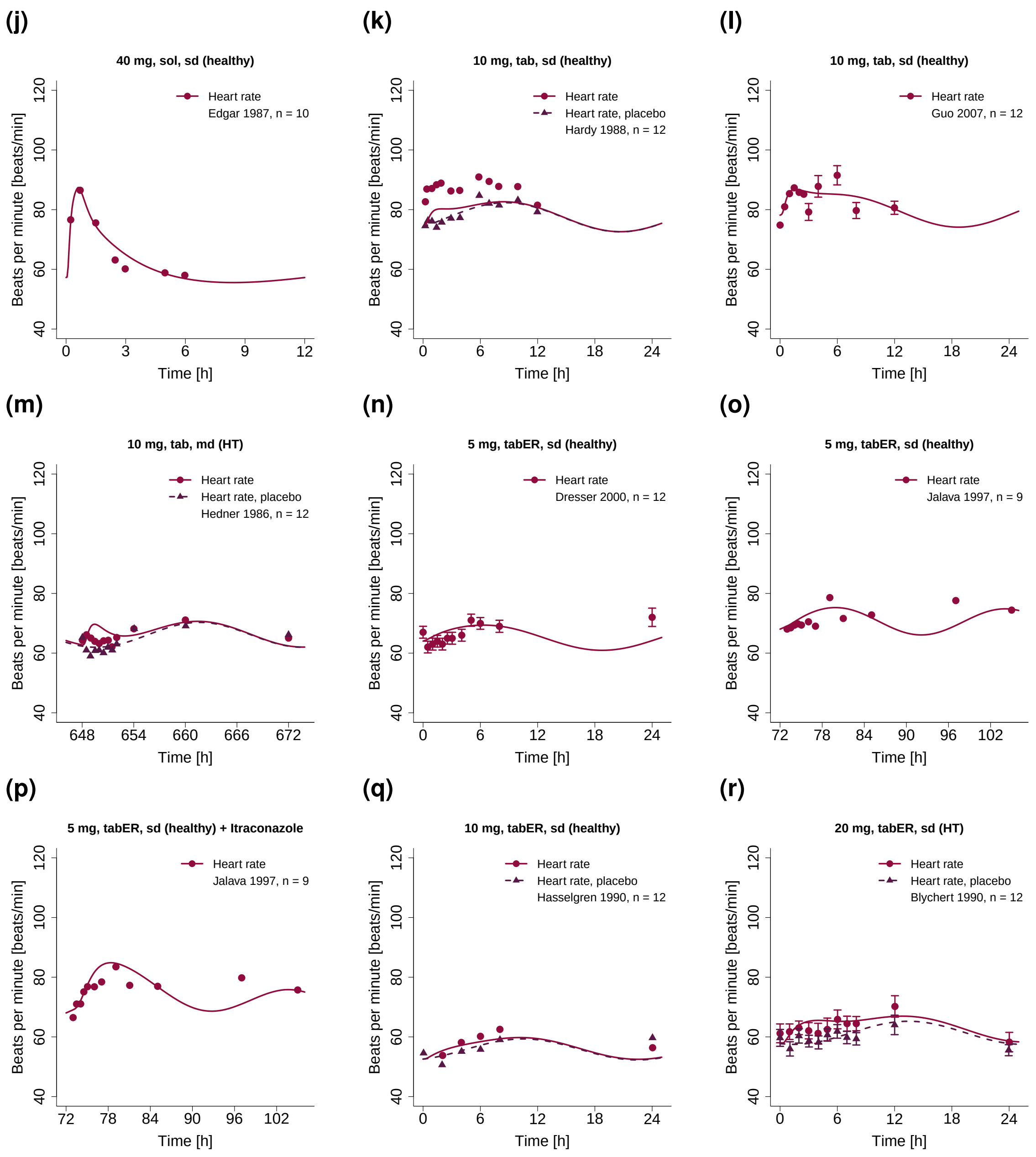

Figure S10: Predicted compared to observed effect-time profiles of heart rate after intravenous and oral administration of felodipine. Observed data are shown as dots and triangles ± standard deviation (if available); model predictions are shown as solid lines. Details on dosing regimens, study populations and literature references are listed in Table S1. bid: twice daily, HT: hypertension, iv: intravenous, md: multiple dose, n: number of individuals, qd: once daily, RI: renal impairment, sol: solution, sd: single dose, tab: tablet, tabER: extended release tablet. *(continued)*

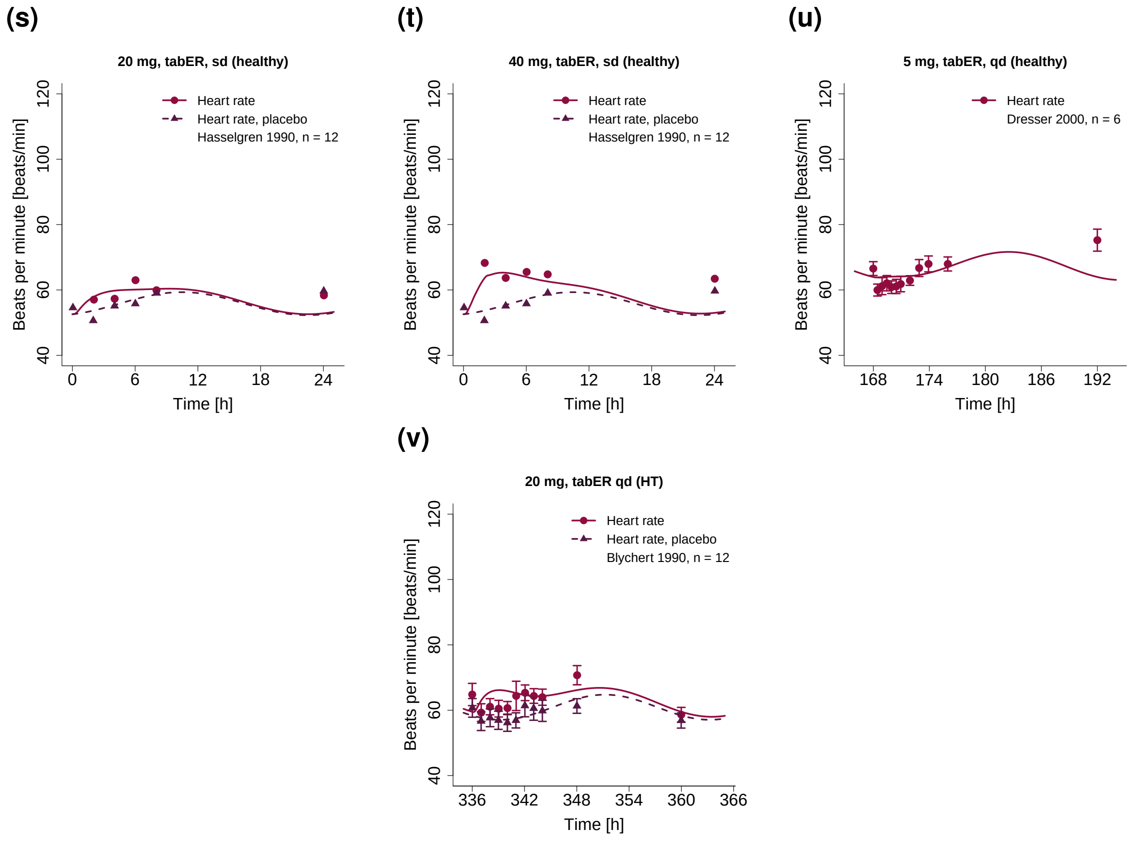

Figure S10: Predicted compared to observed effect-time profiles of heart rate after intravenous and oral administration of felodipine. Observed data are shown as dots and triangles ± standard deviation (if available); model predictions are shown as solid lines. Details on dosing regimens, study populations and literature references are listed in Table S1. bid: twice daily, HT: hypertension, iv: intravenous, md: multiple dose, n: number of individuals, qd: once daily, RI: renal impairment, sol: solution, sd: single dose, tab: tablet, tabER: extended release tablet. *(continued)*

3.4 Model evaluation

3.4.1 Goodness-of-fit plots

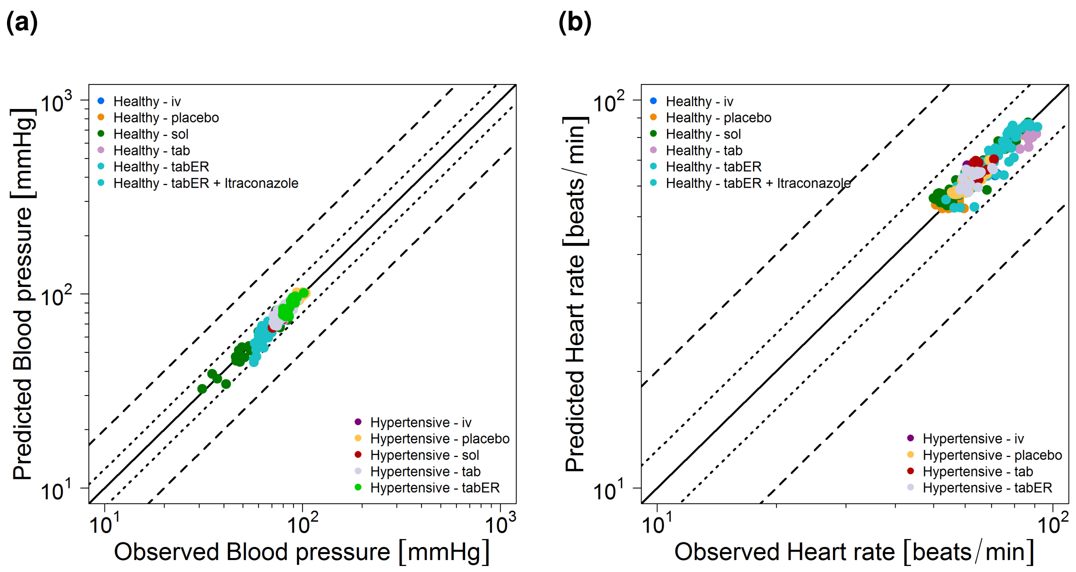

Figure S11: Predicted compared to observed (a) diastolic blood pressure and (b) heart rate measurements with and without felodipine administration. The solid line marks the line of identity. Dotted lines indicate 1.25-fold, dashed lines indicate 2-fold deviation. iv: intravenous, sol: solution, tab: tablet, tabER: extended release tablet.

3.4.2 Mean relative deviation of predicted effect measurements

Table S7: Mean relative deviation values of predicted diastolic blood pressure measurements with and without felodipine administration

Parameter	Felodipine route	Felodipine dose [mg]	MRD	Reference
Diastolic blood pressure	iv (20 min)	1.5	1.08	Sluiter 1985 [9]
Diastolic blood pressure	iv (15 min)	2.25	1.06	Blychert 1990 [50]
Diastolic blood pressure	po (sol), sd	0.83	1.02	Edgar 1985 [11]
Diastolic blood pressure	po (sol), sd	5	1.03	Bengtsson 1988 [6]
Diastolic blood pressure	po (sol), sd	5	1.03	Edgar 1987 [7]
Diastolic blood pressure	po (sol), sd	8.3	1.06	Edgar 1985 [11]
Diastolic blood pressure	po (sol), sd	10	1.06	Bengtsson 1990 [14]
Diastolic blood pressure	po (sol), sd	10.35	1.10	Johnsson 1983 [18]
Diastolic blood pressure	po (sol), sd	15	1.05	Bengtsson 1988 [6]
Diastolic blood pressure	po (sol), sd	15	1.05	Edgar 1987 [7]
Diastolic blood pressure	po (sol), sd	40	1.04	Bengtsson 1988 [6]
Diastolic blood pressure	po (sol), sd	40	1.13	Edgar 1987 [7]
Diastolic blood pressure	po (tab), sd	10	1.05	Hardy 1988 [26]
Diastolic blood pressure	po (tab), sd	10	1.03	Larsson 1990 [51]
Diastolic blood pressure	po (tab), sd	10	1.02	Larsson 1990 [51]
Diastolic blood pressure	po (tab), md	10	1.03	Hedner 1987 [52]
Diastolic blood pressure	po (tabER), qd	20	1.05	Hedner 1987 [52]
Diastolic blood pressure	po (tab), bid	10	1.05	Larsson 1990 [51]
Diastolic blood pressure	po (tab), bid	10	1.06	Larsson 1990 [51]
Diastolic blood pressure	po (tab), bid	10	1.07	Hedner 1986 [54]
Diastolic blood pressure	po (tabER), sd	5	1.05	Dresser 2000 [27]
Diastolic blood pressure	po (tabER), sd	5	1.05	Jalava 1997 [28]
Diastolic blood pressure	po (tabER), sd	5	1.14	Jalava 1997 [28]
Diastolic blood pressure	po (tabER), sd	10	1.03	Hasselgren 1990 [40]
Diastolic blood pressure	po (tabER), sd	10	1.03	Lundahl 1995 [42]
Diastolic blood pressure	po (tabER), sd	20	1.05	Blychert 1990 [50]
Diastolic blood pressure	po (tabER), sd	20	1.05	Hasselgren 1990 [40]
Diastolic blood pressure	po (tabER), sd	40	1.11	Hasselgren 1990 [40]
Diastolic blood pressure	po (tabER), qd	5	1.04	Dresser 2000 [27]
Diastolic blood pressure	po (tabER), qd	20	1.04	Blychert 1990 [50]
Diastolic blood pressure, placebo	-	-	1.02	Sluiter 1985 [9]
Diastolic blood pressure, placebo	-	-	1.00	Bengtsson 1990 [14]
Diastolic blood pressure, placebo	-	-	1.02	Bengtsson 1988 [6]
Diastolic blood pressure, placebo	-	-	1.02	Edgar 1985 [11]
Diastolic blood pressure, placebo	-	-	1.02	Hardy 1988 [26]
Diastolic blood pressure, placebo	-	-	1.02	Hedner 1986 [54]
Diastolic blood pressure, placebo	-	-	1.02	Blychert 1990 [50]
Diastolic blood pressure, placebo	-	-	1.02	Hasselgren 1990 [40]
Diastolic blood pressure, placebo	-	-	1.04	Blychert 1990 [50]
**MRD (effect)**			**1.06 (1.02-1.14)**	
**MRD (placebo)**			**1.02 (1.00-1.04)**	
**MRD (total)**			**1.05 (1.00-1.14)**	
			**39/39 with MRD < 2**	

bid: twice daily, D: day, iv: intravenous, MRD: mean relative deviation, po: oral, qd: once daily, sd: single dose, sol: solution, tab: tablet, tabER: extended release tablet

Table S8: Mean relative deviation values of predicted heart rate measurements with and without felodipine administration

Parameter	Felodipine route	Felodipine dose [mg]	MRD	Reference
Heart rate	iv (20 min)	1.5	1.10	Sluiter 1985 [9]
Heart rate	iv (15 min)	2.25	1.07	Blychert 1990 [50]
Heart rate	po (sol), sd	5	1.05	Bengtsson 1988 [6]
Heart rate	po (sol), sd	5	1.07	Edgar 1987 [7]
Heart rate	po (sol), sd	10	1.11	Bengtsson 1990 [14]
Heart rate	po (sol), sd	10.35	1.04	Johnsson1983 [18]
Heart rate	po (sol), sd	15	1.04	Bengtsson 1988 [6]
Heart rate	po (sol), sd	15	1.05	Edgar 1987 [7]
Heart rate	po (sol), sd	40	1.05	Bengtsson 1988 [6]
Heart rate	po (sol), sd	40	1.04	Edgar 1987 [7]
Heart rate	po (tab), sd	10	1.09	Hardy 1988 [26]
Heart rate	po (tab), sd	10	1.04	Guo 2007 [24]
Heart rate	po (tab), bid	10	1.05	Hedner 1986 [54]
Heart rate	po (tabER), sd	5	1.05	Dresser 2000 [27]
Heart rate	po (tabER), sd	5	1.05	Jalava 1997 [28]
Heart rate	po (tabER), sd	5	1.05	Jalava 1997 [28]
Heart rate	po (tabER), sd	10	1.04	Hasselgren 1990 [40]
Heart rate	po (tabER), sd	20	1.04	Blychert 1990 [50]
Heart rate	po (tabER), sd	20	1.05	Hasselgren 1990 [40]
Heart rate	po (tabER), sd	40	1.09	Hasselgren 1990 [40]
Heart rate	po (tabER), qd	5	1.06	Dresser 2000 [27]
Heart rate	po (tabER), qd	20	1.06	Blychert 1990 [50]
Heart rate, placebo	-	-	1.01	Sluiter 1985 [9]
Heart rate, placebo	-	-	1.00	Bengtsson 1990 [14]
Heart rate, placebo	-	-	1.05	Bengtsson 1988 [6]
Heart rate, placebo	-	-	1.02	Hardy 1988 [26]
Heart rate, placebo	-	-	1.03	Hedner 1986 [54]
Heart rate, placebo	-	-	1.03	Blychert 1990 [50]
Heart rate, placebo	-	-	1.06	Hasselgren 1990 [40]
Heart rate, placebo	-	-	1.03	Blychert 1990 [50]
**MRD (effect)**			**1.06 (1.04-1.11)**	
**MRD (placebo)**			**1.03 (1.00-1.06)**	
**MRD (total)**			**1.05 (1.00-1.11)**	
			**30/30 with MRD < 2**	

iv: intravenous, MRD: mean relative deviation, po: oral, qd: once daily, sd: single dose, sol: solution, tab: tablet, tabER: extended release tablet

4 Felodipine DDIs

4.1 DDI modeling

Three DDI studies, providing (1) three victim drug plasma concentration-time profiles of felodipine (2) one plasma concentration-time profile of dehydrofelodipine and (3) one blood pressure and heart rate effect-time profile, were utilized to evaluate the DDI performance of the felodipine PBPK model. In those studies, (1) the mechanism-based CYP3A4 inhibitor erythromycin, (2) the competitive CYP3A4 inhibitor itraconazole and (3) the CYP3A4 inducers carbamazepine and phenytoin were used as perpetrators of felodipine metabolism. The implementation of these DDIs is described in more detail in the following sections.

4.2 Erythromycin-felodipine DDI

The erythromycin-felodipine DDI was modeled using a previously developed and evaluated whole-body PBPK model of erythromycin, available in the Open Systems Pharmacology (OSP) GitHub model repository (https://github.com/Open-Systems-Pharmacology/Erythromycin-Model). [69, 70].

The implementation of the erythromycin-felodipine DDI is illustrated in Figure S12. DDI parameters were used as implemented in the erythromycin model. Drug-dependent parameters of the erythromycin PBPK model are displayed in Table S9.

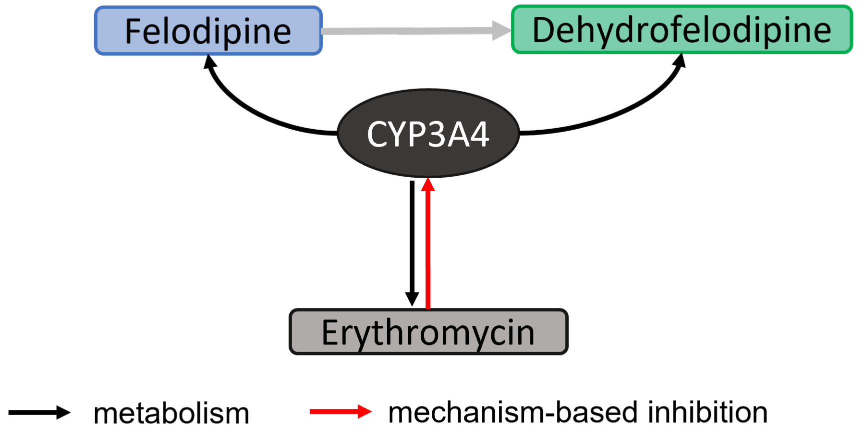

Figure S12: Implementation of the erythromycin-felodipine DDI. Erythromycin inhibits the CYP3A4 mediated metabolism of felodipine and dehydrofelodipine in a mechanism-based manner. As erythromycin is also substrate of CYP3A4, the compound also inhibits its own metabolism. CYP: cytochrome P450

Details on the modeled clinical DDI studies are given in Table S10. Predicted felodipine and dehydrofelodipine plasma concentration-time profiles with and without erythromycin co-administration, compared to observed data, are shown in Figures S13 (linear) and S14 (semi-logarithmic). The correlation of predicted to observed DDI AUC_{last} and C_{max} ratios is shown in Figure S15. Table S11 lists the corresponding predicted and observed DDI AUC_{last} ratios, DDI C_{max} ratios, as well as GMFE values.

4.2.1 Erythromycin drug-dependent parameters

Table S9: Drug-dependent parameters of the erythromycin PBPK model according to [69]

Parameter	Unit	Model	Literature	Reference	Description
MW	g/mol	733.9 (lit)	733.9	[71]	Molecular weight
logP	Log Units	2.82 (lit)	2.82 (2.48-3.06)[a]	[72–74]	Lipophilicity
Solubility (pH)	mg/ml	200.0 (7.0) (lactobionate) (lit),	200.0 (7.0) (lactobionate),	[75]	Solubility
		0.028 (7.0) (stearate) (opt),	0.182 (7.0) (stearate),	[76]	
		0.50 (7.0) (base pellets) (opt),	2.10 (7.0) (base)	[77]	
		0.0084 (7.0) (base tablet) (opt)			
fu	%	30.5 (lit)	27.0, 28.0, 30.5, 32.6	[78–81]	Fraction unbound in plasma
pKa (base)	-	8.88 (lit)	8.88	[74]	Acid dissociation constant
K_m (CYP3A4)	µmol/l	70.0 (lit)	70 (44.0-88.0)[a]	[82, 83]	CYP3A4 Michaelis-Menten constant
k_{cat} (CYP3A4)	1/min	8.50 (opt)	-	-	CYP3A4 catalytic rate constant
K_m (OATP1B1)	µmol/l	0.74 (opt)	13.2	[84]	OATP1B1 Michaelis-Menten constant
k_{cat} (OATP1B1)	1/min	2.02 (opt)	-	-	OATP1B1 transport rate constant
CL_{hep}	1/min	4.15 (opt)	-	-	Hepatic plasma clearance
GFR fraction	-	1.16 (opt)	-	-	Fraction of filtered drug in the urine
K_i (CYP3A4)	µmol/l	7.60 (opt)	18.4 (0.76-109.0)[b]	[81, 85–95]	Concentration for half-maximal inactivation
k_{inact} (CYP3A4)	1/min	0.03 (opt)	0.06 (0.01-0.30)[b]	[81, 85–95]	Maximum inactivation rate constant
Intestinal permeability	cm/min	3.87E-04 (opt)	-	-	Transcellular intestinal permeability
Partition coefficients	-	Diverse	Rogers and Rowland	[64, 65]	Cell to plasma partition coefficients
Cellular permeability	cm/min	1.22E-4 (calc)	Charge-dependent Schmitt	[1]	Permeability into the cellular space
$t_{50\%}$ (film tablet)	min	79.63 (opt)	-	-	Dissolution time (50% dissolved)
s (film tablet)	-	1.08 (opt)	-	-	Dissolution profile shape

-: not given, calc: calculated, CYP3A4: cytochrome P450 3A4, GFR: glomerular filtration rate, lit: literature, OATP1B1: organic anion transporting polypeptide 1B1, opt: optimized during parameter identification

[a] mean (range)

4.2.2 Erythromycin-felodipine clinical studies

Table S10: Clinical studies investigating the erythromycin-felodipine DDI

Erythromycin administration		Felodipine administration		Interval [h]	n	Healthy [%]	Females [%]	Age[a] [years]	Weight [kg]	Reference
Dose [mg]	Route	Dose [mg]	Route							
250	po , qid D1-D2	10	po (tabER), sd D2	0	12	100	0	(18-45)	-	Bailey et al. 1996 [35]

-: not given, D: day, po: oral, n: number of individuals, qid: four times daily, sd: single dose, tabER: extended release tablet

[a] range

4.2.3 Plasma concentration-time profiles

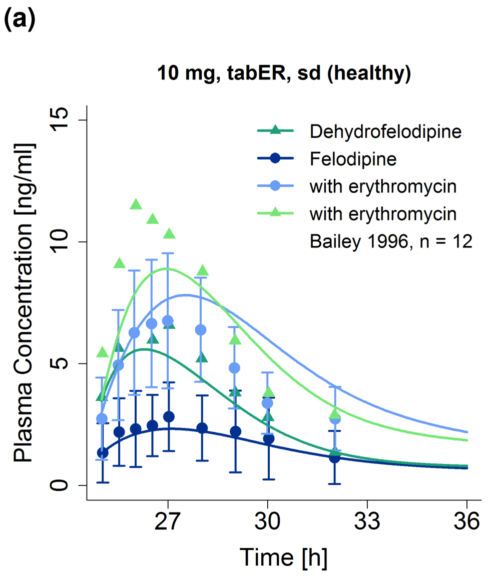

Figure S13: Predicted compared to observed felodipine and dehydrofelodipine plasma concentration-time profiles (linear) before and during erythromycin co-administration. Observed data are shown as dots and triangles ± standard deviation; model predictions are shown as solid lines. Details on dosing regimens, study population and literature reference are listed in Table S10. n: number of individuals, sd: single dose, tabER: extended release tablet.

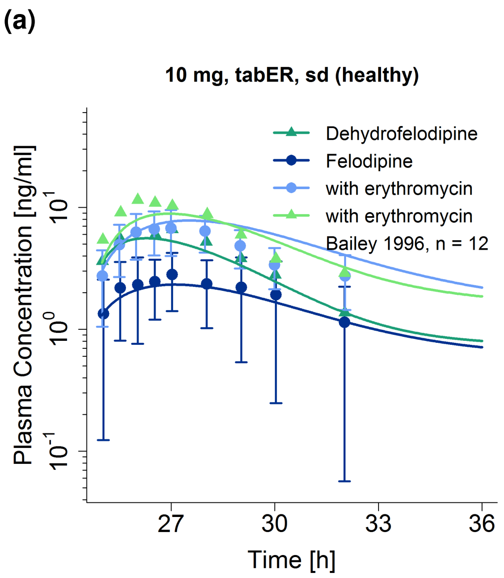

Figure S14: Predicted compared to observed felodipine and dehydrofelodipine plasma concentration-time profiles (semi-logarithmic) before and during erythromycin co-administration. Observed data are shown as dots and triangles ± standard deviation; model predictions are shown as solid lines. Details on dosing regimens, study population and literature reference are listed in Table S10. n: number of individuals, sd: single dose, tabER: extended release tablet.

4.2.4 DDI AUC_{last} and C_{max} ratio goodness-of-fit plots

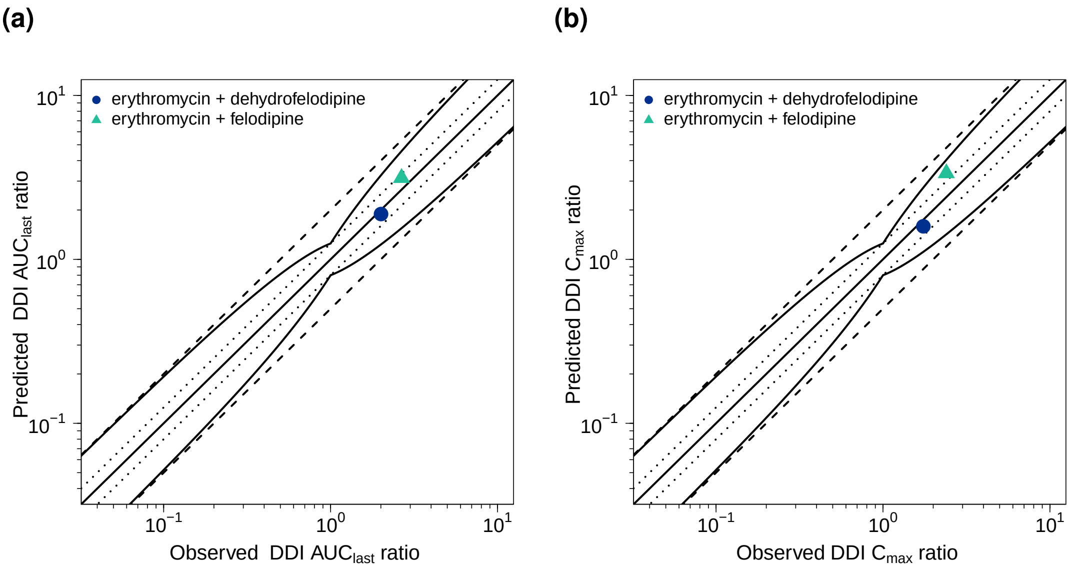

Figure S15: Predicted compared to observed erythromycin-felodipine DDI AUC_{last} and DDI C_{max} values. The straight solid line marks the line of identity, the curved solid lines show the prediction success limits proposed by Guest et al. allowing for 1.25-fold variability of the DDI ratio [96]. Dotted lines indicate 1.25-fold, dashed lines indicate 2-fold deviation. AUC_{last}: area under the plasma concentration-time curve from the time of drug administration to the last concentration measurement, C_{max}: maximum plasma concentration, DDI: drug-drug interaction

4.2.5 Geometric mean fold error of predicted DDI AUC_{last} and C_{max} ratios

Table S11: Predicted and observed erythromycin-felodipine DDI AUC_{last} ratios and DDI C_{max} ratios

**Perpetrator**	**Victim**	**Compound**	**Dose gap [h]**	**n**	**DDI AUC_{last} ratio**			**DDI C_{max} ratio**			
Erythromycin	Felodipine				**Pred**	**Obs**	**Pred/Obs**	**Pred**	**Obs**	**Pred/Obs**	**Reference**
250 mg, po, qid D1-D2	10 mg, po (tabER), sd D2	Felodipine	0	12	3.14	2.66	1.18	3.35	2.40	1.40	Bailey 1996 [35]
250 mg, po, qid D1-D2	10 mg, po (tabER), sd D2	Dehydrofelodipine	0	12	1.89	2.00	0.94	1.59	1.74	0.91	Bailey 1996 [35]
**GMFE**							**1.12 (1.06-1.18)**			**1.25 (1.10-1.40)**	
							**2/2 with GMFE < 2**			**2/2 with GMFE < 2**	

AUC_{last}: area under the plasma concentration-time curve calculated from the time of drug administration to the time of the last concentration measurement, C_{max}: maximum plasma concentration, D: day, DDI: drug-drug interaction, GMFE: geometric mean fold error, n: number of individuals, obs: observed, pred: predicted, po: oral, qid: four times daily, sd: single dose, tabER: extended release tablet

4.3 Itraconazole-felodipine DDI

The parent-metabolite PBPK model of itraconazole and its three metabolites was previously developed and evaluated for the prediction of CYP3A4 mediated DDIs [97]. The model can be freely downloaded from the OSP repository on GitHub (https://github.com/Open-Systems-Pharmacology/Itraconazole-Model). The implementation of the itraconazole-felodipine DDI is illustrated in Figure S16. Inhibition parameters were used as implemented in the itraconazole parent-metabolite model. Drug-dependent parameters of all compounds of the parent-metabolite PBPK model are displayed in Table S12. No information on the itraconazole formulation was provided in the itraconazole-felodipine DDI study. However, the itraconazole plasma concentration-time profile was best described assuming administration as oral solution.

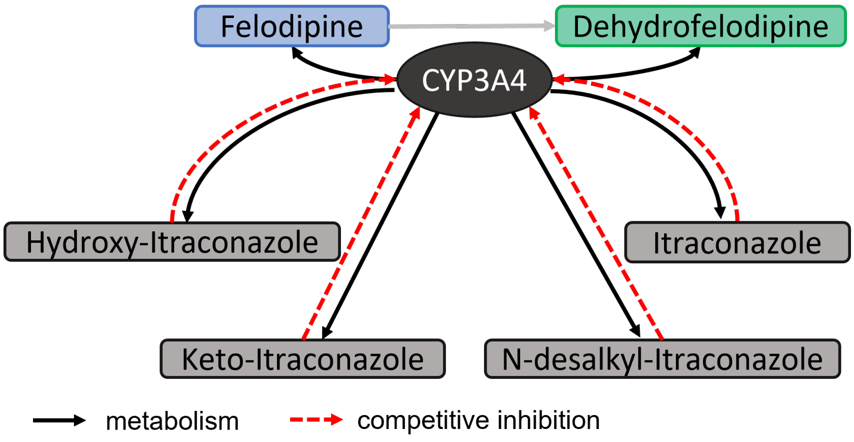

Figure S16: Implementation of the itraconazole-felodipine DDI. The CYP3A4 metabolism of felodipine and dehydrofelodipine is inhibited competitively by itraconazole and its three metabolites. Itraconazole and its metabolites are substrates of CYP3A4 as well and, therefore, inhibit each other's metabolism. CYP 3A4: cytochrome P450 3A4

Details on the modeled clinical DDI study are given in Table S13. Predicted felodipine plasma concentration-time profiles before and during itraconazole co-administration, compared to observed data, are shown in Figures S17 (linear) and S18 (semi-logarithmic). Predicted compared to observed blood pressure and heart rate effect-time profiles before and during itraconazole co-administration are shown in Figure S19. The correlation of predicted to observed DDI AUC_{last} and C_{max} ratios is shown in Figure S20. Table S14 lists the corresponding predicted and observed DDI AUC_{last} ratios, DDI C_{max} ratios, as well as GMFE values.

4.3.1 Itraconazole drug-dependent parameters

Table S12: Drug-dependent parameters of the itraconazole parent-metabolite PBPK model according to [97]

Parameter	Unit	Model	Literature	Reference	Description
**Itraconazole**					
MW	g/mol	705.633 (lit)	705.633		Molecular weight
pKa (basic)		3.7 (lit)	3.7	[98]	Acid dissociation constant
Solubility - solution fed	mg/l	1.58 (opt)	-	-	Solubility
logP		4.62 (opt)	5.66	[98]	Lipophilicity
fu	%	0.6 (lit)	0.2, 0.6, 1.6, 3.6	[98–101]	Fraction unbound in plasma
CYP3A4 K_m	nmol/l	2.07 (opt)	3.9	[102]	CYP3A4 Michaelis-Menten constant
CYP3A4 k_{cat}	1/min	0.04 (opt)	-	-	CYP3A4 catalytic rate constant
GFR fraction		1	-	-	Fraction of filtered drug in the urine
K_i CYP3A4	nmol/l	1.3 (lit)	1.3	[102]	Concentration for half-maximal inhibition
Cell permeabilities		calculated	PK-Sim Standard	[66]	Permeability into the cellular space
Partition coefficients		calculated	Rodgers and Rowland	[64, 65]	Cell to plasma partition coefficients
Intestinal permeability	cm/min	0.00000533	-	-	Transcellular intestinal permeability
Intestinal permeability	cm/min	0.000144 (calc)	-	-	Normalized to surface area
**Hydroxyitraconazole**					
MW	g/mol	721.633 (lit)	721.633		Molecular weight
pKa		3.7 (basic) (asm)	-	-	Acid dissociation constant
Solubility [pH]	mg/l	-	-	-	Solubility
logP		3.72 (opt)	4.5	[103]	Lipophilicity
fu	%	1.7 (lit)	1.7	[100]	Fraction unbound in plasma
CYP3A4 K_m	nmol/l	4.17 (opt)	27	[102]	CYP3A4 Michaelis-Menten constant
CYP3A4 k_{cat}	1/min	0.02 (opt)	-	-	CYP3A4 catalytic rate constant
GFR fraction		1 (asm)	-	-	Fraction of filtered drug in the urine
K_i CYP3A4	nmol/l	14.4 (lit)	14.4	[102]	Concentration for half-maximal inhibition
Cell permeabilities		calculated	PK-Sim Standard	[66]	Permeability into the cellular space
Partition coefficients		calculated	Rodgers and Rowland	[64, 65]	Cell to plasma partition coefficients
Intestinal permeability	cm/min	0.000000152 (calc)	-	-	Transcellular intestinal permeability
Cellular permeability	cm/min	0.0000155 (calc)	-	-	Normalized to surface area
**Keto-itraconazole**					
MW	g/mol	719.617 (lit)	-	-	Molecular weight

asm: assumed, CYP3A4: cytochrome P450 3A4, GFR: glomerular filtration rate, lit: literature, opt: optimized during parameter identification

Table S12: Drug-dependent parameters of the itraconazole parent-metabolite PBPK model according to [97] *(continued)*

Parameter	Unit	Model	Literature	Reference	Description
pKa		3.7 (basic) (asm)	-	-	Acid dissociation constant
logP		4.21 (opt)	4.5	[104]	Lipophilicity
fu	%	1 (lit)	1	[100]	Fraction unbound in plasma
CYP3A4 K_m	nmol/l	2.22 (opt)	1.4	[102]	CYP3A4 Michaelis-Menten constant
CYP3A4 k_{cat}	1/min	0.393 (opt)	-	-	CYP3A4 catalytic rate constant
GFR fraction		1 (asm)	-	-	Fraction of filtered drug in the urine
K_i CYP3A4	nmol/l	5.12[a] (lit)	5.12 [a]	[102]	Concentration for half-maximal inhibition
Cell permeabilities		calculated	PK-Sim Standard	[66]	Permeability into the cellular space
Partition coefficients		calculated	Rodgers and Rowland	[64, 65]	Cell to plasma partition coefficients
Intestinal permeability	cm/min	0.000000479 (calc)	-	-	Transcellular intestinal permeability
Cellular permeability	cm/min	0.0000492 (calc)	-	-	Normalized to surface area
**N-desalkyl-itraconazole**					
MW	g/mol	649.527 (lit)	-	-	Molecular weight
pKa		3.7 (base) (asm)	-	-	Acid dissociation constant
logP		5.18 (opt)	4.2	[105]	Lipophilicity
fu	%	1.1 (lit)	1.1	[100]	Fraction unbound in plasma
CYP3A4 K_m	nmol/l	0.63 (opt)	-	-	CYP3A4 Michaelis-Menten constant
CYP3A4 k_{cat}	1/min	0.061 (opt)	-	-	CYP3A4 catalytic rate constant
GFR fraction		1	-	-	Fraction of filtered drug in the urine
K_i CYP3A4	nmol/l	0.32[a] (lit)	0.32	[102]	Concentration for half-maximal inhibition
Cell permeabilities		calculated	PK-Sim Standard	[66]	Permeability into the cellular space
Partition coefficients		calculated	Rodgers and Rowland	[64, 65]	Cell to plasma partition coefficients
Cellular permeability	cm/min	0.00000737 (calc)	-	-	Transcellular intestinal permeability
Intestinal permeability	cm/min	0.000891 (calc)	-	-	Normalized to surface area

asm: assumed, CYP3A4: cytochrome P450 3A4, GFR: glomerular filtration rate, lit: literature, opt: optimized during parameter identification

4.3.2 Itraconazole-felodipine clinical studies

Table S13: Clinical studies investigating the itraconazole-felodipine DDI

Itraconazole administration		Felodipine administration		Interval [h]	n	Healthy [%]	Females [%]	Age[a] [years]	Weight[a] [kg]	Reference
Dose [mg]	Route	Dose [mg]	Route							
200	po (*sol*), qd D1-D4	5	po (tabER), sd (D4)	1	9	100	44.4	24.44 (22-26)	59.88 (51-73)	Jalava 1997 [28]

assumed

D: day, n: number of individuals, po: oral, qd: once daily, sd: single dose, sol: solution, tabER: extended release tablet

[a] mean (range)

4.3.3 Plasma concentration-time and effect-time profiles

(a)

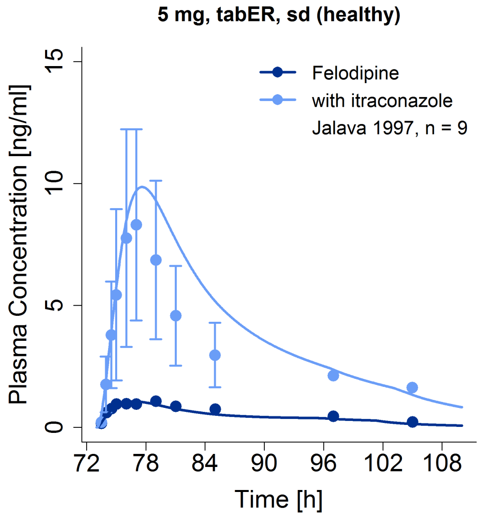

Figure S17: Predicted compared to observed felodipine plasma concentration-time profiles (linear) before and during itraconazole co-administration. Observed data are shown as dots ± standard deviation; model predictions are shown as solid lines. Details on dosing regimens, study population and literature reference are listed in Table S13. n: number of individuals, sd: single dose, tabER: extended release tablet

(a)

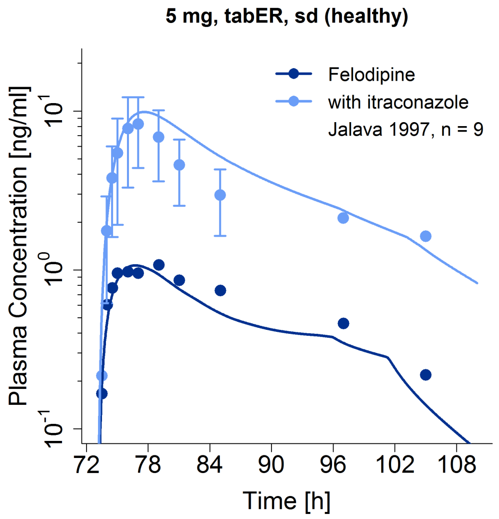

Figure S18: Predicted compared to observed felodipine plasma concentration-time profiles (semi-logarithmic) before and during itraconazole co-administration. Observed data are shown as dots ± standard deviation; model predictions are shown as solid lines. Details on dosing regimens, study population and literature reference are listed in Table S13. n: number of individuals, sd: single dose, tabER: extended release tablet.

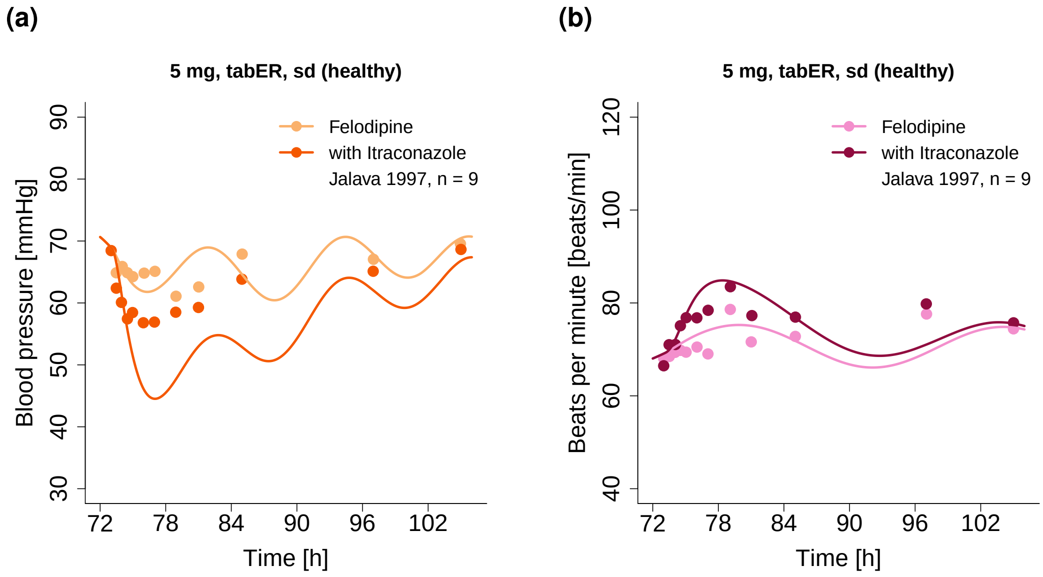

Figure S19: Predicted compared to observed (a) blood pressure and (b) heart rate effect-time profiles before and during itraconazole co-administration. Observed data are shown as dots; model predictions are shown as solid lines. Details on dosing regimens, study population and literature reference are listed in Table S13. n: number of individuals, sd: single dose, tabER: extended release tablet.

4.3.4 DDI AUC_{last} and C_{max} ratio goodness-of-fit plots

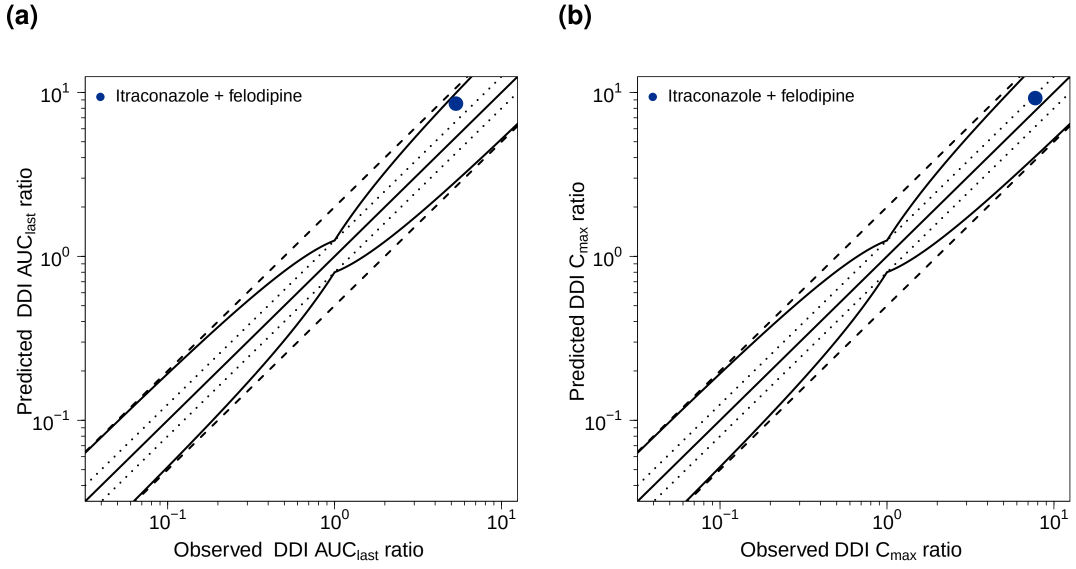

Figure S20: Predicted compared to observed itraconazole-felodipine DDI AUC_{last} and DDI C_{max} values. The straight solid line marks the line of identity, the curved solid lines show the prediction success limits proposed by Guest et al. allowing for 1.25-fold variability of the DDI ratio [96]. Dotted lines indicate 1.25-fold, dashed lines indicate 2-fold deviation. AUC_{last}: area under the plasma concentration-time curve from the time of drug administration to the last concentration measurement, C_{max}: maximum plasma concentration, DDI: drug-drug interaction

4.3.5 Geometric mean fold error of predicted DDI AUC_{last} and C_{max} ratios

Table S14: Predicted and observed itraconazole-felodipine DDI AUC_{last} ratios and DDI C_{max} ratios

**Perpetrator**	**Victim**	**Compound**	**Dose gap [h]**	**n**	**DDI AUC_{last} ratio**			**DDI C_{max} ratio**			
Itraconazole	Felodipine				**Pred**	**Obs**	**Pred/Obs**	**Pred**	**Obs**	**Pred/Obs**	**Reference**
200 mg po (*sol*), qd D1-D4	5, mg, po (tabER), sd D4	Felodipine	1	9	8.54	5.33	1.60	9.23	7.73	1.19	Jalava 1997 [28]
**GMFE**							**1.60**			**1.19**	
							**1/1 with GMFE < 2**			**1/1 with GMFE < 2**	

AUC_{last}: area under the plasma concentration-time curve calculated from the time of drug administration to the time of the last concentration measurement, C_{max}: maximum plasma concentration, D: day, DDI: drug-drug interaction, GMFE: geometric mean fold error, n: number of individuals, obs: observed, pred: predicted, po: oral, qd: once daily, sd: single dose, sol: solution, tabER: extended release tablet

4.4 Carbamazepine-felodipine DDI

Previously developed PBPK models of carbamazepine [106] and phenytoin (unpublished, in house) were available and already evaluated for the prediction of CYP3A4 mediated DDIs. The implementation of the carbamazepine-phenytoin-felodipine DDI is illustrated in Figure S21. Interaction parameters were used as implemented previously in the perpetrator models. Drug-dependent parameters of carbamazepine are shown in Table S15.

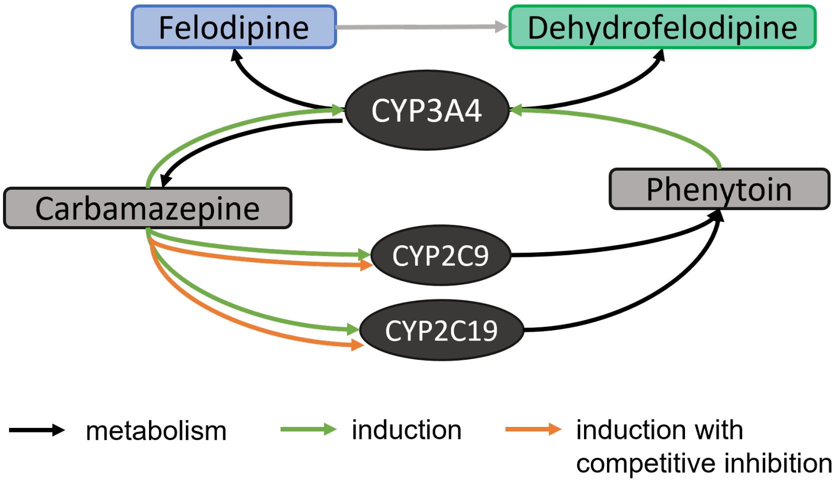

Figure S21: Implementation of the carbamazepine-phenytoin-felodipine DDI. The CYP3A4 metabolism of felodipine and dehydrofelodipine is induced by carbamazepine and phenytoin. The CYP3A4 metabolism of carbamazepine is induced by carbamazepine itself as well as phenytoin. The CYP2C9 and CYP2C19 metabolism of phenytoin is competitively inhibited and induced by carbamazepine. CYP: cytochrome P450

Details on the modeled clinical DDI study are given in Table S16. Predicted felodipine plasma concentration-time profiles with and without carbamazepine/phenytoin co-administration, compared to observed data, are shown in Figures S22 (linear) and S23 (semi-logarithmic). The correlation of predicted to observed DDI AUC_{last} and C_{max} ratios is shown in Figure S24. Table S17 lists the corresponding predicted and observed DDI AUC_{last} ratios, DDI C_{max} ratios, as well as GMFE values.

4.4.1 Carbamazepine drug-dependent parameters

Table S15: Drug-dependent parameters of the carbamazepine and carbamazepine-10,11-epoxide PBPK model according to [106]

Parameter	Unit	Model	Literature	Reference	Description
**Carbamazepine**					
MW	g/mol	236.27 (Lit)	236.27	[109]	Molecular weight
logP	Log Units	2.00 (Fit)	1.45, 2.10, 2.45, 2.77	[109–111]	Lipophilicity
Solubility (pH)	mg/ml	0.336 (6.2) (Lit)	0.170 (6.2), 0.283 (7.0), 0.306 (6.9), 0.336 (6.2)	[112–115]	Solubility FaHIF
fu	%	25.0 (Lit)	21.0, 24.0, 25.0	[116–119]	Fraction unbound in plasma
K_m (CYP3A4) CBZE	µmol/l	248.0 (Lit)	119.0, 248.0, 442.0, 630.0	[120–123]	CYP3A4 Michaelis-Menten constant
k_{cat} (CYP3A4) CBZE	1/min	0.75 (Fit)	1.17, 1.70, 4.87, 5.30[b]	[120–123]	CYP3A4 catalytic rate constant
K_m (CYP2C8)	µmol/l	757.0 (Lit)	757.0	[122]	CYP2C8 Michaelis-Menten constant
k_{cat} (CYP2C8)	1/min	0.67 (Lit)	0.67[b]	[122]	CYP2C8 catalytic rate constant
K_m (CYP2B6)	µmol/l	420.0 (Lit)	420.0	[124]	CYP2B6 Michaelis-Menten constant
k_{cat} (CYP2B6)	1/min	0.43 (Lit)	0.43[b]	[124]	CYP2B6 catalytic rate constant
K_m (CYP3A4)	µmol/l	282.0 (Lit)	282.0	[124]	CYP3A4 Michaelis-Menten constant
k_{cat} (CYP3A4)	1/min	0.20 (Fit)	0.16[b]	[124]	CYP3A4 catalytic rate constant
K_m (UGT2B7)	µmol/l	214.0 (Lit)	214.0	[107]	UGT2B7 Michaelis-Menten constant
k_{cat} (UGT2B7)	1/min	9.53E-3 (Lit)	9.53E-3[c]	[107]	UGT2B7 catalytic rate constant
CL_{hep}	1/min	0.02 (Fit)	-	-	Unspecified hepatic clearance
GFR fraction	-	0.03 (Fit)	-	-	Fraction of filtered drug in the urine
EC_{50} (CYP3A4)	µmol/l	20.00[a] (Lit)	4.3 - 137	[125–132]	Concentration for half-maximal induction
E_{max} (CYP3A4)	-	6.00 (Fit)	1.90 - 23.0	[125–132]	CYP3A4 maximum induction effect
EC_{50} (CYP2B6)	µmol/l	20.0[a] (Asm)	22 - 145	[132–134]	Concentration for half-maximal induction
E_{max} (CYP2B6)	-	17.0 (Fit)	3.10 - 21.50	[132–134]	CYP2B6 maximum induction effect
EC_{50} (EPHX1)	µmol/l	20.0[a] (Asm)	-	-	Concentration for half-maximal induction
E_{max} (EPHX1)	-	3.25 (Fit)	-	-	EPHX1 maximum induction effect
EC_{50} (CYP2C9)	µmol/l	20.0[a] (Asm)	-	-	Concentration for half-maximal induction
E_{max} (CYP2C9)	-	1.83 (Lit)	1.83	[132]	CYP2C9 maximum induction effect

asm: assumption, calc: calculated, CBZE: carbamazepine-10,11-epoxide, CL_{hep}: hepatic clearance, CL_{spec}: specific clearance, CYP: cytochrome P450, EPHX1: epoxide hydroxylase 1, FaHIF: fasted human intestinal fluid, IR: immediate release, fit: optimized during parameter optimization, lit: literature, UGT: UDP-glucuronosyltransferase, XR: extended release

[a] mean of literature values for EC_{50} (CYP3A4), assumed for all EC_{50} values

[b] k_{cat} values calculated within PK-Sim from V_{max}/recombinant enzyme

[c] k_{cat} value calculated within PK-Sim from V_{max} = 0.79 pmol/min/microsomal protein [107], assuming a microsomal UGT2B7 content of 82.9 pmol/mg microsomal protein [108], kcat = Vmax/ UGT2B7 content microsomes

Table S15: Drug-dependent parameters of the carbamazepine and carbamazepine-10,11-epoxide PBPK model according to [106] *(continued)*

**Parameter**	**Unit**	**Model**	**Literature**	**Reference**	**Description**
EC_{50} (CYP2C19)	µmol/l	20.0[a] (Asm)	-	-	Concentration for half-maximal induction
E_{max} (CYP2C19)	-	1.24 (Lit)	1.24	[132]	CYP2C19 maximum induction effect
K_i (CYP2C9)	µmol/l	90 (Lit)	90	[135]	Concentration for half-maximal inhibition
K_i (CYP2C19)	µmol/l	8 (Lit)	8	[135]	Concentration for half-maximal inhibition
Intestinal permeability	cm/min	2.58E-2 (Lit)	2.58E-2	[136]	Transcellular intestinal permeability
Partition coefficients	-	Diverse	Rodgers and Rowlands	[64, 65]	Cell to plasma partition coefficients
Cellular permeability	cm/min	0.02 (Calc)	PK-Sim Standard	[66]	Permeability into the cellular space
$t_{50\%}$ (IR tablet, fasted))	min	200.0	-	-	Dissolution time (50% dissolved)
s (IR tablet, fasted))	-	0.74	-	-	Dissolution profile shape
**Carbamazepine-10,11-epoxide**					
logP	Log Units	1.16 (Fit)	1.58, 1.97	[137]	Lipophilicity
Solubility	mg/ml	1.34 (Lit)	1.34	[137]	Solubility
fu	%	51.8 (Lit)	46.8-51.8	[138]	Fraction unbound in plasma
CL_{spec} (EPHX1)	1/min	0.01 (Fit)	0.05	-	EPHX1 first-order clearance
GFR fraction	-	0.21 (Fit)	-	-	Fraction of filtered drug in the urine
Intestinal permeability	cm/min	0.3 (Fit)	-	-	Transcellular intestinal permeability
Partition coefficients	-	Diverse	Rodgers and Rowlands	[64, 65]	Cell to plasma partition coefficients
Cellular permeability	cm/min	1.61E-3 (Calc)	PK-Sim Standard	[66]	Permeability into the cellular space

asm: assumption, calc: calculated, CBZE: carbamazepine-10,11-epoxide, CL_{hep}: hepatic clearance, CL_{spec}: specific clearance, CYP: cytochrome P450, EPHX1: epoxide hydroxylase 1, FaHIF: fasted human intestinal fluid, IR: immediate release, fit: optimized during parameter optimization, lit: literature, UGT: UDP-glucuronosyltransferase, XR: extended release

[a] mean of literature values for EC_{50} (CYP3A4), assumed for all EC_{50} values

[b] k_{cat} values calculated within PK-Sim from V_{max}/recombinant enzyme

[c] k_{cat} value calculated within PK-Sim from V_{max} = 0.79 pmol/min/microsomal protein [107], assuming a microsomal UGT2B7 content of 82.9 pmol/mg microsomal protein [108], kcat = Vmax/ UGT2B7 content microsomes

4.4.2 Carbamazepine-phenytoin-felodipine clinical studies

Table S16: Clinical studies investigating the carbamazepine-phenytoin DDI

Carbamazepine/phenytoin administration		Felodipine administration		Interval [h]	n	Healthy [%]	Females [%]	Age[a] [years]	Weight[a] [kg]	Reference
**Dose [mg]**	**Route**	**Dose [mg]**	**Route**							
757/325	po, md	5	po (tab), bid	-	10	0	10	28.6 ± 8.6	74 ± 15.6	Capewell 1988 [23]*
-	-	5	po (tab), bid D1-D5	-	12	100	8	28.7 ± 8.9	71.1 ± 9.0	Capewell 1988 [23]*

-: not given, bid: twice daily, D: day, md: multiple dose, n: number of individuals, po: oral, tab: tablet

[a] mean ± standard deviation

* control study (lower row) in healthy individuals, DDI study (upper row) in epileptic patients

4.4.3 Plasma concentration-time profiles

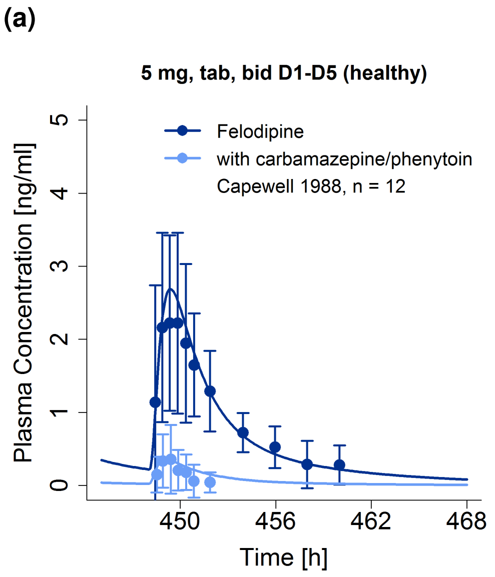

Figure S22: Predicted compared to observed felodipine plasma concentration-time profiles (linear) without and during carbamazepine and phenytoin co-administration. Observed data are shown as dots; model predictions are shown as solid lines ± standard deviation. Details on dosing regimens, study population and literature reference are listed in Table S16. bid: twice daily, D: day, n: number of individuals, tab: tablet.

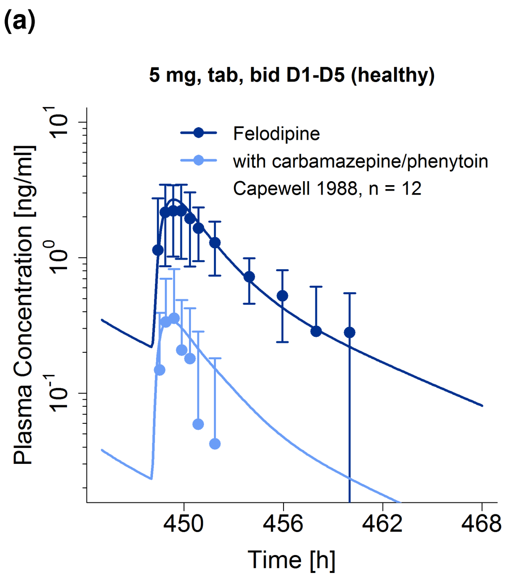

Figure S23: Predicted compared to observed felodipine plasma concentration-time profiles (semi-logarithmic) without and during carbamazepine and phenytoin co-administration. Observed data are shown as dots ± standard deviation; model predictions are shown as solid lines. Details on dosing regimens, study population and literature reference are listed in Table S16. bid: twice daily, D: day, n: number of individuals, tab: tablet.

4.4.4 DDI AUC_{last} and C_{max} ratio goodness-of-fit plots

(a) **(b)**

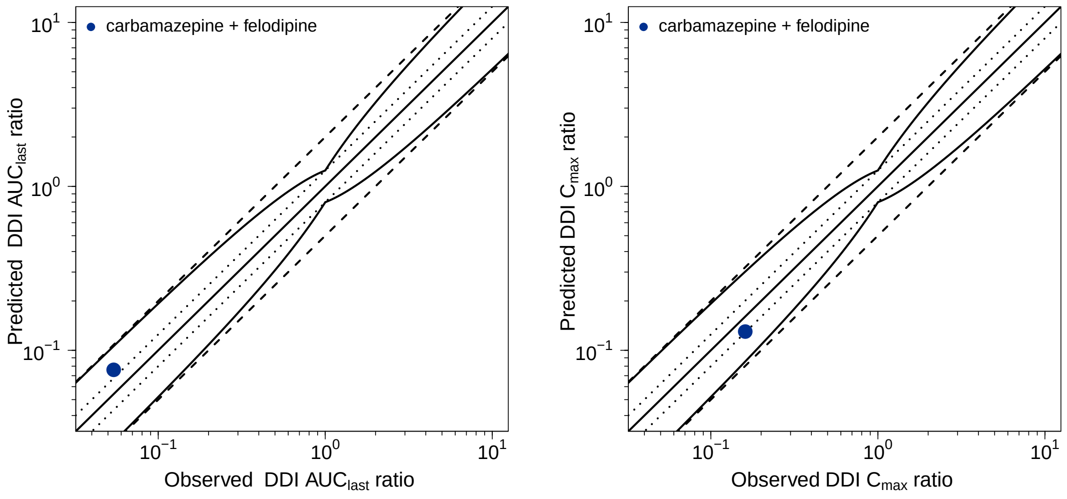

Figure S24: Predicted compared to observed carbamazepine-phenytoin-felodipine DDI AUC_{last} and DDI C_{max} values. The straight solid line marks the line of identity, the curved solid lines show the prediction success limits proposed by Guest et al. allowing for 1.25-fold variability of the DDI ratio [96]. Dotted lines indicate 1.25-fold, dashed lines indicate 2-fold deviation. AUC_{last}: area under the plasma concentration-time curve from the time of drug administration to the last concentration measurement, C_{max}: maximum plasma concentration, DDI: drug-drug interaction

4.4.5 Geometric mean fold error of predicted DDI AUC_{last} and C_{max} ratios

Table S17: Predicted and observed carbamazepine-phenytoin-felodipine DDI AUC_{last} ratios and DDI C_{max} ratios

**Perpetrator**	**Victim**	**Compound**	**Dose gap [h]**	**n**	**DDI AUC_{last} ratio**			**DDI C_{max} ratio**			
Carbamazepine/Phenytoin	Felodipine				**Pred**	**Obs**	**Pred/Obs**	**Pred**	**Obs**	**Pred/Obs**	**Reference**
757/325 mg, po, md	5 mg, po (tab), bid D1-D5	Felodipine	-	10	0.08	0.05	1.41	0.13	0.16	0.81	Capewell 1988 [23]
**GMFE**							**1.41**			**1.19**	
							**1/1 with GMFE < 2**			**1/1 with GMFE < 2**	

-: not given, AUC_{last}: area under the plasma concentration-time curve from the time of drug administration to the last concentration measurement, bid: twice daily, C_{max}: maximum plasma concentration, D: day, DDI: drug-drug interaction, GMFE: geometric mean fold error, md: multiple dose, n: number of individuals, obs: observed, po: oral, pred: predicted, tab: tablet

5 System-dependent parameters

Details on the expression of metabolizing enzymes and transport proteins implemented to model the pharmacokinetics of felodipine and dehydrofelodipine and of the perpetrator models erythromycin, itraconazole, carbamazepine and phenytoin are summarized in Table S18. As enterohepatic circulation is active under physiological conditions, the parameter EHC continuous fraction was set to 1 in all individuals.

Table S18: System-dependent parameters

	**Reference concentration**				**Half-life**	
**Enzyme/ Transporter**	**Mean[a] [µmol/l]**	**Relative expression[b]**	**Localization**	**Direction**	**Liver [h]**	**Intestine [h]**
CYP3A4	4.32 [140]	RT-PCR [141]	Intracellular	-	36 [142]	23 [143]
CYP2B6	1.56 [140]	RT-PCR [141]	Intracellular	-	32	23
CYP2C8	2.56 [140]	RT-PCR [141]	Intracellular	-	23	23
CYP2C9	3.84 [140]	RT-PCR [141]	Intracellular	-	104	23
CYP2C19	0.76 [140]	RT-PCR [141]	Intracellular	-	26	23
EPHX1	1.00[c] [139]	RT-PCR [144]	Intracellular	-	36	23
UGT2B7	2.78 [145]	EST [146]	Intracellular	-	36	23
OATP1B1	1.00[c] [139]	RT-PCR [147]	Basolateral	Influx	36	-

-: not given, CYP: cytochrome P450, EPHX1: epoxidehydroxylase 1, EST: expressed sequence tags expression profile, OATP1B1: organic anion transporting polypeptide 1B1, RT-PCR: reverse transcription-polymerase chain reaction profile, UGT: UDP-glucuronosyltransferase

[a] µmol/lol protein/l in the tissue of highest expression

[b] in the different organs (PK-Sim® expression database profile)

[c] if no information was available, the mean reference concentration was set to 1.00 µmol/l and the catalytic rate constant (kcat) was optimized [139]

List of Figures

S1 Implemented administration and elimination pathways of felodipine and dehydrofelodipine. 10
S2 Felodipine and dehydrofelodipine plasma concentration-time profiles (linear) . . . 16
S3 Felodipine and dehydrofelodipine plasma concentration-time profiles (semi-logarithmic) 25
S4 Dissolution-time profile after administration of 10 mg felodipine as extended release tablet 34
S5 Predicted compared to observed plasma concentrations of felodipine and dehydrofelodipine 35
S6 Predicted compared to observed AUC_{last} and C_{max} values of felodipine and dehydrofelodipine 39
S7 Felodipine parent-metabolite PBPK model sensitivity analysis for intravenous felodipine administration 45
S8 Felodipine parent-metabolite PBPK model sensitivity analysis for oral felodipine administration 46

S9 Effect-time profiles of diastolic blood pressure 51
S10 Effect-time profiles of heart rate 55
S11 Predicted compared to observed diastolic blood pressure and heart rate measurements 58

S12 Implementation of the erythromycin-felodipine DDI. 62
S13 Erythromycin-felodipine DDI (linear) 65
S14 Erythromycin-felodipine DDI (semi-logarithmic) 65
S15 Predicted compared to observed erythromycin-felodipine DDI AUC_{last} and DDI C_{max} values 66
S16 Implementation of the itraconazole-felodipine DDI. 68
S17 Itraconazole-felodipine DDI (linear) 72
S18 Itraconazole-felodipine DDI (semi-logarithmic) 72
S19 Itraconazole-felodipine DDI (effect-time profiles) 73
S20 Predicted compared to observed itraconazole-felodipine DDI AUC_{last} and DDI C_{max} values 74
S21 Implementation of the carbamazepine-phenytoin-felodipine DDI. 76
S22 Carbamazepine-phenytoin-felodipine DDI (linear) 80
S23 Carbamazepine-phenytoin-felodipine DDI (semi-logarithmic) 80
S24 Predicted compared to observed carbamazepine-phenytoin-felodipine DDI AUC_{last} and DDI C_{max} values 81

List of Tables

S1 Clinical studies used for the development of the felodipine PBPK/PD model . . . 11
S2 Drug-dependent parameters of the final felodipine parent-metabolite PBPK model 15
S3 Mean relative deviation values of predicted plasma concentrations of felodipine and dehydrofelodipine . . . 36
S4 Predicted and observed AUC_{last} and C_{max} values with geometric mean fold errors of felodipine and dehydrofelodipine . . . 40

S5 Optimized parameters of the circadian blood pressure and heart rate models . . 48
S6 Drug-dependent parameter of the final felodipine PD model . . . 50
S7 Mean relative deviation values of predicted diastolic blood pressure measurements with and without felodipine administration . . . 59
S8 Mean relative deviation values of predicted heart rate measurements with and without felodipine administration . . . 60

S9 Drug-dependent parameters of the erythromycin PBPK model according to [69] 63
S10 Clinical studies investigating the erythromycin-felodipine DDI . . . 64
S11 Predicted and observed erythromycin-felodipine DDI AUC_{last} ratios and DDI C_{max} ratios . . . 67
S12 Drug-dependent parameters of the itraconazole parent-metabolite PBPK model according to [97] . . . 69
S13 Clinical studies investigating the itraconazole-felodipine DDI . . . 71
S14 Predicted and observed itraconazole-felodipine DDI AUC_{last} ratios and DDI C_{max} ratios . . . 75
S15 Drug-dependent parameters of the carbamazepine and carbamazepine-10,11-epoxide PBPK model according to [106] . . . 77
S16 Clinical studies investigating the carbamazepine-phenytoin DDI . . . 79
S17 Predicted and observed carbamazepine-phenytoin-felodipine DDI AUC_{last} ratios and DDI C_{max} ratios . . . 82

S18 System-dependent parameters . . . 83

Abbreviations

**ADME**	Absorption, distribution, metabolism and excretion
**amp**	Circadian amplitude
**AUC**	Area under the concentration-time curve
**AUC_{last}**	AUC values calculated from the time of drug administration to the time of the last concentration measurement
**asm**	Assumption
**bid**	Twice daily
**BP**	Blood pressure
**calc**	Calculated
**CBZ**	Carbamazepine
**CBZE**	Carbamazepine-10,11-epoxide
**circ**	Circadian rhythm
**CL_{hep}**	Hepatic clearance
**CL**	C clearance
**C_{max}**	Maximum plasma concentration
**CYP**	Cytochrome P450
**D**	Day
**DDI**	Drug-drug interaction
**DHF**	Dehydrofelodipine
**EC50**	Concentration for half maximal induction in vivo
**EHC**	Enterohepatic circulation
**E_{max}**	Maximum induction effect in vivo
**EPHX1**	Epoxide hydroxylase 1
**EST**	Expressed sequence tag
**fu**	Fraction unbound in plasma
**GFR**	Glomerular filtration rate
**GMFE**	Geometric mean fold error
**HR**	Heart rate
**HT**	Hypertension
**IC_{50}**	Half maximal inhibitory concentration

**IR**	Immediate release
**iv**	Intravenous
k_{cat}	Transport or catalytic rate constant
k_{deg}	Degradation rate constant
$k_{deg, app}$	Degradation rate constant in the presence of a mechanism based inhibitor
K_i	Dissociation constant of the inhibitor-transporter/ -enzyme complex
K_{inact}	Maximum inactivation rate
K_M	Michaelis-Menten constant
$K_{M,app}$	Michaelis-Menten constant in the presence of inhibitor
**logP**	Lipophilicity
**lit**	Literature
**MRD**	Mean relative deviation
**md**	Multiple dose
**MW**	Molecular weight
**OATP**	Organic-anion-transporting polypeptide
**obs**	Observed
**opt**	Optimized
**OSP**	Open Systems Pharmacology
**PD**	Pharmacodynamics
**PBPK**	Physiologically based pharmacokinetic
**pKa**	Acid dissociation constant
**po**	Oral
**pred**	Predicted
**qid**	Four times daily
**qd**	Once daily
**RI**	Renal impairment
R_{syn}	Rate of transporter or enzyme synthesis
$R_{syn,app}$	Rate of transporter or enzyme synthesis in the presence of inducer
**RT-PCR**	Reverse transcription-polymerase chain reaction
**sd**	Single dose
**s**	Weibull dissolution shape
**sol**	Solution

tab Tablet

tabER Extended release tablet

UGT UDP-glucuronosyltransferase

v Reaction velocity

v_{max} Maximum reaction velocity

Bibliography

[1] Open Systems Pharmacology Suite Community. Open Systems Pharmacology Suite Manual, 2018. URL https://docs.open-systems-pharmacology.org/. accessed: 14 Dec 2020.

[2] D. Chae, Y. Kim, and K. Park. Characterization of circadian blood pressure patterns using non-linear mixed effects modeling. *Translational and clinical pharmacology*, 27(1):24–32, mar 2019.

[3] D. Lott, T. Lehr, J. Dingemanse, and A. Krause. Modeling Tolerance Development for the Effect on Heart Rate of the Selective S1P1 Receptor Modulator Ponesimod. *Clinical pharmacology and therapeutics*, 103(6):1083–1092, 2018.

[4] P. A. Soons, M. C. Roosemalen, and D. D. Breimer. Enantioselective determination of felodipine and other chiral dihydropyridine calcium entry blockers in human plasma. *Journal of chromatography*, 528(2):343–56, jun 1990.

[5] U. G. Eriksson, J. Lundahl, C. Bäärnhielm, and C. G. Regårdh. Stereoselective metabolism of felodipine in liver microsomes from rat, dog, and human. *Drug metabolism and disposition*, 19(5):889–94, 1991.

[6] B. Bengtsson-Hasselgren, B. Edgar, and O. Rönn. Dose-dependent effects of felodipine on diuresis and natriuresis in healthy subjects. *Journal of cardiovascular pharmacology*, 12(2):134–9, aug 1988.

[7] B. Edgar, C. G. Regårdh, P. Lundborg, S. Romare, G. Nyberg, and O. Rönn. Pharmacokinetic and pharmacodynamic studies of felodipine in healthy subjects after various single, oral and intravenous doses. *Biopharmaceutics & drug disposition*, 8(3):235–48, 1987.

[8] T. A. Sutfin, T. Lind, M. Gabrielsson, and C. G. Regårdh. Biliary secretion of felodipine metabolites in man after intravenous [14C] felodipine. *European journal of clinical pharmacology*, 38(5):421–4, 1990.

[9] H. E. Sluiter, F. T. Huysmans, T. A. Thien, and R. A. Koene. Haemodynamic effects of intravenous felodipine in normotensive and hypertensive subjects. *Drugs*, 29(Suppl 2): 144–53, 1985.

[10] J. Lundahl, C. G. Regårdh, B. Edgar, and G. Johnsson. Effects of grapefruit juice ingestion–pharmacokinetics and haemodynamics of intravenously and orally administered felodipine in healthy men. *European journal of clinical pharmacology*, 52(2): 139–45, 1997.

[11] B. Edgar, B. Bengtsson, D. Elmfeldt, P. Lundborg, G. Nyberg, S. Raner, and O. Rönn. Acute diuretic/natriuretic properties of felodipine in man. *Drugs*, 29(Suppl 2):176–84, 1985.

[12] B. Edgar, C. G. Regårdh, G. Johnsson, L. Johansson, P. Lundborg, I. Löfberg, and O. Rönn. Felodipine kinetics in healthy men. *Clinical pharmacology and therapeutics*, 38 (2):205–11, aug 1985.

[13] B. Abrahamsson, D. Johansson, A. Torstensson, and K. Wingstrand. Evaluation of solubilizers in the drug release testing of hydrophilic matrix extended-release tablets of felodipine. *Pharmaceutical research*, 11(8):1093–7, aug 1994.

[14] B. Bengtsson-Hasselgren, O. Rönn, L. O. Blychert, B. Edgar, and S. Raner. Acute effects of felodipine and nifedipine on hepatic and forearm blood flow in healthy men. *European journal of clinical pharmacology*, 38(6):529–33, 1990.

[15] B. Edgar, P. Lundborg, and C. G. Regårdh. Clinical pharmacokinetics of felodipine. A summary. *Drugs*, 34(Suppl 3):16–27, 1987.

[16] K. Wingstrand, B. Abrahamsson, and B. Edgar. Bioavailability from felodipine extended-release tablets with different dissolution properties. *International Journal of Pharmaceutics*, 60(2):151–156, apr 1990.

[17] E. Blychert, K. Wingstrand, B. Edgar, and K. Lidman. Plasma concentration profiles and antihypertensive effect of conventional and extended-release felodipine tablets. *British journal of clinical pharmacology*, 29(1):39–45, jan 1990.

[18] G. Johnsson, G. Murray, A. Tweddel, and I. Hutton. Haemodynamic effects of a new vasodilator drug, felodipine, in healthy subjects. *European journal of clinical pharmacology*, 24(1):49–53, 1983.

[19] P. A. Soons, T. M. Mulders, E. Uchida, H. C. Schoemaker, A. F. Cohen, and D. D. Breimer. Stereoselective pharmacokinetics of oral felodipine and nitrendipine in healthy subjects: correlation with nifedipine pharmacokinetics. *European journal of clinical pharmacology*, 44(2):163–9, 1993.

[20] D. G. Bailey, J. M. Arnold, C. Munoz, and J. D. Spence. Grapefruit juice-felodipine interaction: mechanism, predictability, and effect of naringin. *Clinical pharmacology and therapeutics*, 53(6):637–42, jun 1993.

[21] B. Edgar, D. Bailey, R. Bergstrand, G. Johnsson, and C. G. Regårdh. Acute effects of drinking grapefruit juice on the pharmacokinetics and dynamics of felodipine-and its potential clinical relevance. *European journal of clinical pharmacology*, 42(3):313–7, 1992.

[22] S. Landahl, B. Edgar, M. Gabrielsson, M. Larsson, B. Lernfelt, P. Lundborg, and C. G. Regårdh. Pharmacokinetics and blood pressure effects of felodipine in elderly hypertensive patients. A comparison with young healthy subjects. *Clinical pharmacokinetics*, 14(6):374–83, jun 1988.

[23] S. Capewell, S. Freestone, J. A. Critchley, A. Pottage, and L. F. Prescott. Reduced felodipine bioavailability in patients taking anticonvulsants. *Lancet (London, England)*, 2 (8609):480–2, aug 1988.

[24] L.-Q. Guo, Q.-Y. Chen, X. Wang, Y.-X. Liu, X.-M. Chu, X.-M. Cao, J.-H. Li, and Y. Yamazoe. Different roles of pummelo furanocoumarin and cytochrome P450 3A5*3 polymorphism in the fate and action of felodipine. *Current drug metabolism*, 8(6):623–30, aug 2007.

[25] B. Lindmark, M. Ahnoff, and B.-A. Persson. Enantioselective determination of felodipine in human plasma by chiral normal-phase liquid chromatography and electrospray ionisation mass spectrometry. *Journal of pharmaceutical and biomedical analysis*, 27(3-4): 489–95, jan 2002.

[26] B. G. Hardy, W. R. Bartle, M. Myers, D. G. Bailey, and B. Edgar. Effect of indomethacin on the pharmacokinetics and pharmacodynamics of felodipine. *British journal of clinical pharmacology*, 26(5):557–62, nov 1988.

[27] G. K. Dresser, D. G. Bailey, and S. G. Carruthers. Grapefruit juice-felodipine interaction in the elderly. *Clinical pharmacology and therapeutics*, 68(1):28–34, jul 2000.

[28] K. M. Jalava, K. T. Olkkola, and P. J. Neuvonen. Itraconazole greatly increases plasma concentrations and effects of felodipine. *Clinical pharmacology and therapeutics*, 61(4): 410–5, apr 1997.

[29] J. C. A. Carrasco, M. d. C. C. Portugal, F. J. F. Murrieta, and S. C. Quinteros. Oral Pharmacokinetics of Felodipine in Mexican Healthy Volunteers: Evidence for Interethnic Differences. *International Journal of Pharmacology*, 11(4):382–386, may 2015.

[30] T. C. Goosen, D. Cillié, D. G. Bailey, C. Yu, K. He, P. F. Hollenberg, P. M. Woster, L. Cohen, J. A. Williams, M. Rheeders, and H. P. Dijkstra. Bergamottin contribution to the grapefruit juice-felodipine interaction and disposition in humans. *Clinical pharmacology and therapeutics*, 76(6):607–17, dec 2004.

[31] Q. Xiang, C. Li, X. Zhao, and Y. M. Cui. The influence of CYP3A5*3 and BCRPC421A genetic polymorphisms on the pharmacokinetics of felodipine in healthy Chinese volunteers. *Journal of clinical pharmacy and therapeutics*, 42(3):345–349, jun 2017.

[32] A comparative study on the relative bioavailability of 2.5 and 5mg ER tablets of felodipine. NDA: 19-834, 1994. URL https://www.accessdata.fda.gov/drugsatfda_docs/nda/pre96/19834-S002_PLENDILTABLETS_BIOEQR.PDF. accessed: 02 Jun 2022.

[33] D. G. Bailey, J. M. Arnold, J. R. Bend, L. T. Tran, and J. D. Spence. Grapefruit juice-felodipine interaction: reproducibility and characterization with the extended release drug formulation. *British journal of clinical pharmacology*, 40(2):135–40, aug 1995.

[34] J. K. Madsen, J. D. Jensen, L. W. Jensen, and E. B. Pedersen. Pharmacokinetic interaction between cyclosporine and the dihydropyridine calcium antagonist felodipine. *European journal of clinical pharmacology*, 50(3):203–8, 1996.

[35] D. G. Bailey, J. R. Bend, J. M. Arnold, L. T. Tran, and J. D. Spence. Erythromycin-felodipine interaction: magnitude, mechanism, and comparison with grapefruit juice. *Clinical pharmacology and therapeutics*, 60(1):25–33, jul 1996.

[36] D. G. Bailey, J. H. Kreeft, C. Munoz, D. J. Freeman, and J. R. Bend. Grapefruit juice-felodipine interaction: effect of naringin and 6',7'-dihydroxybergamottin in humans. *Clinical pharmacology and therapeutics*, 64(3):248–56, sep 1998.

[37] D. G. Bailey, G. K. Dresser, J. H. Kreeft, C. Munoz, D. J. Freeman, and J. R. Bend. Grapefruit-felodipine interaction: effect of unprocessed fruit and probable active ingredients. *Clinical pharmacology and therapeutics*, 68(5):468–77, nov 2000.

[38] D. G. Bailey, G. K. Dresser, and J. R. Bend. Bergamottin, lime juice, and red wine as inhibitors of cytochrome P450 3A4 activity: comparison with grapefruit juice. *Clinical pharmacology and therapeutics*, 73(6):529–37, jun 2003.

[39] G. K. Dresser, B. L. Urquhart, J. Proniuk, A. Tieu, D. J. Freeman, J. M. Arnold, and D. G. Bailey. Coffee inhibition of CYP3A4 in vitro was not translated to a grapefruit-like pharmacokinetic interaction clinically. *Pharmacology research & perspectives*, 5(5):1–9, 2017.

[40] B. Hasselgren, O. Rönn, B. Edgar, P. Johansson, and B. Wall. Pharmacokinetics and hemodynamic and diuretic/natriuretic effects of felodipine administered as an extended-release tablet. *Cardiovascular drugs and therapy*, 4(6):1495–500, dec 1990.

[41] K. S. Lown, D. G. Bailey, R. J. Fontana, S. K. Janardan, C. H. Adair, L. A. Fortlage, M. B. Brown, W. Guo, and P. B. Watkins. Grapefruit juice increases felodipine oral availability

in humans by decreasing intestinal CYP3A protein expression. *The Journal of clinical investigation*, 99(10):2545–53, may 1997.

[42] J. Lundahl, C. G. Regårdh, B. Edgar, and G. Johnsson. Relationship between time of intake of grapefruit juice and its effect on pharmacokinetics and pharmacodynamics of felodipine in healthy subjects. *European journal of clinical pharmacology*, 49(1-2):61–7, 1995.

[43] J. U. Lundahl, C. G. Regårdh, B. Edgar, and G. Johnsson. The interaction effect of grapefruit juice is maximal after the first glass. *European journal of clinical pharmacology*, 54(1):75–81, mar 1998.

[44] A. Pop, L. Vlase, and Leucuta. Pharmacokinetic study of felodipine after single oral dose of slow release formulations in healthy volunteers. *Farmacia*, 56(5):474–82, 2008.

[45] A. Gelal, D. Balkan, D. Ozzeybek, Y. C. Kaplan, S. Gurler, H. Guven, and N. L. Benowitz. Effect of menthol on the pharmacokinetics and pharmacodynamics of felodipine in healthy subjects. *European journal of clinical pharmacology*, 60(11):785–90, jan 2005.

[46] W. Weitschies, R.-S. Wedemeyer, O. Kosch, K. Fach, S. Nagel, E. Söderlind, L. Trahms, B. Abrahamsson, and H. Mönnikes. Impact of the intragastric location of extended release tablets on food interactions. *Journal of controlled release : official journal of the Controlled Release Society*, 108(2-3):375–85, nov 2005.

[47] S. Patel Devang, N. Shanker, K. Shah Sweety, K. Thakkar Vaishali, N. Mehta Nirali, K. Srivstava Ambrish, S. Singh, and G. Patel Chitrang. Bioequivalence Study of Two Oral Extended Release Formulations of Felodipine 10 mg Tablets in Healthy Volunteers under Fed Condition. *Pharma Science Monitor - An International Journal Of Pharmaceutical Sciences*, 2(2, Supplement 1):9–20, 2011.

[48] S. R. Smith, M. R. Wilkins, D. B. Jack, M. J. Kendall, and S. Laugher. Pharmacokinetic interactions between felodipine and metoprolol. *European journal of clinical pharmacology*, 31(5):575–8, 1987.

[49] B. Edgar, C. G. Regårdh, P. O. Attman, M. Aurell, H. Herlitz, and G. Johnsson. Pharmacokinetics of felodipine in patients with impaired renal function. *British journal of clinical pharmacology*, 27(1):67–74, jan 1989.

[50] E. Blychert, T. Hedner, C. Dahlöf, and D. Elmfeldt. Plasma concentration-effect relationships of intravenous and extended-release oral felodipine in hypertensive patients. *Journal of cardiovascular pharmacology*, 15(3):428–35, mar 1990.

[51] R. Larsson, B. E. Karlberg, A. Gelin, J. Aberg, and C. G. Regårdh. Acute and steady-state pharmacokinetics and antihypertensive effects of felodipine in patients with normal and impaired renal function. *Journal of clinical pharmacology*, 30(11):1020–30, nov 1990.

[52] T. Hedner, D. Elmfeldt, C. Dahlöf, and E. Sjögren. Comparison of antihypertensive effect and pharmacokinetics of conventional and extended release felodipine tablets in patients with arterial hypertension. *Drugs*, 34(Suppl 3):125–31, 1987.

[53] F. H. H. Leenen and E. Coletta. Pharmacokinetic and antihypertensive profile of amlodipine and felodipine-ER in younger versus older patients with hypertension. *Journal of cardiovascular pharmacology*, 56(6):669–75, dec 2010.

[54] T. Hedner, O. Samuelsson, E. Sjögren, and D. Elmfeldt. Treatment of essential hypertension with felodipine in combination with a diuretic. *European journal of clinical pharmacology*, 30(2):133–9, 1986.

[55] Human Metabolome Database. Metabocard for Felodipine (HMDB0015158). URL https://hmdb.ca/metabolites/HMDB0015158. accessed: 02 Jun 2022.

[56] C. G. Regårdh, B. Edgar, R. Olsson, M. Kendall, P. Collste, and C. Shansky. Pharmacokinetics of felodipine in patients with liver disease. *European journal of clinical pharmacology*, 36(5):473–9, 1989.

[57] K. Felle, B. Persson, and J. Vessman. Dissolution test for felodipine tablets using chemical oxidation in situ to maintain 'sink conditions'. *Journal of Pharmaceutical and Biomedical Analysis*, 2(3/4):527–536, jan 1984.

[58] DRUGBANK online - Felodipine, 2022. URL https://go.drugbank.com/drugs/DB01023. accessed: 02 Jun 2022.

[59] J. Takano, K. Maeda, M. B. Bolger, and Y. Sugiyama. The Prediction of the Relative Importance of CYP3A/P-glycoprotein to the Nonlinear Intestinal Absorption of Drugs by Advanced Compartmental Absorption and Transit Model. *Drug metabolism and disposition: the biological fate of chemicals*, 44(11):1808–1818, 2016.

[60] R. van der Lee, M. Pfaffendorf, R. P. Koopmans, J. J. van Lieshout, G. A. van Montfrans, and P. A. van Zwieten. Comparison of the time courses and potencies of the vasodilator effects of nifedipine and felodipine in the human forearm. *Blood pressure*, 10(4):217–22, 2001.

[61] P. Berben, J. Brouwers, and P. Augustijns. Assessment of Passive Intestinal Permeability Using an Artificial Membrane Insert System. *Journal of pharmaceutical sciences*, 107 (1):250–256, 2018.

[62] R. L. Walsky and R. S. Obach. Validated assays for human cytochrome P450 activities. *Drug metabolism and disposition: the biological fate of chemicals*, 32(6):647–60, jun 2004.

[63] A. Galetin, S. E. Clarke, and J. B. Houston. Quinidine and haloperidol as modifiers of CYP3A4 activity: multisite kinetic model approach. *Drug metabolism and disposition: the biological fate of chemicals*, 30(12):1512–22, dec 2002.

[64] T. Rodgers, D. Leahy, and M. Rowland. Physiologically based pharmacokinetic modeling 1: predicting the tissue distribution of moderate-to-strong bases. *Journal of pharmaceutical sciences*, 94(6):1259–76, jun 2005.

[65] M. J. Taylor, S. Tanna, and T. Sahota. In vivo study of a polymeric glucose-sensitive insulin delivery system using a rat model. *Journal of pharmaceutical sciences*, 99(10): 4215–27, 2010.

[66] R. Kawai, M. Lemaire, J. L. Steimer, A. Bruelisauer, W. Niederberger, and M. Rowland. Physiologically based pharmacokinetic study on a cyclosporin derivative, SDZ IMM 125. *Journal of pharmacokinetics and biopharmaceutics*, 22(5):327–65, oct 1994.

[67] Chemaxon. Chemicalize - Dehydrofelodipine, 2022. URL https://chemicalize.com/welcome.

[68] W. Schmitt. General approach for the calculation of tissue to plasma partition coefficients. *Toxicology in vitro : an international journal published in association with BIBRA*, 22(2): 457–67, mar 2008.

[69] S. Frechen and A. Dallmann. Building and evaluation of a PBPK model for erythromycin in healthy adults, 2020. URL https://github.com/Open-Systems-Pharmacology/OSP-PBPK-Model-Library/blob/v9.1/Erythromycin/Erythromycin_evaluation_report.pdf. accessed: 02 Jun 2022.

[70] S. Frechen. CYP3A4 DDI Qualification, 2020. URL https://github.com/Open-Systems-Pharmacology/OSP-Qualification-Reports/blob/v9.1/DDI_Qualification_CYP3A4/report.pdf. accessed: 02 Jun 2022.

[71] Drugbank. Erythromycin, 2020. URL https://go.drugbank.com/drugs/DB00199. accessed: 02 Jun 2022.

[72] E. Lien, J. Kuwahara, and R. Koda. Diffusion of drugs into prostatic fluid and milk. *Drug intelligence & Clinical Pharmacy*, 8(8):470–475, 1974.

[73] J. O. Capobianco and R. C. Goldman. Macrolide transport in Escherichia coli strains having normal and altered OmpC and/or OmpF porins. *International journal of antimicrobial agents*, 4(3):183–9, 1994.

[74] J. W. McFarland, C. M. Berger, S. A. Froshauer, S. F. Hayashi, S. J. Hecker, B. H. Jaynes, M. R. Jefson, B. J. Kamicker, C. A. Lipinski, K. M. Lundy, C. P. Reese, and C. B. Vu. Quantitative structure-activity relationships among macrolide antibacterial agents: In vitro and in vivo potency against Pasteurella multocida. *Journal of Medicinal Chemistry*, 40 (9):1340–1346, 1997.

[75] C. Hoffhine. Aqueous soluble salts of erythromycin. Issues September 4. Nr. 2,761,859, 1956. URL https://patents.google.com/patent/US2761859A/en. accessed: 02 Jun 2022.

[76] P. H. Jones, E. K. Rowley, A. L. Weiss, D. L. Bishop, and A. H. Chun. Insoluble erythromycin salts. *Journal of Pharmaceutical Sciences*, 58(3):337–339, 1969.

[77] P. K. Manna and S. K. Basu. Preparation and evaluation of erythromycin fumarate - A new derivative of erythromycin. *Drug Development and Industrial Pharmacy*, 24(9): 879–882, 1998.

[78] H. Sun, L. A. Frassetto, Y. Huang, and L. Z. Benet. Hepatic clearance, but not gut availability, of erythromycin is altered in patients with end-stage renal disease. *Clinical Pharmacology & Therapeutics*, 87(4):465–472, 2010.

[79] A. Iliopoulou, M. Aldhous, A. Johnston, and P. Turner. Pharmacokinetic interaction between theophylline and erythromycin. *British Journal of Clinical Pharmacology*, 14 (4):495–499, 1982.

[80] J. Barre, A. Mallat, J. Rosenbaum, L. Deforges, G. Houin, D. Dhumeaux, and J. Tillement. Pharmacokinetics of erythromycin in patients with severe cirrhosis. Respective influence of decreased serum binding and impaired liver metabolic capacity. *British Journal of Clinical Pharmacology*, 23(6):753–757, 1987.

[81] L. Xu, Y. Chen, Y. Pan, G. L. Skiles, and M. Shou. Prediction of human drug-drug interactions from time-dependent inactivation of CYP3A4 in primary hepatocytes using a population-based simulator. *Drug Metabolism and Disposition*, 37(12):2330–2339, 2009.

[82] R. W. Wang, D. J. Newton, T. D. Scheri, and A. Y. Lu. Human cytochrome P450 3A4-catalyzed testosterone 6 beta-hydroxylation and erythromycin N-demethylation. Competition during catalysis. *Drug metabolism and disposition: the biological fate of chemicals*, 25(4):502–7, 1997.

[83] R. J. Riley and D. Howbrook. In vitro analysis of the activity of the major human hepatic CYP enzyme (CYP3A4) using [N-methyl-14C]-erythromycin. *Journal of Pharmacological and Toxicological Methods*, 38(4):189–193, dec 1997.

[84] C. S. Lancaster, G. H. Bruun, C. J. Peer, T. S. Mikkelsen, T. J. Corydon, A. A. Gibson, S. Hu, S. J. Orwick, R. H. J. Mathijssen, W. D. Figg, S. D. Baker, and A. Sparreboom. OATP1B1 polymorphism as a determinant of erythromycin disposition. *Clinical Pharmacology & Therapeutics*, 92(5):642–650, nov 2012.

[85] T. Akiyoshi, M. Ito, S. Murase, M. Miyazaki, F. P. Guengerich, K. Nakamura, K. Yamamoto, and H. Ohtani. Mechanism-based inhibition profiles of erythromycin and clarithromycin with cytochrome P450 3A4 genetic variants. *Drug metabolism and pharmacokinetics*, 28 (5):411–5, 2013.

[86] A. Atkinson, J. R. Kenny, and K. Grime. Automated assessment of time-dependent inhibition of human cytochrome P450 enzymes using liquid chromatography-tandem mass spectrometry analysis. *Drug Metabolism and Disposition*, 33(11):1637–1647, nov 2005.

[87] S. Aueviriyavit, K. Kobayashi, and K. Chiba. Species differences in mechanism-based inactivation of CYP3A in humans, rats and mice. *Drug Metabolism and Pharmacokinetics*, 25(1):93–100, 2010.

[88] W. K. Chan and A. B. Delucchi. Resveratrol, a red wine constituent, is a mechanism-based inactivator of cytochrome P450 3A4. *Life Sciences*, 67(25):3103–3112, nov 2000.

[89] Y. Chen, L. Liu, M. Monshouwer, and A. J. Fretland. Determination of time-dependent inactivation of CYP3A4 in cryopreserved human hepatocytes and assessment of human drug-drug interactions. *Drug Metabolism and Disposition*, 39(11):2085–2092, nov 2011.

[90] Y. Ishikawa, T. Akiyoshi, A. Imaoka, and H. Ohtani. Inactivation kinetics and residual activity of CYP3A4 after treatment with erythromycin. *Biopharmaceutics & Drug Disposition*, 38(7):420–425, 2017.

[91] S. Kanamitsu, I. K, G. CE, C. Tyson, N. Shimada, and Y. Sugiyama. Prediction of in vivo interaction between triazolam and erythromycin based on in vitro studies using human liver microsomes and recombinant human CYP3A4. *Pharmaceutical Research*, 17(4): 419–426, 2000.

[92] K. Kozakai, Y. Yamada, M. Oshikata, T. Kawase, E. Suzuki, Y. Haramaki, and H. Taniguchi. Cocktail-substrate approach-based high-throughput assay for evaluation of direct and time-dependent inhibition of multiple cytochrome P450 isoforms. *Drug Metabolism and Pharmacokinetics*, 29(2):198–207, 2014.

[93] J. Mao, S. Tay, C. S. Khojasteh, Y. Chen, C. E. C. A. Hop, and J. R. Kenny. Evaluation of time dependent inhibition assays for marketed oncology drugs: Comparison of human hepatocytes and liver microsomes in the presence and absence of human plasma. *Pharmaceutical Research*, 33(5):1204–1219, 2016.

[94] D. J. McConn, Y. S. Lin, K. Allen, K. L. Kunze, and K. E. Thummel. Differences in the inhibition of cytochromes P450 3A4 and 3A5 by metabolite-inhibitor complex-forming drugs. *Drug Metabolism and Disposition*, 32(10):1083–1091, 2004.

[95] X. Zhang, D. R. Jones, and S. D. Hall. Prediction of the effect of erythromycin, diltiazem, and their metabolites, alone and in combination, on CYP3A4 inhibition. *Drug Metabolism and Disposition*, 37(1):150–160, 2009.

[96] E. J. Guest, L. Aarons, J. B. Houston, A. Rostami-Hodjegan, and A. Galetin. Critique of the two-fold measure of prediction success for ratios: application for the assessment of drug-drug interactions. *Drug metabolism and disposition: the biological fate of chemicals*, 39(2):170–3, feb 2011.

[97] N. Hanke, S. Frechen, D. Moj, H. Britz, T. Eissing, T. Wendl, and T. Lehr. PBPK Models for CYP3A4 and P-gp DDI prediction: a modeling network of rifampicin, itraconazole, clarithromycin, midazolam, alfentanil, and digoxin. *CPT: pharmacometrics & systems pharmacology*, 7(10):647–59, oct 2018.

[98] J. Heykants, A. Van Peer, V. Van de Velde, P. Van Rooy, W. Meuldermans, K. Lavrijsen, R. Woestenborghs, J. Van Cutsem, and G. Cauwenbergh. The clinical pharmacokinetics of itraconazole: an overview. *Mycoses*, 32 Suppl 1:67–87, 1989.

[99] I. E. Templeton, K. E. Thummel, E. D. Kharasch, K. L. Kunze, C. Hoffer, W. L. Nelson, and N. Isoherranen. Contribution of itraconazole metabolites to inhibition of CYP3A4 in vivo. *Clinical pharmacology and therapeutics*, 83(1):77–85, jan 2008.

[100] K. Riccardi, S. Cawley, P. D. Yates, C. Chang, C. Funk, M. Niosi, J. Lin, and L. Di. Plasma Protein Binding of Challenging Compounds. *Journal of pharmaceutical sciences*, 104(8): 2627–36, aug 2015.

[101] M. Ishigam, M. Uchiyama, T. Kondo, H. Iwabuchi, S. I. Inoue, W. Takasaki, T. Ikeda, T. Komai, K. Ito, and Y. Sugiyama. Inhibition of in vitro metabolism of simvastatin by itraconazole in humans and prediction of in vivo drug-drug interactions. *Pharmaceutical Research*, 18(5):622–631, 2001.

[102] N. Isoherranen, K. L. Kunze, K. E. Allen, W. L. Nelson, and K. E. Thummel. Role of itraconazole metabolites in CYP3A4 inhibition. *Drug metabolism and disposition: the biological fate of chemicals*, 32(10):1121–31, oct 2004.

[103] National Center for Biotechnology Information (NCBI). Hydroxy-itraconazole - PubChem Identifier: CID 108222. PubChem Database., . URL https://pubchem.ncbi.nlm.nih.gov/compound/108222. accessed: 02 Jun 2022.

[104] National Center for Biotechnology Information (NCBI). Keto-itraconazole - PubChem Identifier: CID 45039625. PubChem Database., . URL https://pubchem.ncbi.nlm.nih.gov/compound/45039625. accessed: 02 Jun 2022.

[105] National Center for Biotechnology Information (NCBI). N-Desalkyl-itraconazole - PubChem Identifier: CID 53789808. PubChem Database., . URL https://pubchem.ncbi.nlm.nih.gov/compound/53789808. accessed: 02 Jun 2022.

[106] L. M. Fuhr, F. Z. Marok, N. Hanke, D. Selzer, and T. Lehr. Pharmacokinetics of the CYP3A4 and CYP2B6 Inducer Carbamazepine and Its Drug-Drug Interaction Potential: A Physiologically Based Pharmacokinetic Modeling Approach. *Pharmaceutics*, 13(2): 1–21, feb 2021.

[107] A. G. Staines, M. W. Coughtrie, and B. Burchell. N-glucuronidation of carbamazepine in human tissues is mediated by UGT2B7. *Journal of Pharmacology and Experimental Therapeutics*, 311(3):1131–1137, 2004.

[108] B. Achour, M. R. Russell, J. Barber, and A. Rostami-Hodjegan. Simultaneous quantification of the abundance of several cytochrome P450 and uridine 5-diphospho-glucuronosyltransferase enzymes in human liver microsomes using multiplexed targeted proteomics. *Drug Metabolism and Disposition*, 42(4):500–510, 2014.

[109] Drugbank. Carbamazepine, 2018. URL https://www.drugbank.ca/drugs/DB00564. accessed: 02 Jun 2022.

[110] R. P. Austin, P. Barton, S. L. Cockroft, M. C. Wenlock, and R. J. Riley. The influence of nonspecific microsomal binding on apparent intrinsic clearance, and its prediction from physicochemical properties. *Drug Metabolism and Disposition*, 30(12):1497–1503, 2002.

[111] A. Avdeef. *Absorption and drug development - Solubility, permeability, and charge state*. 2003. ISBN 3175723993.

[112] P. Annaert, Z. Ye, B. Stieger, and P. Augustijns. Interaction of HIV protease inhibitors with OATP1B1, 1B3, and 2B1. *Xenobiotica*, 40(3):163–176, 2010.

[113] S. Clarysse, J. Brouwers, J. Tack, P. Annaert, and P. Augustijns. Intestinal drug solubility estimation based on simulated intestinal fluids: Comparison with solubility in human intestinal fluids. *European Journal of Pharmaceutical Sciences*, 43(4):260–269, 2011.

[114] T. Heikkilä, M. Karjalainen, K. Ojala, K. Partola, F. Lammert, P. Augustijns, A. Urtti, M. Yliperttula, L. Peltonen, and J. Hirvonen. Equilibrium drug solubility measurements in 96-well plates reveal similar drug solubilities in phosphate buffer pH 6.8 and human intestinal fluid. *International Journal of Pharmaceutics*, 405(1-2):132–136, 2011.

[115] E. Söderlind, E. Karlsson, A. Carlsson, R. Kong, A. Lenz, S. Lindborg, and J. J. Sheng. Simulating fasted human intestinal fluids: understanding the roles of lecithin and bile acids. *Molecular pharmaceutics*, 7(5):1498–507, oct 2010.

[116] Heumann Pharma GmbH & Co. Generica KG. Fachinformation - Carbamazepin 200/400 Heumann, 2014. URL https://www.fachinfo.de/suche/fi/006667. accessed: 11 Nov 2020.

[117] Novartis. Tegretol ® label, 2009. URL https://www.accessdata.fda.gov/drugsatfda_docs/label/2009/016608s101,018281s048lbl.pdf. accessed: 02 Jun 2022.

[118] S. Pynnönen. The Pharmacokinetics of Carbamazepine in Plasma and Saliva of Man. *Acta Pharmacologica et Toxicologica*, 41(5):465–471, 1977.

[119] L. Bertilsson. Clinical pharmacokinetics of carbamazepine. *Clinical Pharmacokinetics*, 3:128–1473, 1978.

[120] B. M. Kerr, K. E. Thummel, C. J. Wurden, S. M. Klein, D. L. Kroetz, F. J. Gonzalez, and R. H. Levy. Human liver carbamazepine metabolism. Role of CYP3A4 and CYP2C8 in 10,11-epoxide formation. *Biochemical pharmacology*, 47(11):1969–79, jun 1994.

[121] J. Henshall, A. Galetin, A. Harrison, and J. B. Houston. Comparative analysis of CYP3A heteroactivation by steroid hormones and flavonoids in different in vitro systems and potential in vivo implications. *Drug Metabolism and Disposition*, 36(7):1332–1340, 2008.

[122] N. Cazali, A. Tran, J. M. Treluyer, E. Rey, P. Athis, J. Vincent, and G. Pons. Inhibitory effect of stiripentol on carbamazepine and saquinavir metabolism in human. *British Journal of Clinical Pharmacology*, 56(5):526, 2003.

[123] W. Huang, Y. S. Lin, D. J. McConn, J. C. Calamia, R. A. Totah, N. Isoherranen, M. Glodowski, and K. E. Thummel. Evidence of significant contribution from CYP3A5 to hepatic drug metabolism. *Drug Metabolism and Disposition*, 32(12):1434–1445, 2004.

[124] R. E. Pearce, G. R. Vakkalagadda, and J. Steven Leeder. Pathways of carbamazepine bioactivation in vitro I. Characterization of human cytochromes P450 responsible for the formation of 2- and 3-hydroxylated metabolites. *Drug Metabolism and Disposition*, 30 (11):1170–1179, 2002.

[125] M. Shou, M. Hayashi, Y. Pan, Y. Xu, K. Morrissey, L. Xu, and G. L. Skiles. Modeling, prediction, and in vitro in vivo correlation of CYP3A4 induction. *Drug metabolism and disposition: the biological fate of chemicals*, 36(11):2355–70, 2008.

[126] D. F. McGinnity, G. Zhang, J. R. Kenny, G. A. Hamilton, S. Otmani, K. R. Stams, S. Haney, P. Brassil, D. M. Stresser, and R. J. Riley. Evaluation of multiple in vitro systems for assessment of CYP3A4 induction in drug discovery: human hepatocytes, pregnane X receptor reporter gene, and Fa2N-4 and HepaRG cells. *Drug metabolism and disposition: the biological fate of chemicals*, 37(6):1259–68, jun 2009.

[127] O. A. Fahmi, J. L. Raucy, E. Ponce, S. Hassanali, and J. M. Lasker. Utility of DPX2 cells for predicting CYP3A induction-mediated drug-drug interactions and associated structure-activity relationships. *Drug metabolism and disposition: the biological fate of chemicals*, 40(11):2204–11, nov 2012.

[128] J. G. Zhang, T. Ho, A. L. Callendrello, R. J. Clark, E. A. Santone, S. Kinsman, D. Xiao, L. G. Fox, H. J. Einolf, and D. M. Stresser. Evaluation of calibration curve-based approaches to predict clinical inducers and noninducers of CYP3A4 with plated human hepatocytes. *Drug Metabolism and Disposition*, 42(9):1379–1391, 2014.

[129] A. Moore, P. P. Chothe, H. Tsao, and N. Hariparsad. Evaluation of the interplay between uptake transport and CYP3A4 induction in micropatterned cocultured hepatocytes. *Drug Metabolism and Disposition*, 44(12):1910–1919, 2016.

[130] O. A. Fahmi, M. Kish, S. Boldt, and R. Scott Obach. Cytochrome P450 3A4 mRNA is a more reliable marker than CYP3A4 activity for detecting pregnane X receptor-activated induction of drug-metabolizing enzymes. *Drug Metabolism and Disposition*, 38(9):1605–1611, 2010.

[131] R. Zuo, F. Li, S. Parikh, L. Cao, K. L. Cooper, Y. Hong, J. Liu, R. A. Faris, D. Li, and H. Wang. Evaluation of a novel renewable hepatic cell model for prediction of clinical CYP3A4 induction using a correlation-based relative induction score approach. *Drug Metabolism and Disposition*, 45(2):198–207, 2017.

[132] J. G. Zhang, R. Patel, R. J. Clark, T. Ho, S. K. Trisdale, Y. Fang, and D. M. Stresser. Effect of Fifteen CYP3A4 in vitro Inducers on the Induction of Hepatocytes : A Trend Analysis. Poster presented at: 20th North American ISSX Meeting; 2015 18-22 Oct; Orlando Florida.

[133] O. A. Fahmi, M. Shebley, J. Palamanda, M. W. Sinz, D. Ramsden, H. J. Einolf, L. Chen, and H. Wang. Evaluation of CYP2B6 induction and prediction of clinical drug-drug interactions: Considerations from the IQ consortium induction working group - An industry perspective. *Drug Metabolism and Disposition*, 44(10):1720–1730, 2016.

[134] L. J. Dickmann and N. Isoherranen. Quantitative prediction of CYP2B6 induction by estradiol during pregnancy: Potential explanation for increased methadone clearance during pregnancy. *Drug Metabolism and Disposition*, 41(2):270–274, 2013.

[135] O. Pelkonen, P. Myllynen, P. Taavitsainen, A. R. Boobis, P. Watts, B. G. Lake, R. J. Price, A. B. Renwick, M. J. Gómez-Lechón, J. V. Castell, M. Ingelman-Sundberg, M. Hidestrand, A. Guillouzo, L. Corcos, P. S. Goldfarb, and D. F. Lewis. Carbamazepine: A 'blind' assessment of CYP-associated metabolism and interactions in human liver-derived in vitro systems. *Xenobiotica*, 31(6):321–343, 2001.

[136] H. Lennernäs. Intestinal permeability and its relevance for absorption and elimination. *Xenobiotica*, 37(10-11):1015–1051, nov 2007.

[137] Drugbank. Metabolite 10,11-Epoxycarbamazepine, 2020. URL https://www.drugbank.ca/metabolites/DBMET00291. accessed: 02 Jun 2022.

[138] P. L. Morselli, M. Gerna, D. de Maio, G. Zanda, F. Viani, and S. Garattini. *Pharmacokinetic studies on carbamazepine in volunteers and in epileptic patients*. Springer Berlin Heidelberg, Berlin, Heidelberg, 1975. ISBN 978-3-642-85923-6.

[139] M. Meyer, S. Schneckener, B. Ludewig, L. Kuepfer, and J. Lippert. Using expression data for quantification of active processes in physiologically based pharmacokinetic modeling. *Drug metabolism and disposition: the biological fate of chemicals*, 40(5):892–901, may 2012.

[140] A. D. Rodrigues. Integrated cytochrome P450 reaction phenotyping: attempting to bridge the gap between cDNA-expressed cytochromes P450 and native human liver microsomes. *Biochemical pharmacology*, 57(5):465–80, 1999.

[141] M. Nishimura, H. Yaguti, H. Yoshitsugu, S. Naito, and T. Satoh. Tissue distribution of mRNA expression of human cytochrome P450 isoforms assessed by high-sensitivity real-time reverse transcription PCR. *Journal of the Pharmaceutical Society of Japan*, 123(5):369–75, may 2003.

[142] K. Rowland Yeo, R. L. Walsky, M. Jamei, A. Rostami-Hodjegan, and G. T. Tucker. Prediction of time-dependent CYP3A4 drug-drug interactions by physiologically based pharmacokinetic modelling: Impact of inactivation parameters and enzyme turnover. *European Journal of Pharmaceutical Sciences*, 43(3):160–73, 2011.

[143] D. J. Greenblatt, L. L. Von Moltke, J. S. Harmatz, G. Chen, J. L. Weemhoff, C. Jen, C. J. Kelley, B. W. LeDuc, and M. A. Zinny. Time course of recovery of cytochrome P450 3A function after single doses of grapefruit juice. *Clinical Pharmacology and Therapeutics*, 74(2):121–29, 2003.

[144] M. Nishimura and S. Naito. Tissue-specific mRNA expression profiles of human phase I metabolizing enzymes except for cytochrome P450 and phase II metabolizing enzymes. *Drug metabolism and pharmacokinetics*, 21(5):357–74, 2006.

[145] G. Margaillan, M. Rouleau, K. Klein, J. K. Fallon, P. Caron, L. Villeneuve, P. C. Smith, U. M. Zanger, and C. Guillemette. Multiplexed targeted quantitative proteomics predicts hepatic glucuronidation potential. *Drug Metabolism and Disposition*, 43:1331–5, 2015.

[146] National Center for Biotechnology Information (NCBI). Expressed Sequence Tags (EST) from UniGene. 2019.

[147] M. Nishimura and S. Naito. Tissue-specific mRNA expression profiles of human ATP-binding cassette and solute carrier transporter superfamilies. *Drug metabolism and pharmacokinetics*, 20(6):452–77, 2005.
